# Synthesis and Fluorescent Properties of Alkynyl- and Alkenyl-Fused Benzotriazole-Derived α-Amino Acids

**DOI:** 10.1021/acs.joc.2c02886

**Published:** 2023-02-07

**Authors:** Leanne M. Riley, Toni N. Mclay, Andrew Sutherland

**Affiliations:** School of Chemistry, The Joseph Black Building, University of Glasgow, Glasgow G12 8QQ, U.K.

## Abstract

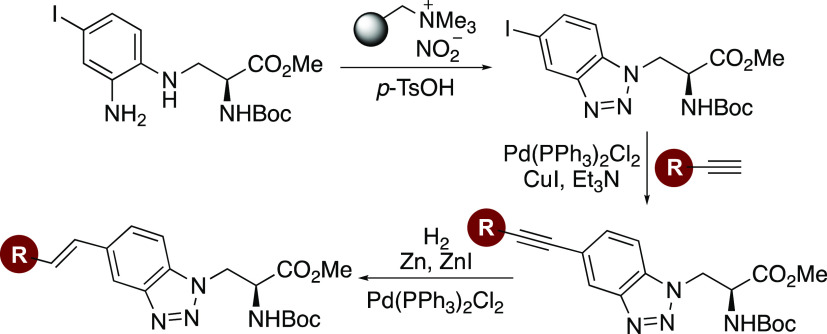

Fluorescent unnatural α-amino acids are widely used as probes in chemical biology and medicinal chemistry. While a variety of structural classes have been developed, there is still a requirement for new environmentally sensitive analogues that can closely mimic proteinogenic α-amino acids. Here, we report the synthesis and fluorescent properties of highly conjugated, benzotriazole-derived α-amino acids designed to mimic l-tryptophan. Alkynyl-substituted analogues were prepared using three key steps, nucleophilic aromatic substitution with a 3-aminoalanine derivative, benzotriazole formation *via* a one-pot diazotization and cyclization process, and a Sonogashira cross-coupling reaction. *E*-Alkenyl-substituted benzotriazoles were accessed by stereoselective partial hydrogenation of the alkynes using zinc iodide and palladium catalysis. The alkynyl analogues were found to possess higher quantum yields and stronger brightness and, a solvatochromic study with the most fluorogenic α-amino acids demonstrated sensitivity to polarity.

## Introduction

**Figure 1:**
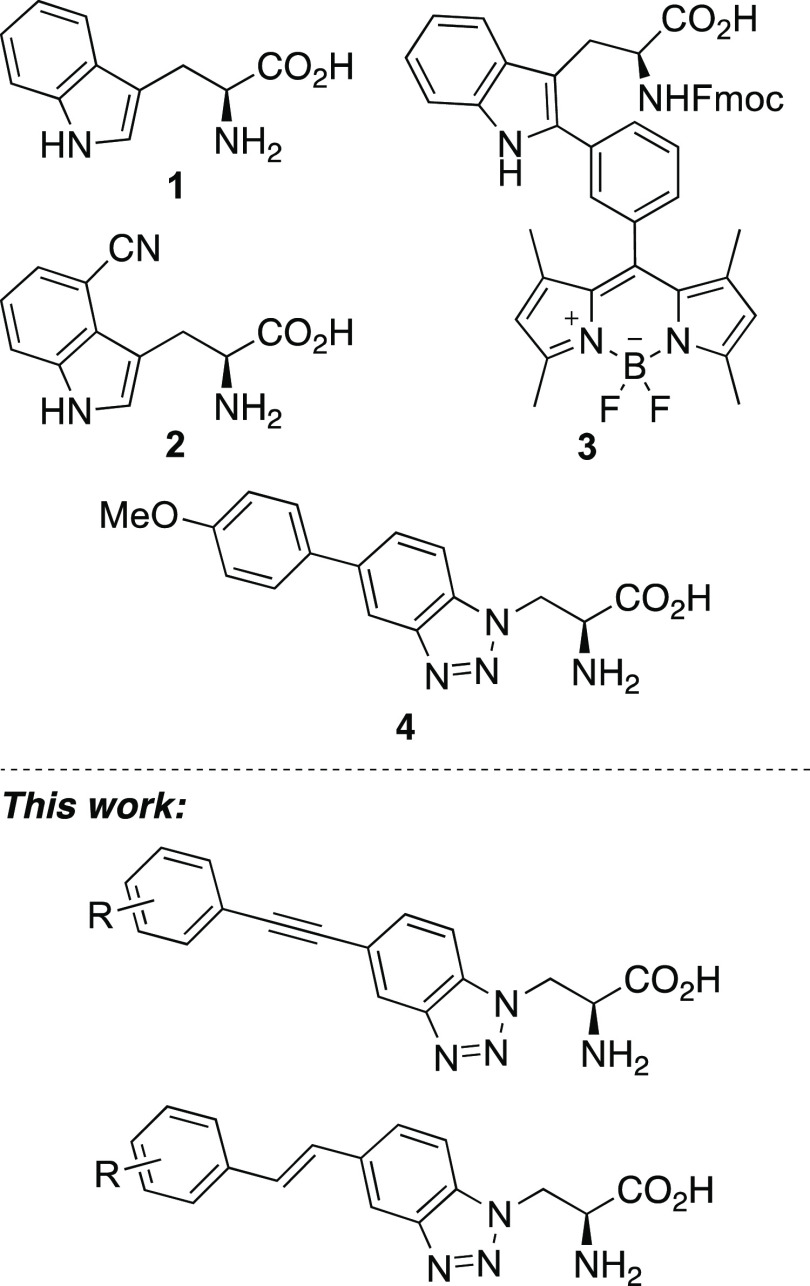
l-Tryptophan (**1**) and selected fluorescent unnatural α-amino acid mimics.

Fluorescence spectroscopy has become a powerful technique for the investigation of biological structure and function, and for visualizing cellular processes at the molecular level.^1^ In combination with the advances in fluorescent-based technology, libraries of small-molecule probes containing chromophores that are tuned for specific applications have been reported.^2^ As peptides and proteins are important for a wide range of biological processes, there has been significant interest in the discovery of fluorescent, unnatural α-amino acids that can be specifically incorporated into a protein, while retaining the original structure and function.^3^ A major strategy in the development of novel, fluorescent α-amino acids has been the structural modification of l-tryptophan (**1**), the most fluorescent proteinogenic α-amino acid (Figure 1). To improve the intrinsic optical properties of the indole side chain and to prevent spectroscopic overlap with l-tryptophan residues already present in a protein, studies have focused on the preparation of analogues with extended conjugation.^4^ For example, cyanotryptophans have proven to be excellent structural analogues of l-tryptophan, with significantly improved optical properties.^5^l-6-Cyanotryptophan has been used as a fluorescent probe for studying protein conformational changes,^6^ while the blue fluorescent amino acid l-4-cyanotryptophan (**2**) has been used to assess peptide–membrane interactions.^7^ Tryptophan compounds with extended conjugation at the C-2 position of the indole have also been prepared *via* coupling reactions with triazoles or arenes.^8^ This general approach has been used by Vendrell and co-workers, who have developed a series of l-tryptophan analogues substituted at the C-2 position with BODIPY chromophores.^9,10^ This includes l-tryptophan-BODIPY conjugate **3** that was incorporated into a cyclic peptide and used for the visualization of fungal infections in human tissue.^9^

We have previously reported the synthesis and fluorescent properties of various classes of α-amino acids,^11^ including the preparation of benzotriazole-derived α-amino acids, as potential structural mimics of l-tryptophan.^12^ In a previous study, a Suzuki–Miyaura cross-coupling reaction was used to extend the conjugation of the benzotriazole side chain allowing access to 5-aryl analogues such as compound **4** (Figure 1). Incorporation of various electron-rich arenes generated fluorescent amino acids with MegaStokes shifts. However, the main absorption band of these compounds was similar to that of proteinogenic α-amino acids, such as l-tyrosine and l-tryptophan, and thus, prohibited the use of these as fluorescent probes in proteins containing these residues. To overcome this limitation, we were interested in the development of new analogues with extended conjugation that would exhibit absorption at longer wavelengths. Here, we report the design and synthesis of a new class of fluorescent α-amino acid that incorporates an alkynyl or alkenyl spacer unit between the benzotriazole and aryl groups. We also describe the fluorescent properties of these compounds and demonstrate that as well as possessing absorption at a longer wavelength, key analogues from the alkynyl series are brighter and highly sensitive to environment polarity.

## Results and Discussion

**Scheme 1:**
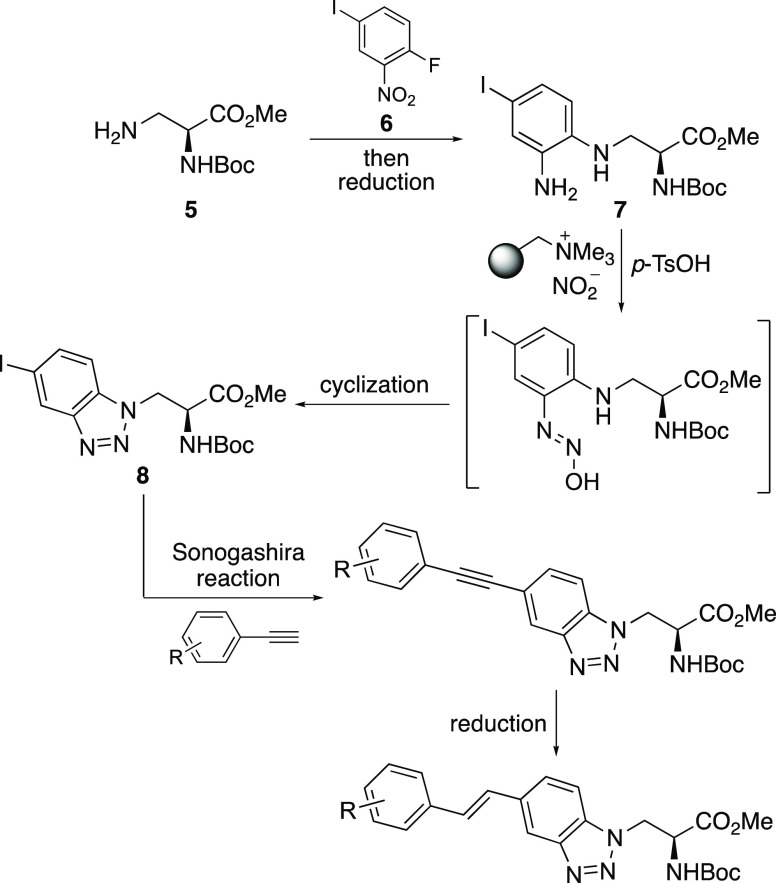
Proposed Synthesis of α-Amino Acid Targets

As shown in Scheme 1, our proposed approach to α-amino acids bearing alkynyl- and alkenyl-substituted benzotriazole side chains involved the synthesis of 5-iodobenzotriazole **8** as a key intermediate. The planned three-step preparation of **8** involved a nucleophilic aromatic substitution reaction of 2-fluoro-5-iodonitrobenzene (**6**) with 3-aminoalanine derivative **5**. Following reduction of the nitro group, a one-pot diazotization and cyclization reaction under mild conditions would yield 5-iodobenzotriazole **8**. Sonogashira coupling of **8** with a range of aryl-substituted alkynes would result in the preparation of the alkynyl targets. Stereo- and chemoselective reduction of the alkynyl compounds would then allow the rapid synthesis of the second set of targets, the styryl analogues.

**Scheme 2:**
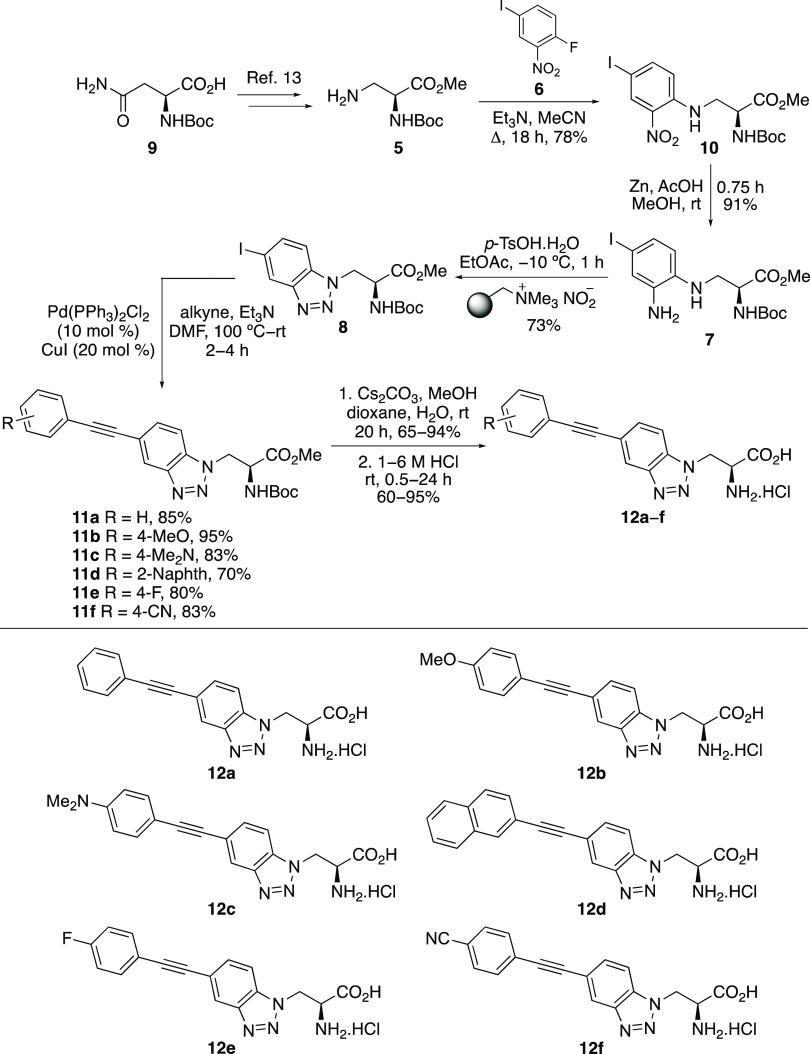
Synthesis of Alkynyl-Fused Benzotriazole-Derived α-Amino Acids **12a**–**f** Isolated yields are shown.

Initially, gram quantities of *N*-Boc-l-3-aminoalanine α-methyl ester **5** were prepared from commercially available *N*-Boc-l-asparagine **9** as previously described by Piantanida and co-workers (Scheme 2).^13^ The four-step route that involved a Hofmann rearrangement and protecting group manipulation gave **5** in 69% overall yield. Nucleophilic aromatic substitution of 3-aminoalanine derivative **5** with 2-fluoro-5-iodonitrobenzene (**6**) using triethylamine gave adduct **10** in 78% yield. Chemoselective nitro group reduction of **10** was then performed using zinc and acetic acid. Reduction under mild acidic conditions and with a short reaction time of 0.75 h allowed full conversion, while maintaining both the C–I bond and Boc-group protection of the amine. This gave aniline **7** in 91% yield. The one-pot activation of the amine as the diazo intermediate and *in situ* cyclization to benzotriazole **8** was performed as previously described by us.^12,14^ Formation of the diazo intermediate was achieved under mild conditions, using a polymer-supported nitrite reagent and *p*-tosic acid.^15,16^ The one-pot reaction was complete after 1 h and gave benzotriazole **8** in 73% yield. Again, the integrity of the Boc-protecting group was not affected by the acidic conditions. The synthesis of the α-amino acids with extended alkynyl-fused benzotriazole chromophores was then achieved using a Sonogashira reaction of iodobenzotriazole **8** with various electron-rich and electron-deficient aryl-substituted acetylenes. Under standard conditions, this gave the coupled products **11a**–**f** in 70–95% yields.^17,18^ Crucial to the success of the coupling reactions was the requirement of an initiation phase at high temperature (100 °C for 0.1 h), before conducting the remainder of the reaction at room temperature. Initial heating allows efficient formation of the active palladium(0) species from the pre-catalyst, while returning to room temperature for the coupling step prevents extensive Glaser–Hay coupling of the terminal alkyne reagent.^19^ Deprotection to the parent α-amino acids was then performed using a two-step approach. Ester hydrolysis with cesium carbonate was followed by the removal of the Boc-group under acidic conditions. Despite the presence of electron-rich alkynes, acidic deprotection proceeded cleanly, without any significant byproducts. Purification by recrystallization gave the amino acid hydrochloride salts **12a**–**f** in good overall yields.

**Scheme 3:**
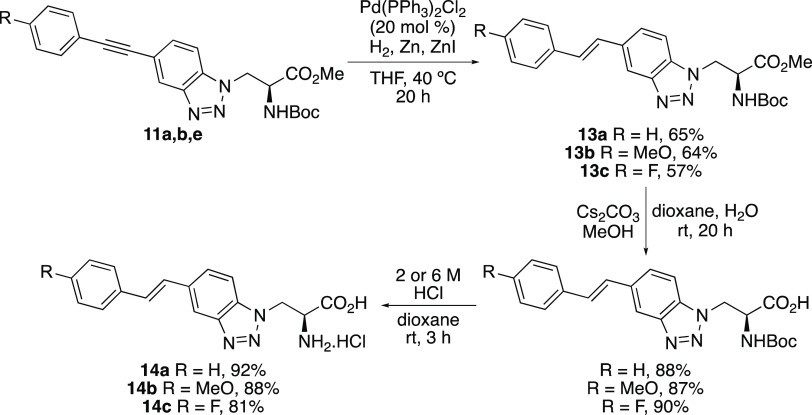
Synthesis of Alkenyl-Fused Benzotriazole-Derived α-Amino Acids **14a**–**c** Isolated yields are shown.

To access the corresponding alkenyl analogues, various approaches were attempted. These included the Heck cross-coupling reaction of styrene with iodobenzotriazole **8** and although successful, competing protodepalladation during the reaction resulted in a low yield of the desired product (32%). A second attempt involved the Suzuki–Miyaura cross-coupling of 2-arylvinylboronic acids with a bromo-analogue of **8**. However, this again gave the alkenyl-coupled benzotriazole in low yield (25%). For these reasons, the reduction of the alkynyl-fused benzotriazoles **11** was considered as a possible approach to the corresponding alkenyl analogues. From the range of literature methods available, a chemo- and stereoselective hydrogenation procedure at atmospheric pressure, reported by Jackowski and co-workers, was considered.^20^ This reaction involves the combination of a palladium catalyst and zinc(II) iodide, which promotes *syn*-hydrogenation, followed by *Z*- to *E*-isomerization. Attempted reduction of alkyne **11b** using the standard conditions (5 mol % of Pd cat. and 25 °C) required a reaction time of 112 h and gave a 2:1 mixture of *E*- and *Z*-alkenes isomers (Table S1). Optimization studies showed that by increasing both the amount of palladium catalyst (20 mol %) and reaction temperature (40 °C), the reaction time for reduction of **11b** could be reduced to 20 h, to give solely *E*-isomer **13b** in 64% yield (Scheme 3). Having prepared an electron-rich analogue, an electron-neutral analogue **11a**, and an electron-deficient analogue **11e** were also subjected to the optimized reduction conditions. In both cases, the reaction generated only the *E*-isomer (**13a** and **13c**) in similar yields. Ester hydrolysis with cesium carbonate and mild acid removal of the Boc-group gave the deprotected amino acids in high overall yields.

**Figure 2:**
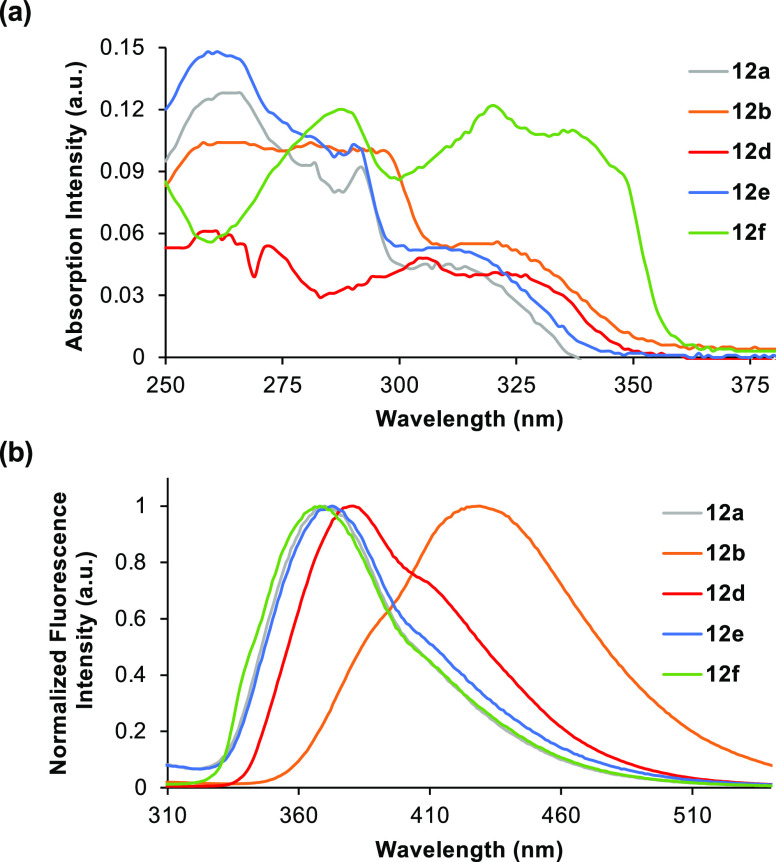
(a) Absorption spectra of **12a**–**b** and **12d**–**f**, recorded at 5 μM in methanol. (b) Emission spectra of **12a**–**b** and **12d**–**f**, recorded at 5 μM in methanol.

**Figure 3:**
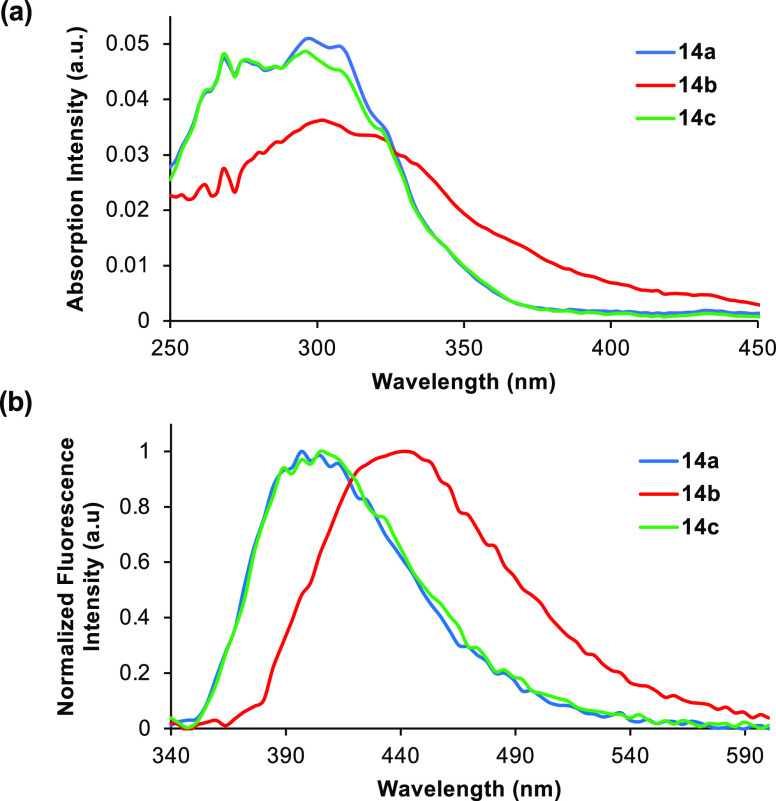
(a) Absorption spectra of **14a**–**c**, recorded at 5 μM in methanol. (b) Emission spectra of **14a**–**c**, recorded at 5 μM in methanol.

Following the synthesis of the alkynyl- and alkenyl-fused benzotriazole-derived α-amino acids, the optical properties were measured for each compound and compared with 5-aryl analogue **4** (Figures 2 and 3, and Table 1).^12,21^ The ultraviolet/visible (UV/visible) absorption and photoluminescence spectra of the α-amino acids were recorded in methanol at a concentration of 5 μM. As proposed, the extended chromophores all displayed red-shifted absorption. Direct comparison of the *p*-methoxy analogues shows absorption bands at 321 nm for alkyne **12b** and 320 nm for alkene **14b***vs* 256 nm for 5-aryl analogue **4**. Strong fluorescence with emission maxima in the visible region was observed for benzotriazoles containing electron-rich aryl side chains.^22^ In addition, the alkynyl series of compounds displayed the most favorable properties. For example, *p*-methoxyphenyl **12b** possessed a quantum yield of 34% and was significantly brighter than both structural analogues **4** and **14b**. These results suggest that the aryl-substituted alkynyl-benzotriazoles can readily adopt a flat conformation which allows effective conjugation across the chromophore, compared to the biaryl (**4**) and alkenyl (**14b**) systems that can relax *via* nonradiative decay pathways due to extra rotational and vibrational modes.^23^ In addition to the strong fluorescence of electron-rich analogues, naphthyl-substituted alkyne **12d** possessed the highest quantum yield (42%) and good brightness. Overall, the strategy of inserting alkynyl and alkenyl spacer units to extend the chromophores has yielded compounds with improved photophysical properties, particularly absorption maxima that no longer overlap with proteinogenic amino acids. It should be noted that while this has resulted in compounds with smaller Stokes shifts than the original series, these are still significantly large (*e.g.*, 7400 cm^−1^ for **12b** and 8677 cm^−1^ for **14b**).

**Table 1:** Photophysical Data of Benzotriazole-Derived α-Amino Acids

amino acid	λ_Abs_ (nm)^a^	ε (cm^–1^ M^–1^)	λ_Em_ (nm)^a^	Stokes shift (cm^–1^)	Φ_F_^b^	brightness (cm^–1^ M^–1^)
**4**	256	23,034	418	15,139	0.17	3857
**12a**	310	25,000	373	5448	0.08	2030
**12b**	321	20,800	421	7400	0.34	7240
**12c**	349	14,500	430	5397	<0.01	10
**12d**	320	12,400	382	5072	0.42	5200
**12e**	310	28,900	371	5304	0.07	2100
**12f**	341	15,000	371	2371	0.17	2470
**14a**	307	18,000	402	7698	0.09	1670
**14b**	320	12,400	443	8677	0.11	1410
**14c**	303	5900	398	7878	0.09	550

a Spectra were recorded at 5 μM in methanol.

b Quantum yields (Φ_F_) were determined in methanol using anthracene and l-tryptophan as standards.

**Figure 4:**
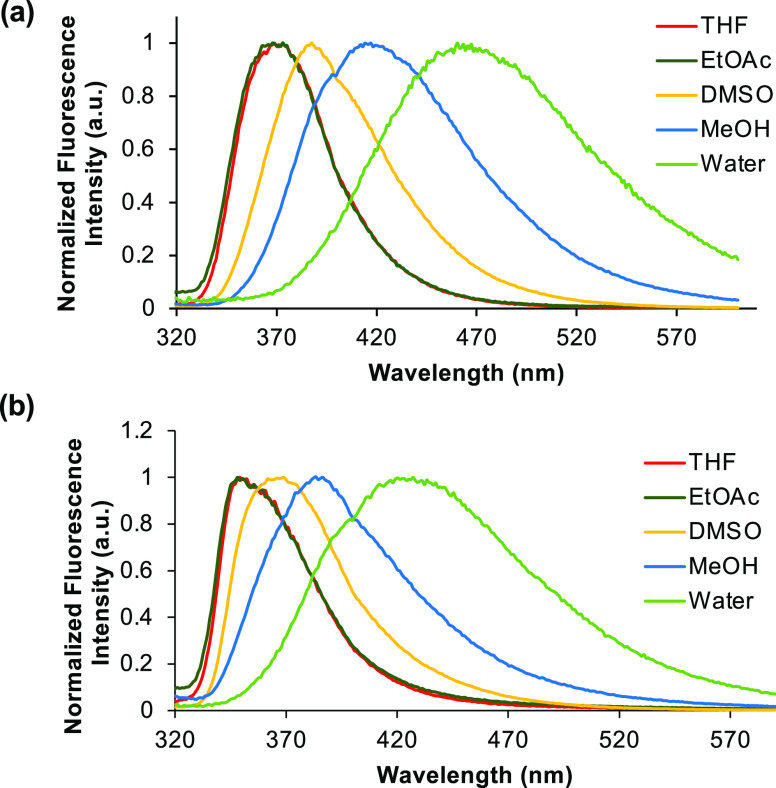
(a) Emission spectra of **12b** in various solvents. (b) Emission spectra of **12d** in various solvents. All spectra were recorded using a concentration of 5 μM.

As the *p*-methoxyphenyl- and naphthyl-substituted alkynyl-fused benzotriazoles **12b** and **12d** were found to be the brightest α-amino acids, the properties of these compounds were further explored *via* a solvatochromic study.^24^ In contrast to the absorption bands of both compounds, which were found to be independent of solvent polarity (see the Supporting Information), the emission maxima displayed significant bathochromic shift with increasing polarity (Figure 4). For example, benzotriazole **12b** displayed an emission maximum at 367 nm in ethyl acetate compared to 460 nm in water. Similarly, benzotriazole **12d** showed a range from 348 to 426 nm. The solvatochromism exhibited by **12b** and **12d** suggests that the excited state has internal charge transfer character, which is stabilized in more polar solvents. The larger bathochromic shift of **12b** implies a stronger dipole across the chromophore, which would be expected for a more electron-rich substituent. The solvatochromism for **12b** and **12d** was further evidenced from Lippert–Mataga plots, in which graphs of Stokes shifts versus solvent orientation polarizability showed a linear correlation (see the SI).^25^ The linearity of these plots confirms the general effect of solvent in the shift of emission bands. Both the bathochromic shift of emission in aqueous solvents and the environment sensitivity of these amino acids suggests potential as probes for chemical biology applications.

## Conclusions

In summary, a series of alkyne-fused benzotriazole-derived α-amino acids have been prepared using nucleophilic aromatic substitution with a 3-aminoalanine derivative, a one-pot diazotization, and cyclization to access the benzotriazole unit and a Sonogashira cross-coupling reaction for the introduction of the unsaturated side chain as the key steps. Access to the corresponding *E*-alkenes was achieved by a chemo- and stereoselective, palladium-catalyzed hydrogenation reaction. Investigation of the photophysical properties of both classes of α-amino acids revealed that extended conjugation resulted in compounds with red-shifted absorption bands. This means these compounds can be excited in the presence of fluorescent proteinogenic α-amino acids. The majority of compounds demonstrated strong fluorescent properties and large Stokes shifts, with the alkyne series possessing the highest quantum yields and brightest chromophores. A solvatochromic study with the brightest α-amino acids showed significant environment sensitivity to solvent polarity. Work is currently underway to investigate the use of compounds **12b** and **12d** as probes in chemical biology applications.

## Experimental Section

The synthesis of compound **5** has been previously described in the literature.^13^ All reagents and starting materials were obtained from commercial sources and used as received. Reactions were performed open to air unless otherwise mentioned. All reactions performed at elevated temperatures were heated using an oil bath. Brine refers to a saturated aqueous solution of sodium chloride. Flash column chromatography was performed using silica gel 60 (40–63 μm). Aluminum-backed plates precoated with silica gel 60F_254_ were used for thin-layer chromatography and were visualized with a UV lamp or by staining with potassium permanganate, vanillin, or ninhydrin. ^1^H NMR spectra were recorded on an NMR spectrometer at either 400 or 500 MHz and data are reported as follows: chemical shift in ppm relative to the solvent as internal standard (CHCl_3_, δ 7.26 ppm; CH_3_OH, δ 3.31 ppm; DMSO, δ 2.50), multiplicity (s = singlet, d = doublet, t = triplet, q = quartet, m = multiplet or overlap of nonequivalent resonances, integration). The abbreviations br s and br d refer to broad singlet and broad doublet, respectively. ^13^C NMR spectra were recorded on an NMR spectrometer at either 101 or 126 MHz and data are reported as follows: chemical shift in ppm relative to tetramethylsilane or the solvent as internal standard (CDCl_3_, δ 77.2 ppm; CD_3_OD, δ 49.0 ppm; DMSO-*d*_6_, δ 39.5), multiplicity with respect to hydrogen (deduced from DEPT experiments, C, CH, CH_2_ or CH_3_). Infrared spectra were recorded on a Fourier transform infrared (FTIR) spectrometer; wavenumbers are indicated in cm^–1^. Mass spectra were recorded using electrospray techniques. High-resolution mass spectra (HRMS) were recorded using quadrupole time-of-flight (Q-TOF) mass spectrometers. Melting points are uncorrected. Optical rotations were determined as solutions irradiating with the sodium D line (λ = 589 nm) using a polarimeter. [α]_D_ values are given in units 10^–1^ deg cm^–1^ g^–1^. UV–vis and fluorescence spectra were recorded on a fluorescence and absorbance spectrometer. Absorbance spectra were recorded with an integration time of 0.05 s and a band pass of 5 nm. Fluorescence spectra were recorded with excitation and emission band pass of 5 nm, an integration time of 2 s, and with detector accumulations set to 1. Quantum yield data were measured using anthracene and l-tryptophan as standard references.

### Methyl (2*S*)-2-[(*tert*-butoxycarbonyl)amino]-3-[(2′-nitro-4′-iodophenyl)amino]propanoate (**10**)

To a solution of methyl (2*S*)-2-[(*tert*-butoxycarbonyl)amino]-3-aminopropanoate (**5**) (1.50 g, 6.99 mmol) in acetonitrile (50 mL) under argon were added 2-fluoro-5-iodonitrobenzene (**6**) (5.60 g, 21.0 mmol) and triethylamine (2.92 mL, 21.0 mmol). The reaction mixture was stirred under reflux for 18 h. After cooling the reaction to ambient temperature, the solvent was removed *in vacuo*. The resulting residue was dissolved in ethyl acetate (50 mL) and washed with water (3 × 50 mL) and brine (50 mL). The organic layer was dried (MgSO_4_), filtered, and concentrated *in vacuo*. Purification by flash column chromatography eluting with 0–10% ethyl acetate in dichloromethane gave methyl (2*S*)-2-[(*tert*-butoxycarbonyl)amino]-3-[(2′-nitro-4′-iodophenyl)amino]propanoate (**10**) as a yellow solid (2.54 g, 78%). Mp 103–105 °C; IR (neat) 3364, 2978, 1744, 1709, 1611, 1500, 1233, 1159 cm^–1^; [α]_D_^21^ +51.5 (*c* 1.0, CHCl_3_); ^1^H NMR (CDCl_3_, 400 MHz): δ 8.46 (d, 1H, *J* = 2.1 Hz), 8.22 (t, 1H, *J* = 5.6 Hz), 7.66 (dd, 1H, *J* = 9.0, 2.1 Hz), 6.80 (d, 1H, *J* = 9.0 Hz), 5.37 (d, 1H, *J* = 6.3 Hz), 4.62–4.54 (m, 1H), 3.81 (s, 3H), 3.79–3.72 (m, 1H), 3.68 (dt, 1H, *J* = 13.2, 5.6 Hz), 1.45 (s, 9H); ^13^C{^1^H} NMR (CDCl_3_, 101 MHz): δ 170.7 (C), 155.2 (C), 144.4 (C), 144.3 (CH), 134.9 (CH), 133.5 (C), 115.9 (CH), 80.7 (C), 75.3 (C), 53.0 (CH and CH_3_), 44.8 (CH_2_), 28.3 (3 × CH_3_); MS (ESI) *m*/*z* 488 (M + Na^+^, 100); HRMS (ESI) *m*/*z*: [M + Na]^+^ calcd for C_15_H_20_IN_3_O_6_Na 488.0289; found 488.0290.

### Methyl (2*S*)-2-[(*tert*-butoxycarbonyl)amino]-3-[(4′-iodo-2′-aminophenyl)amino]propanoate (**7**)

To a solution of methyl (2*S*)-2-[(*tert*-butoxycarbonyl)amino]-3-[(2′-nitro-4′-iodophenyl)amino]propanoate (**10**) (1.80 g, 3.87 mmol) in methanol (40 mL) were added zinc (1.26 g, 19.3 mmol) and acetic acid (1.20 mL, 19.3 mmol). The reaction mixture was stirred for 0.75 h at room temperature, then filtered through Celite, and concentrated *in vacuo*. Purification by flash column chromatography eluting with 10% ethyl acetate in dichloromethane gave methyl (2*S*)-2-[(*tert*-butoxycarbonyl)amino]-3-[(4′-iodo-2′-aminophenyl)amino]propanoate (**7**) as a brown solid (1.53 g, 91%). Mp 176–180 °C; IR (neat) 3348, 2987, 1795, 1697, 1503, 1395, 1250, 1066 cm^–1^; [α]_D_^18^ +24.7 (*c* 0.1, CHCl_3_); ^1^H NMR (CDCl_3_, 400 MHz): δ 7.06 (dd, 1H, *J* = 8.3, 2.0 Hz), 6.99 (d, 1H, *J* = 2.0 Hz), 6.40 (d, 1H, *J* = 8.3 Hz), 5.41 (d, 1H, *J* = 6.7 Hz), 4.62–4.53 (m, 1H), 3.76 (s, 3H), 3.52 (dd, 1H, *J* = 12.5, 4.4 Hz), 3.44–3.31 (m, 3H), 1.44 (s, 9H); ^13^C{^1^H} NMR (CDCl_3_, 101 MHz): δ 171.6 (C), 155.6 (C), 136.6 (C), 136.5 (C), 129.1 (CH), 124.7 (CH), 114.1 (CH), 80.9 (C), 80.5 (C), 53.6 (CH), 52.7 (CH_3_), 46.5 (CH_2_), 28.3 (3 × CH_3_); MS (ESI) *m*/*z* 458 (M + Na^+^, 100); HRMS (ESI) *m*/*z*: [M + Na]^+^ calcd for C_15_H_22_IN_3_O_4_Na 458.0547; found 458.0545.

### Methyl (2*S*)-2-[(*tert*-butoxycarbonyl)amino]-3-(5′-iodo-1*H*-benzo[*d*][1.2.3]triazol-1′-yl)propanoate (**8**)

To a solution of methyl (2*S*)-2-[(*tert*-butoxycarbonyl)amino]-3-[(4′-iodo-2′-aminophenyl)amino]propanoate (**7**) (0.700 g, 1.61 mmol) in ethyl acetate (35 mL) at −10 °C was added *p*-toluenesulfonic acid (0.917 g, 4.82 mmol) and polymer-supported nitrite (1.37 g, containing 4.82 mmol of NO_2_^–^). The reaction mixture was stirred for 1 h, filtered, and the resin washed with ethyl acetate (50 mL). The organic layer was washed with a saturated solution of aqueous sodium hydrogen carbonate (50 mL) and brine (50 mL), dried (MgSO_4_), filtered, and concentrated *in vacuo*. Purification by flash column chromatography eluting with 0–5% ethyl acetate in dichloromethane gave methyl (2*S*)-2-[(*tert*-butoxycarbonyl)amino]-3-(5′-iodo-1*H*-benzo[*d*][1.2.3]triazol-1′-yl)propanoate (**8**) as a gray solid (0.527 g, 73%). Mp 113–116 °C; IR (neat) 3676, 2972, 1701, 1395, 1250, 1163, 1066 cm^–1^; [α]_D_^19^ +21.9 (*c* 0.1, CHCl_3_); ^1^H NMR (CDCl_3_, 400 MHz): δ 8.43 (dd, 1H, *J* = 1.3, 0.6 Hz), 7.73 (dd, 1H, *J* = 8.7, 1.3 Hz), 7.32 (br d, 1H, *J* = 8.7 Hz), 5.31 (d, 1H, *J* = 6.0 Hz), 5.09 (d, 2H, *J* = 4.4 Hz), 4.77 (dt, 1H, *J* = 6.0, 4.4 Hz), 3.78 (s, 3H), 1.42 (s, 9H); ^13^C{^1^H} NMR (CDCl_3_, 101 MHz): δ 169.5 (C), 155.0 (C), 147.5 (C), 136.2 (CH), 133.3 (C), 129.1 (CH), 111.0 (CH), 87.6 (C), 80.8 (C), 53.9 (CH), 53.2 (CH_3_), 48.8 (CH_2_), 28.2 (3 × CH_3_); MS (ESI) *m*/*z* 469 (M + Na^+^, 100); HRMS (ESI) *m*/*z*: [M + Na]^+^ calcd for C_15_H_19_IN_4_O_4_Na 469.0343; found 469.0359.

### Methyl (2*S*)-2-[(*tert*-butoxycarbonyl)amino]-3-[5′-(phenylethynyl)-1*H*-benzo[*d*][1.2.3]triazol-1′-yl]propanoate (**11a**)

To a solution of methyl (2*S*)-2-[(*tert*-butoxycarbonyl)amino]-3-(5′-iodo-1*H*-benzo[*d*][1.2.3]triazol-1′-yl)propanoate (**8**) (0.050 g, 0.11 mmol) in *N,N*′-dimethylformamide (3 mL) were added copper iodide (0.0042 g, 0.022 mmol) and bis(triphenylphosphine)palladium(II) dichloride (0.0077 g, 0.011 mmol). Phenylacetylene (0.015 mL, 0.14 mmol) was dissolved in degassed triethylamine (7 mL) and added to the reaction mixture. The solution was heated to 100 °C for 0.1 h and stirred at room temperature for 2 h. The solution was concentrated *in vacuo*, dissolved in ethyl acetate (20 mL), washed with water (5 × 10 mL) and brine (2 × 10 mL), dried (MgSO_4_), and concentrated *in vacuo*. Purification by flash column chromatography eluting with 0–5% ethyl acetate in dichloromethane gave methyl (2*S*)-2-[(*tert*-butoxycarbonyl)amino]-3-[5′-(phenylethynyl)-1*H*-benzo[*d*][1.2.3]triazol-1′-yl]propanoate (**11a**) as a yellow oil (0.039 g, 85%). IR (neat) 3357, 2978, 2360, 1745, 1708, 1498, 1162, 756 cm^–1^; [α]_D_^19^ +21.1 (*c* 0.1, CHCl_3_); ^1^H NMR (CDCl_3_, 400 MHz): δ 8.43 (br s, 1H), 7.63 (d, 1H, *J* = 8.6 Hz), 7.57 (dd, 2H, *J* = 6.5, 3.2 Hz), 7.50 (d, 1H, *J* = 8.6 Hz), 7.39–7.34 (m, 3H), 5.39 (d, 1H, *J* = 6.6 Hz), 5.12 (d, 2H, *J* = 4.5 Hz), 4.79 (dt, 1H, *J* = 6.6, 4.5 Hz), 3.78 (s, 3H), 1.43 (s, 9H); ^13^C{^1^H} NMR (CDCl_3_, 101 MHz): δ 169.5 (C), 155.0 (C), 145.6 (C), 133.5 (C), 131.7 (2 × CH), 131.2 (CH), 128.5 (CH), 128.4 (2 × CH), 123.3 (CH), 122.9 (C), 119.4 (C), 109.5 (CH), 89.5 (C), 88.7 (C), 80.7 (C), 53.9 (CH), 53.2 (CH_3_), 48.7 (CH_2_), 28.2 (3 × CH_3_); MS (ESI) *m*/*z* 443 (M + Na^+^, 100); HRMS (ESI) *m*/*z*: [M + Na]^+^ calcd for C_23_H_24_N_4_O_4_Na 443.1690; found 443.1687.

### Methyl (2*S*)-2-[(*tert*-butoxycarbonyl)amino]-3-{5′-[(4‴-methoxyphenyl)ethynyl]-1*H*-benzo[*d*][1.2.3]triazol-1′-yl}propanoate (**11b**)

Methyl (2*S*)-2-[(*tert*-butoxycarbonyl)amino]-3-{5′-[(4‴-methoxyphenyl)ethynyl]-1*H*-benzo[*d*][1.2.3]triazol-1′-yl}propanoate (**11b**) was synthesized as described for **11a** using methyl (2*S*)-2-[(*tert*-butoxycarbonyl)amino]-3-(5′-iodo-1*H*-benzo[*d*][1.2.3]triazol-1′-yl)propanoate (**8**) (0.100 g, 0.220 mmol), *N,N*′-dimethylformamide (7 mL), copper iodide (0.00840 g, 0.0440 mmol), bis(triphenylphosphine)palladium(II) dichloride (0.0154 g, 0.0220 mmol), 4-methoxyphenylacetylene (0.0400 g, 0.280 mmol), and triethylamine (14 mL). Purification by flash column chromatography eluting with 5% ethyl acetate in dichloromethane gave methyl (2*S*)-2-[(*tert*-butoxycarbonyl)amino]-3-{5′-[(4‴-methoxyphenyl)ethynyl]-1*H*-benzo[*d*][1.2.3]triazol-1′-yl}propanoate (**11b**) as a yellow solid (0.0950 g, 95%). Mp 155–158 °C; IR (neat) 3369, 2976, 2357, 1748, 1714, 1605, 1514, 1250, 1171, 1033, 833 cm^–1^; [α]_D_^18^ +17.3 (*c* 0.1, CHCl_3_); ^1^H NMR (CDCl_3_, 400 MHz): δ 8.19 (br s, 1H), 7.61 (d, 1H, *J* = 8.6 Hz), 7.53–7.47 (m, 3H), 6.90 (d, 2H, *J* = 8.8 Hz), 5.32 (d, 1H, *J* = 6.5 Hz), 5.11 (d, 2H, *J* = 4.3 Hz), 4.79 (dt, 1H, *J* = 6.5, 4.3 Hz), 3.84 (s, 3H), 3.78 (s, 3H), 1.43 (s, 9H); ^13^C{^1^H} NMR (CDCl_3_, 101 MHz): δ 169.6 (C), 159.8 (C), 155.0 (C), 145.7 (C), 133.4 (C), 133.1 (2 × CH), 131.2 (CH), 123.0 (CH), 119.8 (C), 115.0 (C), 114.1 (2 × CH), 109.4 (CH), 89.6 (C), 87.4 (C), 80.7 (C), 55.3 (CH_3_), 53.9 (CH), 53.2 (CH_3_), 48.7 (CH_2_), 28.3 (3 × CH_3_); MS (ESI) *m*/*z* 473 (M + Na^+^, 100); HRMS (ESI) *m*/*z*: [M + Na]^+^ calcd for C_24_H_26_N_4_O_5_Na 473.1795; found 473.1792.

### Methyl (2*S*)-2-[(*tert*-butoxycarbonyl)amino]-3-{5′-[(4‴-dimethylaminophenyl)ethynyl]-1*H*-benzo[*d*][1.2.3]triazol-1′-yl}propanoate (**11c**)

Methyl (2*S*)-2-[(*tert*-butoxycarbonyl)amino]-3-{5′-[(4‴-dimethylaminophenyl)ethynyl]-1*H*-benzo[*d*][1.2.3]triazol-1′-yl}propanoate (**11c**) was synthesized as described for **11a** using methyl (2*S*)-2-[(*tert*-butoxycarbonyl)amino]-3-(5′-iodo-1*H*-benzo[*d*][1.2.3]triazol-1′-yl)propanoate (**8**) (0.400 g, 0.900 mmol), *N,N*′-dimethylformamide (18 mL), copper iodide (0.0340 g, 0.180 mmol), bis(triphenylphosphine)palladium(II) dichloride (0.0630 g, 0.0900 mmol), 4-dimethylaminophenylacetylene (0.170 g, 1.17 mmol), and triethylamine (42 mL). Purification by flash column chromatography eluting with 80% diethyl ether in hexane gave methyl (2*S*)-2-[(*tert*-butoxycarbonyl)amino]-3-{5′-[(4‴-dimethylaminophenyl)ethynyl]-1*H*-benzo[*d*][1.2.3]triazol-1′-yl}propanoate (**11c**) as a yellow oil (0.346 g, 83%). IR (neat) 3318, 2927, 2207, 1746, 1709, 1606, 1510, 1365, 1164, 757 cm^–1^; [α]_D_^17^ +12.9 (*c* 0.2, CHCl_3_); ^1^H NMR (CDCl_3_, 400 MHz): δ 8.17 (br s, 1H), 7.60 (dd, 1H, *J* = 8.6, 1.1 Hz), 7.53–7.47 (m, 3H), 6.90 (d, 2H, *J* = 8.8 Hz), 5.33 (d, 1H, *J* = 6.8 Hz), 5.10 (d, 2H, *J* = 4.3 Hz), 4.79 (dt, 1H, *J* = 6.8, 4.3 Hz), 3.77 (s, 3H), 3.00 (s, 6H), 1.43 (s, 9H); ^13^C{^1^H} NMR (CDCl_3_, 101 MHz): δ 169.6 (C), 155.0 (C), 150.3 (C), 145.8 (C), 133.1 (C), 132.8 (2 × CH), 131.3 (CH), 122.5 (CH), 120.4 (C), 111.9 (2 × CH), 109.6 (C), 109.3 (CH), 90.9 (C), 86.7 (C), 80.7 (C), 53.9 (CH), 53.1 (CH_3_), 48.7 (CH_2_), 40.2 (2 × CH_3_), 28.3 (3 × CH_3_); MS (ESI) *m*/*z* 486 (M + Na^+^, 100); HRMS (ESI) *m*/*z*: [M + Na]^+^ calcd for C_25_H_29_N_5_O_4_Na 486.2112; found 486.2114.

### Methyl (2*S*)-2-[(*tert*-butoxycarbonyl)amino]-3-{5′-[(napthalen-2‴-yl)ethynyl]-1*H*-benzo[*d*][1.2.3]triazol-1′-yl}propanoate (**11d**)

Methyl (2*S*)-2-[(*tert*-butoxycarbonyl)amino]-3-{5′-[(napthalen-2‴-yl)ethynyl]-1*H*-benzo[*d*][1.2.3]triazol-1′-yl}propanoate (**11d**) was synthesized as described for **11a** using methyl (2*S*)-2-[(*tert*-butoxycarbonyl)amino]-3-(5′-iodo-1*H*-benzo[*d*][1.2.3]triazol-1′-yl)propanoate (**8**) (0.150 g, 0.340 mmol), *N,N*′-dimethylformamide (9 mL), copper iodide (0.0128 g, 0.0670 mmol), bis(triphenylphosphine)palladium(II) dichloride (0.0239 g, 0.0340 mmol), 2-ethynylnapthalene (0.0670 g, 0.440 mmol), and triethylamine (21 mL). The reaction mixture was stirred at room temperature for 4 h. Purification by flash column chromatography eluting with 40% ethyl acetate in hexane gave methyl (2*S*)-2-[(*tert*-butoxycarbonyl)amino]-3-{5′-[(napthalen-2‴-yl)ethynyl]-1*H*-benzo[*d*][1.2.3]triazol-1′-yl}propanoate (**11d**) as a yellow solid (0.112 g, 70%). Mp 180–182 °C; IR (neat) 2980, 2360, 1746, 1708, 1503, 1366, 1163, 753 cm^–1^; [α]_D_^21^ +5.5 (*c* 0.2, CHCl_3_); ^1^H NMR (CDCl_3_, 500 MHz): δ 8.24 (br s, 1H), 8.08 (br s, 1H), 7.84–7.79 (m, 3H), 7.67 (dd, 1H, *J* = 8.5, 0.8 Hz), 7.60 (dd, 1H, *J* = 8.5, 1.5 Hz), 7.53–7.47 (m, 3H), 5.45 (d, 1H, *J* = 6.5 Hz), 5.11 (d, 2H, *J* = 4.5 Hz), 4.79 (dt, 1H, *J* = 6.5, 4.5 Hz), 3.78 (s, 3H), 1.44 (s, 9H); ^13^C{^1^H} NMR (CDCl_3_, 126 MHz): δ 169.6 (C), 155.1 (C), 145.6 (C), 133.6 (C), 133.0 (C), 132.9 (C), 131.6 (CH), 128.3 (CH), 128.1 (CH), 127.8 (3 × CH), 126.8 (CH), 126.7 (CH), 123.3 (CH), 120.2 (C), 119.5 (C), 109.6 (CH), 90.0 (C), 89.1 (C), 80.7 (C), 54.0 (CH), 53.2 (CH_3_), 48.7 (CH_2_), 28.3 (3 × CH_3_); MS (ESI) *m*/*z* 493 (M + Na^+^, 100); HRMS (ESI) *m*/*z*: [M + Na]^+^ calcd for C_27_H_26_N_4_O_4_Na 493.1846; found 493.1844.

### Methyl (2*S*)-2-[(*tert*-butoxycarbonyl)amino]-3-{5′-[(4‴-fluorophenyl)ethynyl]-1*H*-benzo[*d*][1.2.3]triazol-1′-yl}propanoate (**11e**)

Methyl (2*S*)-2-[(*tert*-butoxycarbonyl)amino]-3-{5′-[(4‴-fluorophenyl)ethynyl]-1*H*-benzo[*d*][1.2.3]triazol-1′-yl}propanoate (**11e**) was synthesized as described for **11a** using methyl (2*S*)-2-[(*tert*-butoxycarbonyl)amino]-3-(5′-iodo-1*H*-benzo[*d*][1.2.3]triazol-1′-yl)propanoate (**8**) (0.100 g, 0.220 mmol), *N,N*′-dimethylformamide (6 mL), copper iodide (0.00840 g, 0.0440 mmol), bis(triphenylphosphine)palladium(II) dichloride (0.0154 g, 0.0220 mmol), 4-fluorophenylacetylene (0.0336 g, 0.280 mmol), and triethylamine (14 mL). Purification by flash column chromatography eluting with 0–5% ethyl acetate in dichloromethane gave methyl (2*S*)-2-[(*tert*-butoxycarbonyl)amino]-3-{5′-[(4‴-fluorophenyl)ethynyl]-1*H*-benzo[*d*][1.2.3]triazol-1′-yl}propanoate (**11e**) as an off-white solid (0.0770 g, 80%). Mp 145–147 °C; IR (neat) 3358, 2976, 2359, 1748, 1709, 1510, 1221, 1157, 835 cm^–1^; [α]_D_^18^ +21.4 (*c* 0.1, CHCl_3_); ^1^H NMR (CDCl_3_, 400 MHz): δ 8.21 (br s, 1H), 7.61 (dd, 1H, *J* = 8.6, 1.1 Hz), 7.51–7.38 (m, 3H), 7.07 (t, 2H, *J* = 8.7 Hz), 5.33 (d, 1H, *J* = 6.6 Hz), 5.12 (d, 2H, *J* = 4.4 Hz), 4.79 (dt, 1H, *J* = 6.6, 4.4 Hz), 3.78 (s, 3H), 1.43 (s, 9H); ^13^C{^1^H} NMR (CDCl_3_, 101 MHz): δ 169.5 (C), 162.7 (d, ^1^*J*_C–F_ 250.0 Hz, C), 155.0 (C), 145.6 (C), 133.6 (d, ^3^*J*_C–F_ 8.4 Hz, 2 × CH), 133.5 (C), 131.1 (CH), 123.3 (CH), 119.2 (C), 119.1 (d, ^4^*J*_C–F_ 3.6 Hz, C), 115.8 (d, ^2^*J*_C–F_ 22.1 Hz, 2 × CH), 109.6 (CH), 88.5 (C), 88.4 (d, ^5^*J*_C–F_ 1.2 Hz, C), 80.8 (C), 53.9 (CH), 53.2 (CH_3_), 48.7 (CH_2_), 28.2 (3 × CH_3_); MS (ESI) *m*/*z* 461 (M + Na^+^, 100); HRMS (ESI) *m*/*z*: [M + Na]^+^ calcd for C_23_H_23_FN_4_O_4_Na 461.1596; found 461.1592.

### Methyl (2*S*)-2-[(*tert*-butoxycarbonyl)amino]-3-{5′-[(4‴-cyanophenyl)ethynyl]-1*H*-benzo[*d*][1.2.3]triazol-1′-yl}propanoate (**11f**)

Methyl (2*S*)-2-[(*tert*-butoxycarbonyl)amino]-3-{5′-[(4‴-cyanophenyl)ethynyl]-1*H*-benzo[*d*][1.2.3]triazol-1′-yl}propanoate (**11f**) was synthesized as described for **11a** using methyl (2*S*)-2-[(*tert*-butoxycarbonyl)amino]-3-(5′-iodo-1*H*-benzo[*d*][1.2.3]triazol-1′-yl)propanoate (**8**) (0.150 g, 0.340 mmol), *N,N*′-dimethylformamide (9 mL), copper iodide (0.0130 g, 0.0680 mmol), bis(triphenylphosphine)palladium(II) dichloride (0.0239 g, 0.0340 mmol), 4-cyanophenylacetylene (0.0520 mL, 0.442 mmol), and triethylamine (21 mL). Purification by flash column chromatography eluting with 5% ethyl acetate in dichloromethane gave methyl (2*S*)-2-[(*tert*-butoxycarbonyl)amino]-3-{5′-[(4‴-cyanophenyl)ethynyl]-1*H*-benzo[*d*][1.2.3]triazol-1′-yl}propanoate (**11f**) as a yellow solid (0.125 g, 83%). Mp 110–115 °C; IR (neat) 3361, 2984, 2230, 1747, 1690, 1519, 1252, 1155, 1109, 837 cm^–1^; [α]_D_^22^ +6.1 (*c* 0.1, CHCl_3_); ^1^H NMR (CDCl_3_, 500 MHz): δ 8.26 (s, 1H), 7.69–7.62 (m, 5H), 7.54 (d, 1H, *J* = 8.6 Hz), 5.33 (d, 1H, *J* = 6.4 Hz), 5.13 (d, 2H, *J* = 4.5 Hz), 4.79 (dt, 1H, *J* = 6.4, 4.5 Hz), 3.80 (s, 3H), 1.43 (s, 9H); ^13^C{^1^H} NMR (CDCl_3_, 126 MHz): δ 169.6 (C), 155.1 (C), 145.6 (C), 134.1 (C), 132.3 (4 × CH), 131.2 (CH), 128.0 (C), 124.1 (CH), 118.6 (C), 118.4 (C), 111.9 (C), 109.9 (CH), 93.2 (C), 88.0 (C), 80.9 (C), 54.0 (CH), 53.4 (CH_3_), 48.9 (CH_2_), 28.4 (3 × CH_3_); MS (ESI) *m*/*z* 446 (M + H^+^, 100); HRMS (ESI) *m*/*z*: [M + H]^+^ calcd for C_24_H_23_N_5_O_4_H 446.1823; found 446.1832.

### (2*S*)-2-Amino-3-[5′-(phenylethynyl)-1*H*-benzo[*d*][1.2.3]triazol-1′-yl]propanoic Acid Hydrochloride (**12a**)

To a solution of methyl (2*S*)-2-[(*tert*-butoxycarbonyl)amino]-3-[5′-(phenylethynyl)-1*H*-benzo[*d*][1.2.3]triazol-1′-yl]propanoate (**11a**) (0.100 g, 0.240 mmol) in a mixture of methanol (6 mL) and 1,4-dioxane (6 mL) was added a solution of cesium carbonate (0.101 g, 0.310 mmol) in water (3 mL). The reaction mixture was stirred at room temperature for 20 h and then concentrated *in vacuo*. The resulting residue was dissolved in water (50 mL) and acidified to pH 1 with 2 M aqueous hydrochloric acid. The aqueous layer was extracted with dichloromethane (3 × 30 mL), and the combined organic layers were washed with water (30 mL), dried (MgSO_4_), and concentrated *in vacuo* to give (2*S*)-2-[(*tert*-butoxycarbonyl)amino]-3-[5′-(phenylethynyl)-1*H*-benzo[*d*][1.2.3]triazol-1′-yl]propanoic acid as a yellow solid (0.0630 g, 65%). This was used for the next reaction without any further purification. To a solution of (2*S*)-2-[(*tert*-butoxycarbonyl)amino]-3-[5′-(phenylethynyl)-1*H*-benzo[*d*][1.2.3]triazol-1′-yl]propanoic acid (0.0500 g, 0.120 mmol) in acetonitrile (0.1 mL) was added 2 M aqueous hydrochloric acid (3 mL). The reaction mixture was stirred at room temperature for 6 h and then concentrated *in vacuo*. Trituration with chloroform gave (2*S*)-2-amino-3-[5′-(phenylethynyl)-1*H*-benzo[*d*][1.2.3]triazol-1′-yl]propanoic acid hydrochloride (**12a**) as an off-white solid (0.0340 g, 83%). Mp 245–247 °C (decomposition); IR (neat) 2920, 2359, 1732, 1472, 1338, 686 cm^–1^; [α]_D_^18^ +4.9 (*c* 0.1, MeOH); ^1^H NMR (CD_3_OD, 400 MHz): δ 8.22 (br s, 1H), 7.85 (dd, 1H, *J* = 8.7, 0.6 Hz), 7.75 (dd, 1H, *J* = 8.7, 1.3 Hz), 7.61–7.51 (m, 2H), 7.45–7.35 (m, 3H), 5.37 (dd, 1H, *J* = 15.5, 5.8 Hz), 5.27 (dd, 1H, *J* = 15.5, 4.1 Hz), 4.77 (dd, 1H, *J* = 5.8, 4.1 Hz); ^13^C{^1^H} NMR (CD_3_OD, 101 MHz): δ 167.6 (C), 145.4 (C), 133.2 (C), 131.3 (CH), 131.2 (2 × CH), 128.4 (CH), 128.3 (2 × CH), 122.7 (C), 122.0 (CH), 120.0 (C), 110.3 (CH), 89.3 (C), 87.8 (C), 52.2 (CH), 46.8 (CH_2_); MS (ESI) *m*/*z* 307 (M + H^+^, 100); HRMS (ESI) *m*/*z*: [M + H]^+^ calcd for C_17_H_14_N_4_O_2_H 307.1190; found 307.1191.

### (2*S*)-2-Amino-3-{5′-[(4‴-methoxyphenyl)ethynyl]-1*H*-benzo[*d*][1.2.3]triazol-1′-yl}propanoic Acid Hydrochloride (**12b**)

(2*S*)-2-Amino-3-{5′-[(4‴-methoxyphenyl)ethynyl]-1*H*-benzo[*d*][1.2.3]triazol-1′-yl}propanoic acid hydrochloride (**12b**) was prepared as described for **12a** using methyl (2*S*)-2-[(*tert*-butoxycarbonyl)amino]-3-{5′-[(4‴-methoxyphenyl)ethynyl]-1*H*-benzo[*d*][1.2.3]triazol-1′-yl}propanoate (**11b**) (0.0800 g, 0.180 mmol) and cesium carbonate (0.0750 g, 0.230 mmol). This gave (2*S*)-2-[(*tert*-butoxycarbonyl)amino]-3-{5′-[(4‴-methoxyphenyl)ethynyl]-1*H*-benzo[*d*][1.2.3]triazol-1′-yl}propanoic acid (0.0710 g, 91%) as a yellow solid. This was used for the next reaction without any further purification. To a solution of (2*S*)-2-[(*tert*-butoxycarbonyl)amino]-3-{5′-[(4‴-methoxyphenyl)ethynyl]-1*H*-benzo[*d*][1.2.3]triazol-1′-yl}propanoic acid (0.0500 g, 0.110 mmol) in dioxane (2 mL) was added 2 M aqueous hydrochloric acid (0.8 mL). The reaction mixture was stirred at room temperature for 24 h and concentrated *in vacuo*. Purification by recrystallization from methanol and chloroform gave (2*S*)-2-amino-3-{5′-[(4‴-methoxyphenyl)ethynyl]-1*H*-benzo[*d*][1.2.3]triazol-1′-yl}propanoic acid hydrochloride (**12b**) as an off-white solid (0.0340 g, 83%). Mp 274–276 °C (decomposition); IR (neat) 3395, 2932, 2214, 1728, 1605, 1512, 1250, 1173, 1026, 826 cm^–1^; [α]_D_^17^ +6.0 (*c* 0.1, MeOH); ^1^H NMR (DMSO-*d*_6_, 400 MHz): δ 8.13 (dd, 1H, *J* = 1.2, 0.7 Hz), 7.96 (dd, 1H, *J* = 8.6, 0.7 Hz), 7.72 (dd, 1H, *J* = 8.6, 1.2 Hz), 7.54 (d, 2H, *J* = 8.9 Hz), 7.01 (d, 2H, *J* = 8.9 Hz), 5.22 (dd, 1H, *J* = 15.0, 5.0 Hz), 5.17 (dd, 1H, *J* = 15.0, 5.0 Hz), 4.58 (t, 1H, *J* = 5.0 Hz), 3.81 (s, 3H); ^13^C{^1^H} NMR (DMSO-*d*_6_, 126 MHz): δ 168.3 (C), 159.7 (C), 145.2 (C), 133.3 (C), 133.1 (2 × CH), 130.7 (CH), 121.9 (CH), 118.7 (C), 114.5 (C), 114.0 (2 × CH), 110.5 (CH), 89.4 (C), 87.8 (C), 55.3 (CH_3_), 52.0 (CH), 47.2 (CH_2_); MS (ESI) *m*/*z* 359 (M + Na^+^, 100); HRMS (ESI) *m*/*z*: [M + Na]^+^ calcd for C_18_H_16_N_4_O_3_Na 359.1115; found 359.1105.

### (2*S*)-2-Amino-3-{5′-[(4‴-dimethylaminophenyl)ethynyl]-1*H*-benzo[*d*][1.2.3]triazol-1′-yl}propanoic Acid Hydrochloride (**12c**)

(2*S*)-2-Amino-3-{5′-[(4‴-dimethylaminophenyl)ethynyl]-1*H*-benzo[*d*][1.2.3]triazol-1′-yl}propanoic acid hydrochloride (**12c**) was prepared as for **12a** using methyl (2*S*)-2-[(*tert*-butoxycarbonyl)amino]-3-{5′-[(4‴-dimethylaminophenyl)ethynyl]-1*H*-benzo[*d*][1.2.3]triazol-1′-yl}propanoate (**11c**) (0.100 g, 0.220 mmol) and cesium carbonate (0.0910 g, 0.280 mmol). This gave (2*S*)-2-[(*tert*-butoxycarbonyl)amino]-3-{5′-[(4‴-dimethylaminophenyl)ethynyl]-1*H*-benzo[*d*][1.2.3]triazol-1′-yl}propanoic acid (0.0450 g, 66%) as an off-white solid. This was used for the next step without further purification. To a solution of (2*S*)-2-[(*tert*-butoxycarbonyl)amino]-3-{5′-[(4‴-dimethlaminophenyl)ethynyl]-1*H*-benzo[*d*][1.2.3]triazol-1′-yl}propanoic acid (0.0400 g, 0.0890 mmol) in acetonitrile (0.05 mL) was added 1 M aqueous hydrochloric acid (1 mL). The reaction mixture was stirred at room temperature for 0.5 h and concentrated *in vacuo*. Purification by trituration with chloroform gave (2*S*)-2-amino-3-{5′-[(4‴-dimethylaminophenyl)ethynyl]-1*H*-benzo[*d*][1.2.3]triazol-1′-yl}propanoic acid hydrochloride (**12c**) as a yellow oil (0.0300 g, 88%); IR (neat) 3406, 2920, 2361, 1740, 1694, 1508, 1211, 1130, 841 cm^–1^; [α]_D_^19^–4.5 (*c* 0.1, MeOH); ^1^H NMR (CD_3_OD, 400 MHz): δ 8.24 (br s, 1H), 7.89 (dd, 1H, *J* = 8.7, 0.7 Hz), 7.77 (dd, 1H, *J* = 8.7, 1.3 Hz), 7.74 (d, 2H, *J* = 8.9 Hz), 7.59 (d, 2H, *J* = 8.9 Hz), 5.40 (dd, 1H, *J* = 15.5, 5.4 Hz), 5.29 (dd, 1H, *J* = 15.5, 4.4 Hz), 4.78 (dd, 1H, *J* = 5.4, 4.4 Hz), 3.27 (s, 6H); ^13^C{^1^H} NMR (CD_3_OD, 101 MHz): δ 167.8 (C), 145.2 (C), 143.6 (C), 133.2 (2 × CH), 131.4 (CH), 130.9 (C), 122.3 (CH), 119.6 (2 × CH), 111.5 (C), 110.6 (CH), 109.7 (C), 89.5 (C), 87.8 (C), 52.2 (CH), 46.8 (CH_2_), 44.9 (2 × CH_3_); MS (ESI) *m*/*z* 350 (M + H^+^, 100); HRMS (ESI) *m*/*z*: [M + H]^+^ calcd for C_19_H_19_N_5_O_2_H 350.1612; found 350.1616.

### (2*S*)-2-Amino-3-{5′-[(napthalen-2″-yl)ethynyl]-1*H*-benzo[*d*][1.2.3]triazol-1′-yl}propanoic Acid Hydrochloride (**12d**)

(2*S*)-2-Amino-3-{5′-[(napthalen-2″-yl)ethynyl]-1*H*-benzo[*d*][1.2.3]triazol-1′-yl}propanoic acid hydrochloride (**12d**) was prepared as described for **12a** using methyl (2*S*)-2-[(*tert*-butoxycarbonyl)amino]-3-{5′-[(napthalen-2″-yl)ethynyl]-1*H*-benzo[*d*][1.2.3]triazol-1′-yl}propanoate (**11d**) (0.100 g, 0.210 mmol) and cesium carbonate (0.0890 g, 0.270 mmol). This gave (2*S*)-2-[(*tert*-butoxycarbonyl)amino]-3-{5′-[(napthalen-2″-yl)ethynyl]-1*H*-benzo[*d*][1.2.3]triazol-1′-yl}propanoic acid (0.0850 g, 91%) as a yellow solid. This was used for the next reaction without any further purification. To a solution of (2*S*)-2-[(*tert*-butoxycarbonyl)amino]-3-{5′-[(napthalen-2″-yl)ethynyl]-1*H*-benzo[*d*][1.2.3]triazol-1′-yl}propanoic acid (0.0500 g, 0.109 mmol) in 1,4-dioxane (0.1 mL) was added 2 M aqueous hydrochloric acid (1 mL). The reaction mixture was stirred at room temperature for 3 h and concentrated *in vacuo*. Purification by recrystallization from methanol and diethyl ether gave (2*S*)-2-amino-3-{5′-[(napthalen-2″-yl)ethynyl]-1*H*-benzo[*d*][1.2.3]triazol-1′-yl}propanoic acid hydrochloride (**12d**) as a yellow solid (0.0200 g, 80%). Mp 195–198 °C; IR (neat) 3321, 2849, 2353, 1742, 1487, 1235, 1057, 810 cm^–1^; [α]_D_^23^ +14.7 (*c* 0.1, MeOH); ^1^H NMR (CD_3_OD, 400 MHz): δ 8.25 (s, 1H), 8.10 (br s, 1H), 7.92–7.84 (m, 4H), 7.79 (d, 1H, *J* = 8.4 Hz), 7.60 (dd, 1H, *J* = 8.5, 1.4 Hz), 7.56–7.50 (m, 2H), 5.38 (dd, 1H, *J* = 15.3, 4.9 Hz), 5.28 (dd, 1H, *J* = 15.3, 3.0 Hz), 4.81 (br s, 1H); ^13^C{^1^H} NMR (CD_3_OD, 101 MHz): δ 167.6 (C), 145.5 (C), 133.1 (2 × C), 131.3 (CH), 131.2 (CH), 127.9 (CH), 127.8 (CH), 127.5 (CH and C), 127.4 (CH), 126.7 (CH), 126.5 (CH), 122.1 (CH), 120.0 (2 × C), 110.4 (CH), 89.7 (C), 88.1 (C), 52.2 (CH), 46.8 (CH_2_); MS (ESI) *m*/*z* 357 (M + H^+^, 100); HRMS (ESI) *m*/*z*: [M + H]^+^ Calcd for C_21_H_16_N_4_O_2_H 357.1346; Found 357.1350.

### (2*S*)-2-Amino-3-{5′-[(4‴-fluorophenyl)ethynyl]-1*H*-benzo[*d*][1.2.3]triazol-1′-yl}propanoic Acid Hydrochloride (**12e**)

(2*S*)-2-Amino-3-{5′-[(4‴-fluorophenyl)ethynyl]-1*H*-benzo[*d*][1.2.3]triazol-1′-yl}propanoic acid hydrochloride (**12e**) was prepared as described for **12a** using methyl (2*S*)-2-[(*tert*-butoxycarbonyl)amino]-3-{5′-[(4‴-fluorophenyl)ethynyl]-1*H*-benzo[*d*][1.2.3]triazol-1′-yl}propanoate (**11e**) (0.0800 g, 0.180 mmol) and cesium carbonate (0.0750 g, 0.230 mmol). This gave (2*S*)-2-[(*tert*-butoxycarbonyl)amino]-3-{5′-[(4‴-fluorophenyl)ethynyl]-1*H*-benzo[*d*][1.2.3]triazol-1′-yl}propanoic acid (0.0610 g, 79%) as an off-white solid. This was used for the next step without further purification. To a solution of (2*S*)-2-[(*tert*-butoxycarbonyl)amino]-3-{5′-[(4‴-fluorophenyl)ethynyl]-1*H*-benzo[*d*][1.2.3]triazol-1′-yl}propanoic acid (0.0500 g, 0.120 mmol) in acetonitrile (3 mL) was added 6 M aqueous hydrochloric acid (2 mL). The reaction mixture was stirred at room temperature for 3 h and concentrated *in vacuo*. Purification by trituration with chloroform gave (2*S*)-2-amino-3-{5′-[(4‴-fluorophenyl)ethynyl]-1*H*-benzo[*d*][1.2.3]triazol-1′-yl}propanoic acid hydrochloride (**12e**) as a white solid (0.0270 g, 64%). Mp 280–285 °C (decomposition); IR (neat) 2921, 2359, 1742, 1508, 1219, 833 cm^–1^; [α]_D_^17^ +13.6 (*c* 0.1, MeOH); ^1^H NMR (CD_3_OD, 400 MHz): δ 8.19 (s, 1H), 7.85 (d, 1H, *J* = 8.6 Hz), 7.73 (d, 1H, *J* = 8.6 Hz), 7.59 (dd, 2H, *J* = 8.5, 5.5 Hz), 7.14 (t, 2H, *J* = 8.5 Hz), 5.36 (dd, 1H, *J* = 15.4, 5.5 Hz), 5.27 (dd, 1H, *J* = 15.4, 3.8 Hz), 4.79–4.72 (m, 1H); ^13^C{^1^H} NMR (CD_3_OD, 101 MHz): δ 167.5 (C), 162.8 (d, ^1^*J*_C–F_ 247.8 Hz, C), 145.4 (C), 133.4 (d, ^3^*J*_C–F_ 8.5 Hz, 2 × CH), 133.2 (C), 131.2 (CH), 122.0 (CH), 119.9 (C), 119.0 (d, ^4^*J*_C–F_ 3.5 Hz, C), 115.4 (d, ^2^*J*_C–F_ 22.5 Hz, 2 × CH), 110.3 (CH), 88.2 (C), 87.5 (C), 52.2 (CH), 46.8 (CH_2_); MS (ESI) *m*/*z* 347 (M + Na^+^, 100); HRMS (ESI) *m*/*z*: [M + Na]^+^ calcd for C_17_H_13_FN_4_O_2_Na 347.0915; found 347.0913.

### (2*S*)-2-Amino-3-{5′-[(4‴-cyanophenyl)ethynyl]-1*H*-benzo[*d*][1.2.3]triazol-1′-yl}propanoic Acid Hydrochloride (**12f**)

(2*S*)-2-Amino-3-{5′-[(4‴-cyanophenyl)ethynyl]-1*H*-benzo[*d*][1.2.3]triazol-1′-yl}propanoic acid hydrochloride (**12f**) was prepared as described for **12a** using methyl (2*S*)-2-[(*tert*-butoxycarbonyl)amino]-3-{5′-[(4‴-cyanophenyl)ethynyl]-1*H*-benzo[*d*][1.2.3]triazol-1′-yl}propanoate (**11f**) (0.100 g, 0.220 mmol) and cesium carbonate (0.0950 g, 0.290 mmol). This gave (2*S*)-2-[(*tert*-butoxycarbonyl)amino]-3-{5′-[(4‴-cyanophenyl)ethynyl]-1*H*-benzo[*d*][1.2.3]triazol-1′-yl}propanoic acid (0.0895 g, 94%) as a yellow solid. This was used for the next reaction without any further purification. To a solution of (2*S*)-2-[(*tert*-butoxycarbonyl)amino]-3-{5′-[(4‴-cyanophenyl)ethynyl]-1*H*-benzo[*d*][1.2.3]triazol-1′-yl}propanoic acid (0.0500 g, 0.0890 mmol) in acetonitrile (0.1 mL) was added 2 M aqueous hydrochloric acid (1 mL). The reaction mixture was stirred at room temperature for 3 h and concentrated *in vacuo*. Purification by recrystallization from methanol and diethyl ether gave (2*S*)-2-amino-3-{5′-[(4‴-cyanophenyl)ethynyl]-1*H*-benzo[*d*][1.2.3]triazol-1′-yl}propanoic acid hydrochloride (**12f**) as a yellow solid (0.0300 g, 88%). Mp 215–218 °C; IR (neat) 2862, 2230, 1748, 1600, 1497, 1250, 814 cm^–1^; [α]_D_^20^ +12.4 (*c* 0.2, MeOH); ^1^H NMR (DMSO-*d*_6_, 500 MHz): δ 8.39 (s, 1H), 8.09 (d, 1H, *J* = 8.5 Hz), 7.93 (d, 2H, *J* = 8.5 Hz), 7.81–7.76 (m, 3H), 5.24–5.21 (m, 2H), 4.65 (t, 1H, *J* = 5.0 Hz); ^13^C{^1^H} NMR (DMSO-*d*_6_, 126 MHz): δ 47.4 (CH_2_), 52.2 (CH), 88.2 (C), 93.5 (C), 111.5 (C), 112.3 (CH), 117.8 (C), 118.9 (C), 123.4 (CH), 127.5 (C), 131.2 (CH), 132.7 (2 × CH), 133.2 (2 × CH), 134.3 (C), 145.4 (C), 168.9 (C); MS (ESI) *m*/*z* 332 (M + H^+^, 100); HRMS (ESI) *m*/*z*: [M + H]^+^ calcd for C_18_H_13_N_5_O_2_H 332.1142; found 332.1140.

### Methyl (2*S*,1″*E*)-2-[(*tert*-butyloxycarbonyl)amino]-3-(5′-styryl-1*H*-benzo[*d*][1,2,3]triazol-1′-yl)propanoate (**13a**)

To a dry microwave vial containing zinc (0.0470 g, 0.720 mmol) and bis(triphenylphosphine)palladium(II) dichloride (0.0337 g, 0.0480 mmol), under argon, was added a solution of methyl (2*S*)-2-[(*tert*-butoxycarbonyl)amino]-3-[5′-(phenylethynyl)-1*H*-benzo[*d*][1.2.3]triazol-1′-yl]propanoate (**11a**) (0.100 g, 0.240 mmol) in tetrahydrofuran (1.2 mL). A 1 M solution of zinc iodide (0.240 mL, 0.240 mmol) in tetrahydrofuran was added and then the reaction mixture was degassed, purged with hydrogen, and stirred at 40 °C for 20 h. The reaction mixture was allowed to cool to room temperature and diluted with ethyl acetate (40 mL), washed with water (3 × 20 mL), dried (MgSO_4_), and concentrated *in vacuo*. Purification by flash column chromatography, eluting with 70% diethyl ether in hexane gave methyl (2*S*,1″*E*)-2-[(*tert*-butyloxycarbonyl)amino]-3-(5′-styryl-1*H*-benzo[*d*][1,2,3]triazol-1′-yl)propanoate (**13a**) as an off-white solid (0.0660 g, 65%). Mp 170–172 °C; IR (neat) 3368, 2978, 2361, 1755, 1690, 1501, 1161, 748 cm^–1^; [α]_D_^22^ +7.0 (*c* 0.5, CHCl_3_); ^1^H NMR (CDCl_3_, 400 MHz): δ 8.08 (br s, 1H), 7.73 (dd, 1H, *J* = 8.7, 0.9 Hz), 7.57–7.53 (m, 2H), 7.50 (d, 1H, *J* = 8.7 Hz), 7.44–7.34 (m, 2H), 7.31–7.27 (m, 1H), 7.25 (d, 1H, *J* = 16.3 Hz), 7.16 (d, 1H, *J* = 16.3 Hz), 5.39 (d, 1H, *J* = 6.8 Hz), 5.15–5.04 (m, 2H), 4.81 (dt, 1H, *J* = 6.8, 4.4 Hz), 3.77 (s, 3H), 1.43 (s, 9H); ^13^C{^1^H} NMR (CDCl_3_, 101 MHz): δ 169.6 (C), 155.1 (C), 146.4 (C), 137.0 (C), 134.1 (C), 133.6 (C), 129.5 (CH), 128.8 (2 × CH), 127.91 (CH), 127.94 (CH), 126.6 (2 × CH), 126.4 (CH), 117.7 (CH), 109.5 (CH), 80.7 (C), 53.9 (CH), 53.1 (CH_3_), 48.7 (CH_2_), 28.3 (3 × CH_3_); MS (ESI) *m*/*z* 423 (M + H^+^, 100); HRMS (ESI) *m*/*z*: [M + H]^+^ calcd for C_23_H_26_N_4_O_4_H 423.2027; found 423.2035.

### Methyl (2*S*,1″*E*)-2-[(*tert*-butyloxycarbonyl)amino]-3-(5′-[(4‴-methoxyphenyl)ethenyl]-1*H*-benzo[*d*][1,2,3]triazol-1′-yl)propanoate (**13b**)

Methyl (2*S*,1″*E*)-2-[(*tert*-butyloxycarbonyl)amino]-3-(5′-[(4‴-methoxyphenyl)ethenyl]-1*H*-benzo[*d*][1,2,3]triazol-1′-yl)propanoate (**13b**) was synthesized as described for **13a** using zinc (0.0610 g, 0.930 mmol), bis(triphenylphosphine)palladium(II) dichloride (0.0436 g, 0.0620 mmol), methyl (2*S*)-2-[(*tert*-butoxycarbonyl)amino]-3-{5′-[(4‴-methoxyphenyl)ethynyl]-1*H*-benzo[*d*][1.2.3]triazol-1′-yl}propanoate (**11b**) (0.140 g, 0.310 mmol), tetrahydrofuran (1.7 mL), and a 1 M solution of zinc iodide (0.310 mL, 0.310 mmol) in tetrahydrofuran. Purification by flash column chromatography, eluting with 75% diethyl ether in hexane gave methyl (2*S*,1″*E*)-2-[(*tert*-butyloxycarbonyl)amino]-3-(5′-[(4‴-methoxyphenyl)ethenyl]-1*H*-benzo[*d*][1,2,3]triazol-1′-yl)propanoate (**13b**) as a yellow solid (0.0895 g, 64%). Mp 164–168 °C; IR (neat) 3306, 2967, 2361, 1740, 1701, 1605, 1512, 1300, 822 cm^–1^; [α]_D_^21^ +15.0 (*c* 0.2, CHCl_3_); ^1^H NMR (CDCl_3_, 500 MHz): δ 8.05 (s, 1H), 7.71 (d, 1H, *J* = 8.7 Hz), 7.53–7.46 (m, 3H), 7.12 (s, 2H), 6.92 (d, 2H, *J* = 8.4 Hz), 5.37 (d, 1H, *J* = 6.7 Hz), 5.16–5.04 (m, 2H), 4.81 (dt, 1H, *J* = 6.7, 4.2 Hz), 3.85 (s, 3H), 3.77 (s, 3H), 1.44 (s, 9H); ^13^C{^1^H} NMR (CDCl_3_, 126 MHz): δ 169.7 (C), 159.5 (C), 155.1 (C), 146.4 (C), 134.5 (C), 133.3 (C), 129.8 (C), 129.0 (CH), 127.8 (2 × CH), 126.3 (CH), 125.8 (CH), 117.1 (CH), 114.2 (2 × CH), 109.5 (CH), 80.6 (C), 55.4 (CH_3_), 53.9 (CH), 53.1 (CH_3_), 48.7 (CH_2_), 28.3 (3 × CH_3_); MS (ESI) *m*/*z* 475 (M + Na^+^, 100); HRMS (ESI) *m*/*z*: [M + Na]^+^ calcd for C_24_H_28_N_4_O_5_Na 475.1952; found 475.1954.

### Methyl (2*S*,1″*E*)-2-[(*tert*-butyloxycarbonyl)amino]-3-(5′-[(4‴-fluorophenyl)ethenyl]-1*H*-benzo[*d*][1,2,3]triazol-1′-yl)propanoate (**13c**)

Methyl (2*S*,1″*E*)-2-[(tert-butyloxycarbonyl)amino]-3-(5′-[(4‴-fluorophenyl)ethenyl]-1*H*-benzo[*d*][1,2,3]triazol-1′-yl)propanoate (**13c**) was synthesized as described for **13a** using zinc (0.0445 g, 0.680 mmol), bis(triphenylphosphine)palladium(II) dichloride (0.0323 g, 0.0460 mmol), methyl (2*S*)-2-[(*tert*-butyloxycarbonyl)amino]-3-(5′-[(4‴-fluorophenyl)ethynyl]-1*H*-benzo[*d*][1.2.3]triazol-1′-yl)propanoate (**11e**) (0.100 g, 0.230 mmol), tetrahydrofuran (1.4 mL), and a 1 M solution of zinc iodide (0.230 mL, 0.230 mmol) in tetrahydrofuran. Purification by flash column chromatography, eluting with 70% diethyl ether in hexane gave methyl (2*S*,1″*E*)-2-[(*tert*-butyloxycarbonyl)amino]-3-(5′-[(4‴-fluorophenyl)ethenyl]-1*H*-benzo[*d*][1,2,3]triazol-1′-yl)propanoate (**13c**) as a yellow solid (0.0580 g, 57%). Mp 162–165 °C; IR (neat) 3383, 2982, 2361, 1759, 1690, 1508, 1157, 829 cm^–1^; [α]_D_^24^ +6.2 (*c* 0.2, CHCl_3_); ^1^H NMR (DMSO-*d*_6_, 400 MHz): δ 8.16 (br s, 1H), 7.90 (dd, 1H, *J* = 8.8, 0.9 Hz), 7.85 (d, 1H, *J* = 8.8 Hz), 7.68 (dd, 2H, *J* = 8.9, 5.6 Hz), 7.43 (d, 1H, *J* = 9.0 Hz), 7.40 (s, 2H), 7.24 (t, 2H, *J* = 8.9 Hz), 5.08 (dd, 1H, *J* = 14.4, 4.5 Hz), 4.95 (dd, 1H, *J* = 14.4, 9.0 Hz), 4.62 (td, 1H, *J* = 9.0, 4.5 Hz), 3.67 (s, 3H), 1.22 (s, 9H); ^13^C{^1^H} NMR (DMSO-*d*_6_, 101 MHz): δ 170.5 (C), 162.2 (d, ^1^*J*_C–F_ 244.9 Hz, C), 155.4 (C), 146.3 (C), 134.1 (d, ^4^*J*_C–F_ 3.2 Hz, C), 133.9 (C), 133.6 (C), 128.8 (d, ^3^*J*_C–F_ 8.0 Hz, 2 × CH), 128.4 (CH), 128.0 (CH), 126.3 (CH), 117.2 (CH), 116.1 (d, ^2^*J*_C–F_ 21.6 Hz, 2 × CH), 111.4 (CH), 79.1 (C), 53.9 (CH), 52.8 (CH_3_), 48.3 (CH_2_), 28.4 (3 × CH_3_); MS (ESI) *m*/*z* 463 (M + Na^+^, 100); HRMS (ESI) *m*/*z*: [M + Na]^+^ calcd for C_23_H_25_FN_4_O_4_Na 463.1752; found 463.1752.

### (2*S*,1″*E*)-2-Amino-3-(5′-styryl-1*H*-benzo[*d*][1,2,3]triazol-1′-yl)propanoic Acid Hydrochloride (**14a**)

To a solution of methyl (2*S*,1″*E*)-2-[(*tert*-butyloxycarbonyl)amino]-3-(5′-styryl-1*H*-benzo[*d*][1,2,3]triazol-1′-yl)propanoate (**13a**) (0.0500 g, 0.120 mmol) in a mixture of methanol (1.5 mL) and 1,4-dioxane (1.5 mL) was added a solution of cesium carbonate (0.0500 g, 0.150 mmol) in water (0.75 mL). The reaction mixture was stirred at room temperature for 20 h and then concentrated *in vacuo*. The resulting residue was dissolved in water (50 mL) and acidified to pH 1 with 1 M aqueous hydrochloric acid. The aqueous layer was extracted with dichloromethane (3 × 30 mL), dried (MgSO_4_), and concentrated *in vacuo* to give (2*S*,1″*E*)-2-[(*tert*-butyloxycarbonyl)amino]-3-(5′-styryl-1*H*-benzo[*d*][1,2,3]triazol-1′-yl)propanoic acid as a yellow solid (0.0430 g, 88%). This was used for the next reaction without further purification. To a solution of (2*S*,1″*E*)-2-[(*tert*-butyloxycarbonyl)amino]-3-(5′-styryl-1*H*-benzo[*d*][1,2,3]triazol-1′-yl)propanoic acid (0.0300 g, 0.0700 mmol) in 1,4-dioxane (0.1 mL) was added 2 M aqueous hydrochloric acid (2.5 mL). The reaction mixture was stirred at room temperature for 3 h and then concentrated *in vacuo*. This gave (2*S*,1″*E*)-2-amino-3-(5′-styryl-1*H*-benzo[*d*][1,2,3]triazol-1′-yl)propanoic acid hydrochloride (**14a**) as an off-white solid (0.0230 g, 92%). Mp 215–219 °C; IR (neat) 3368, 2955, 2361, 1748, 1690, 1501, 1250, 1204, 1161, 691 cm^–1^; [α]_D_^22^ +5.6 (*c* 0.1, MeOH); ^1^H NMR (CD_3_OD, 500 MHz): δ 8.09 (s, 1H), 7.93 (d, 1H, *J* = 8.5 Hz), 7.82 (d, 1H, *J* = 8.5 Hz), 7.59 (br d, 2H, *J* = 7.5 Hz), 7.43–7.23 (m, 5H), 5.34 (dd, 1H, *J* = 14.9, 4.9 Hz), 5.25 (dd, 1H, *J* = 14.9, 2.3 Hz), 4.74 (br s, 1H); ^13^C{^1^H} NMR (CD_3_OD, 126 MHz): δ 167.6 (C), 146.2 (C), 137.1 (C), 135.1 (C), 133.1 (C), 129.7 (CH), 128.4 (2 × CH), 127.6 (CH), 127.2 (CH), 126.7 (CH), 126.3 (2 × CH), 116.4 (CH), 110.0 (CH), 52.1 (CH), 46.7 (CH_2_); MS (ESI) *m*/*z* 309 (M + H^+^, 100); HRMS (ESI) *m*/*z*: [M + H]^+^ calcd for C_17_H_16_N_4_O_2_H 309.1346; found 309.1344.

### (2*S*,1″*E*)-2-Amino-3-(5′-[(4‴-methoxyphenyl)ethenyl]-1*H*-benzo[*d*][1,2,3]triazol-1′-yl)propanoic Acid Hydrochloride (**14b**)

(2*S*,1″*E*)-2-Amino-3-(5′-[(4‴-methoxyphenyl)ethenyl]-1*H*-benzo[*d*][1,2,3]triazol-1′-yl)propanoic acid hydrochloride (**14b**) was synthesized as described for **14a** using methyl (2*S*,1″*E*)-2-[(*tert*-butyloxycarbonyl)amino]-3-(5′-[(4‴-methoxyphenyl)ethenyl]-1*H*-benzo[*d*][1,2,3]triazol-1′-yl)propanoate (**13b**) (0.0300 g, 0.0660 mmol) and cesium carbonate (0.0280 g, 0.0860 mmol). This gave (2*S*,1″*E*)-2-amino-3-(5′-[(4‴-methoxyphenyl)ethenyl]-1*H*-benzo[*d*][1,2,3]triazol-1′-yl)propanoic acid as an off-white solid (0.0250 g, 87%). This was used for the next reaction without further purification using (2*S*,1″*E*)-2-amino-3-(5′-[(4‴-methoxyphenyl)ethenyl]-1*H*-benzo[*d*][1,2,3]triazol-1′-yl)propanoic acid (0.0250 g, 0.0570 mmol), 1,4-dioxane (2 mL), and 6 M aqueous hydrochloric acid (1 mL). This gave (2*S*,1″*E*)-2-amino-3-(5′-[(4‴-methoxyphenyl)ethenyl]-1*H*-benzo[*d*][1,2,3]triazol-1′-yl)propanoic acid hydrochloride (**14b**) as a yellow solid (0.0220 g, 88%). Mp 210–213 °C; IR (neat) 3372, 2932, 2361, 1728, 1601, 1574, 1501, 1443, 1173, 1022 cm^–1^; [α]_D_^21^ +10.1 (*c* 0.1, MeOH); ^1^H NMR (DMSO-*d*_6_, 400 MHz): δ 8.71 (br s, 1H), 8.16 (s, 1H), 7.93–7.89 (m, 2H), 7.58 (d, 2H, *J* = 8.8 Hz), 7.36 (d, 1H, *J* = 16.5 Hz), 7.29 (d, 1H, *J* = 16.5 Hz), 6.98 (d, 2H, *J* = 8.8 Hz), 5.24 (dd, 1H, *J* = 15.2, 5.5 Hz), 5.17 (dd, 1H, *J* = 15.2, 4.9 Hz), 4.69–4.63 (m, 1H), 3.79 (s, 3H); ^13^C{^1^H} NMR (DMSO-*d*_6_, 101 MHz): δ 168.9 (C), 159.5 (C), 146.4 (C), 134.7 (C), 133.5 (C), 130.1 (C), 129.1 (CH), 128.3 (2 × CH), 126.5 (CH), 126.1 (CH), 116.7 (CH), 114.7 (2 × CH), 111.4 (CH), 55.7 (CH_3_), 52.2 (CH), 47.3 (CH_2_); MS (ESI) *m*/*z* 361 (M + Na^+^, 100); HRMS (ESI) *m*/*z*: [M + Na]^+^ calcd for C_18_H_18_N_4_O_3_Na 361.1271; found 361.1270.

### (2*S*,1″*E*)-2-Amino-3-(5′-[(4‴-fluorophenyl)ethenyl]-1*H*-benzo[*d*][1,2,3]triazol-1′-yl)propanoic Acid Hydrochloride (**14c**)

(2*S*,1″*E*)-2-Amino-3-(5′-[(4‴-fluorophenyl)ethenyl]-1*H*-benzo[*d*][1,2,3]triazol-1′-yl)propanoic acid hydrochloride (**14c**) was synthesized as described for **14a** using methyl (2*S*,1″*E*)-2-[(*tert*-butyloxycarbonyl)amino]-3-(5′-[(4‴-fluorophenyl)ethenyl]-1*H*-benzo[*d*][1,2,3]triazol-1′-yl)propanoate (**13c**) (0.0800 g, 0.180 mmol) and cesium carbonate (0.0770 g, 0.240 mmol). This gave (2*S*,1″*E*)-2-[(*tert*-butyloxycarbonyl)amino]-3-(5′-[(4‴-fluorophenyl)ethenyl]-1*H*-benzo[*d*][1,2,3]triazol-1′-yl)propanoic acid as an off-white solid (0.0690 g, 90%). This was used for the next reaction without further purification using (2*S*,1″*E*)-2-[(*tert*-butyloxycarbonyl)amino]-3-(5′-[(4‴-fluorostyryl)ethenyl]-1*H*-benzo[*d*][1,2,3]triazol-1′-yl)propanoic acid (0.0500 g, 0.117 mmol), 1,4-dioxane (0.2 mL), and 2 M aqueous hydrochloric acid (2.5 mL). This gave (2*S*,1″*E*)-2-amino-3-(5′-[(4‴-fluorophenyl)ethenyl]-1*H*-benzo[*d*][1,2,3]triazol-1′-yl)propanoic acid hydrochloride (**14c**) as an off-white solid (0.0344 g, 81%). Mp 195–199 °C; IR (neat) 3372, 2951, 2361, 1728, 1597, 1497, 1200, 826 cm^–1^; [α]_D_^23^ +7.3 (*c* 0.1, MeOH); ^1^H NMR (DMSO-*d*_6_, 500 MHz): δ 8.21 (s, 1H), 7.97 (d, 1H, *J* = 9.0 Hz), 7.94 (d, 1H, *J* = 9.0 Hz), 7.69 (dd, 2H, *J* = 8.3, 5.8 Hz), 7.42 (s, 2H), 7.25 (t, 2H, *J* = 8.3 Hz), 5.27 (dd, 1H, *J* = 15.2, 5.2 Hz), 5.21 (dd, 1H, *J* = 15.2, 4.8 Hz), 4.69–4.61 (m, 1H); ^13^C{^1^H} NMR (DMSO-*d*_6_, 126 MHz): δ 168.9 (C), 162.2 (d, ^1^*J*_C–F_ 245.1 Hz, C), 146.3 (C), 134.2 (C), 134.1 (d, ^4^*J*_C–F_ 2.7 Hz, C), 133.7 (C), 128.9 (d, ^3^*J*_C–F_ 8.0 Hz, 2 × CH), 128.4 (CH), 128.3 (CH), 126.6 (CH), 117.2 (CH), 116.1 (d, ^2^*J*_C–F_ 21.5 Hz, 2 × CH), 111.5 (CH), 52.2 (CH), 47.3 (CH_2_); MS (ESI) *m*/*z* 327 (M + H^+^, 100); HRMS (ESI) *m*/*z*: [M + H]^+^ calcd for C_17_H_15_FN_4_O_2_H 327.1252; found 327.1260.

## Data Availability

The data underlying this study are available in the published article and its online Supporting Material.

## Supporting Information Available

Optimization studies; photophysical data for α-amino acids **12a**–**f** and **14a**–**c**; and ^1^H and ^13^C NMR spectra for all novel compounds (PDF)
