# Supplementary material for: Synthesis and Fluorescent Properties of Alkynyl- and Alkenyl-Fused Benzotriazole-Derived α-Amino Acids
Source: J Org Chem. 2023 Feb 7;88(4):2453–63. doi: 10.1021/acs.joc.2c02886 (PMC9942204; doi:10.1021/acs.joc.2c02886)

Supporting Information for:

Synthesis and Fluorescent Properties of Alkynyl- and Alkenyl-Fused Benzotriazole-Derived α-Amino Acids

*Leanne M. Riley, Toni N. Mclay and Andrew Sutherland**

School of Chemistry, The Joseph Black Building, University of Glasgow,

Glasgow G12 8QQ, United Kingdom.

Table of Contents

1. Optimisation of the Hydrogenation of Alkynyl α-Amino Acid **11b** S2
2. Photophysical Data for α-Amino Acids **12a**–**f** and **14a**–**c** S3–S13
3. ^{1}H and ^{13}C NMR Spectra for all Novel Compounds S14–S55

1. Optimisation of the Hydrogenation of Alkynyl α-Amino Acid 11b

Table S1

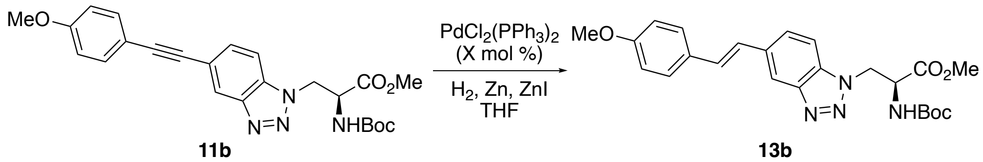

entry	catalyst loading (mol %)	temperature (°C)	time (h)	*Z*:*E* ratio	isolated yield (%)
1	5	25	112	1:2	17
2	20	30	48	*E* only	43
3	20	40	20	*E* only	64

2. Photophysical Data for α-Amino Acids 12a–f and 14a–c

Spectra were recorded at 5 μM in methanol using an excitation and emission slit width of 5 nm.

Absorption and Emission Spectra for 12a.

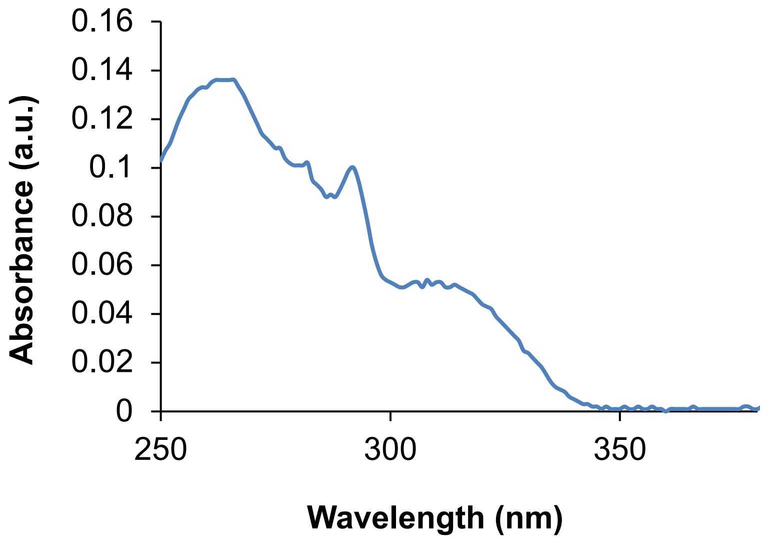

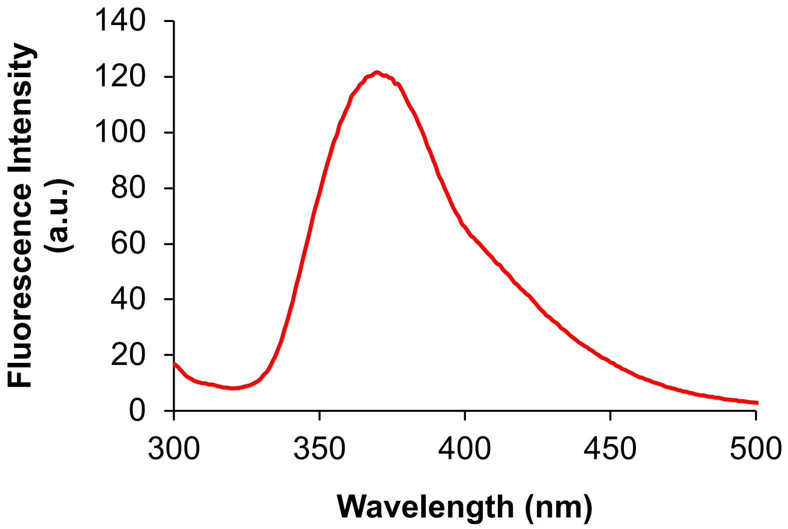

Absorption and Emission Spectra for 12b.

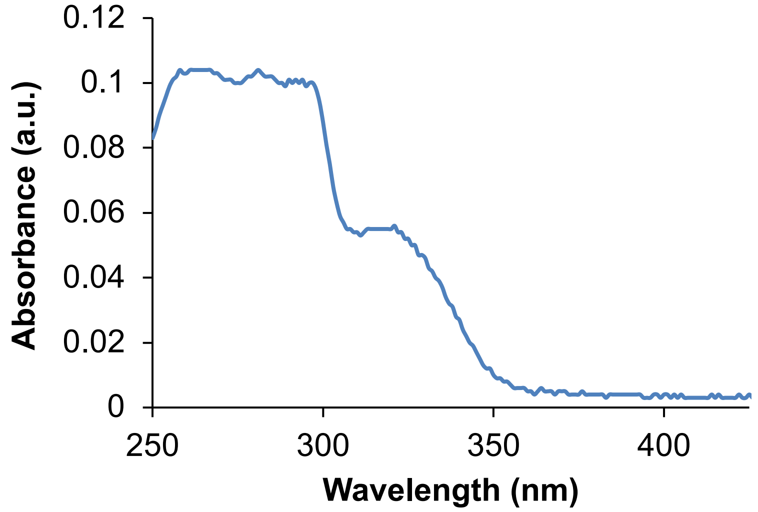

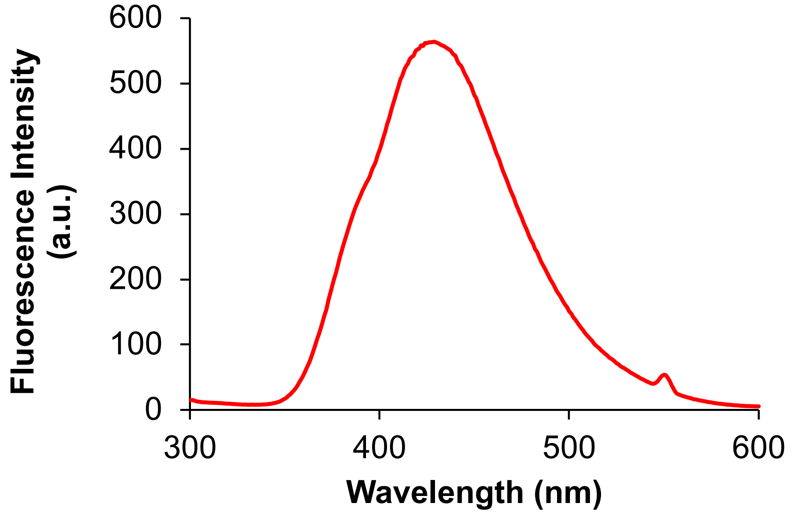

Absorption and Emission Spectra for 12c.

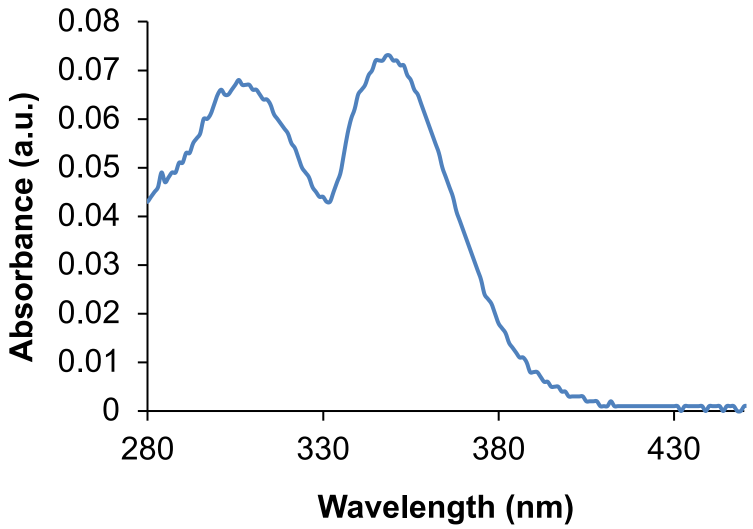

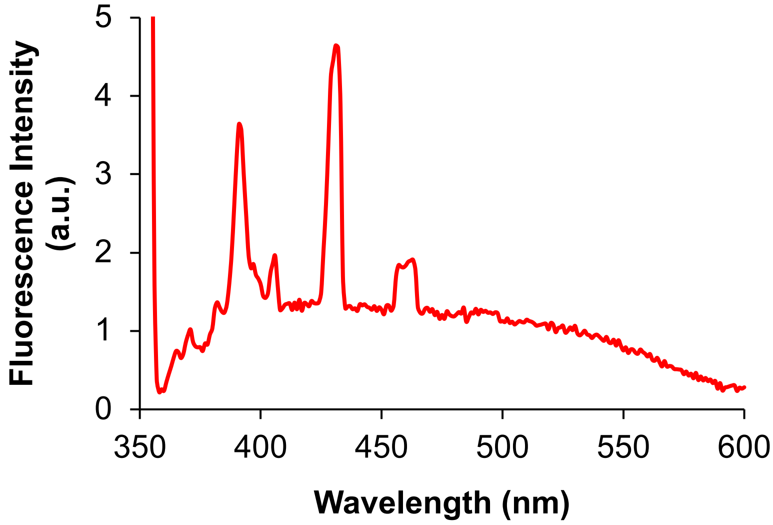

Absorption and Emission Spectra for 12d.

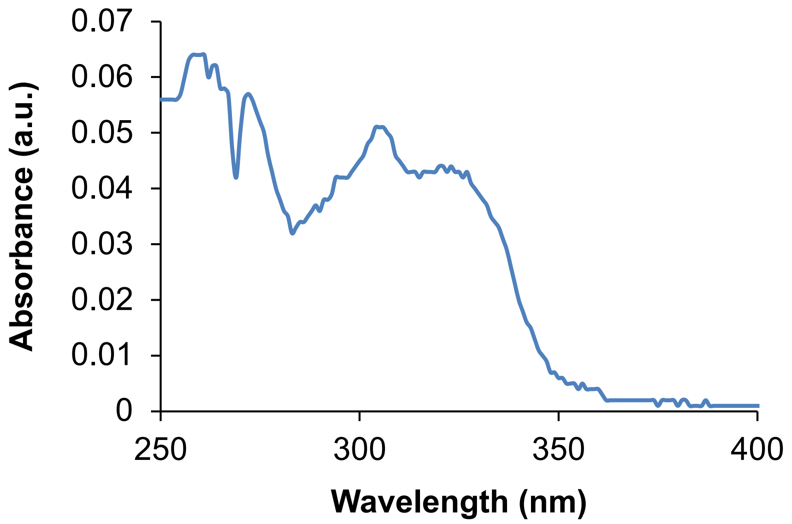

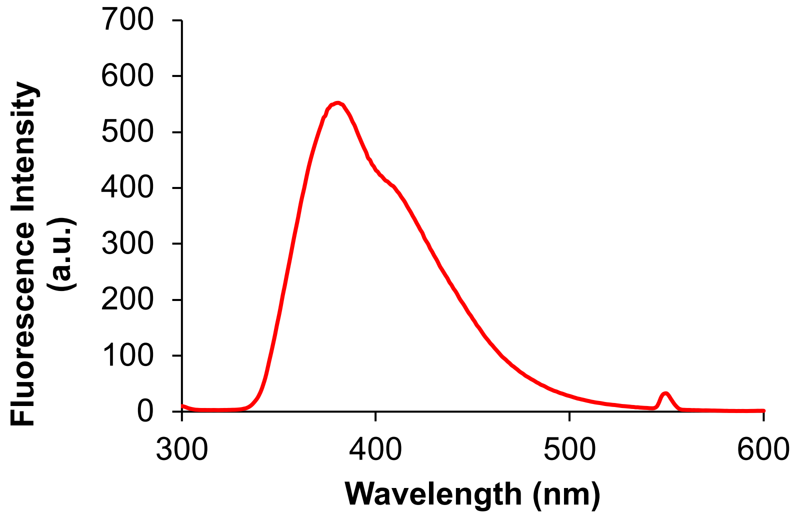

Absorption and Emission Spectra for 12e.

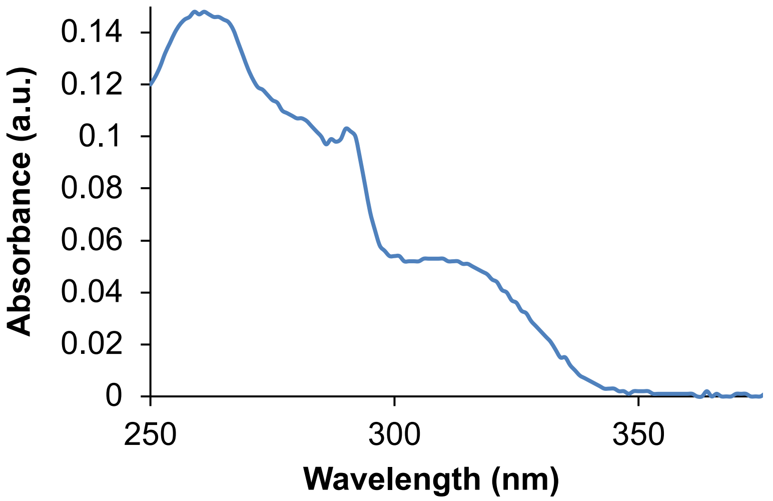

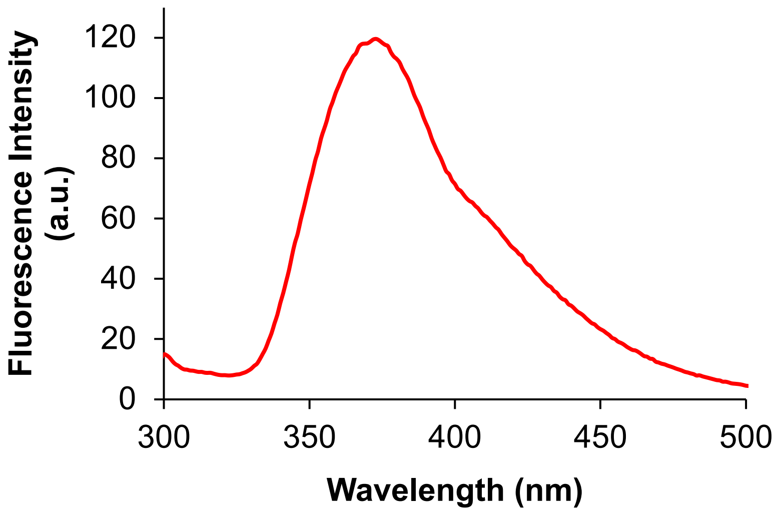

Absorption and Emission Spectra for 12f.

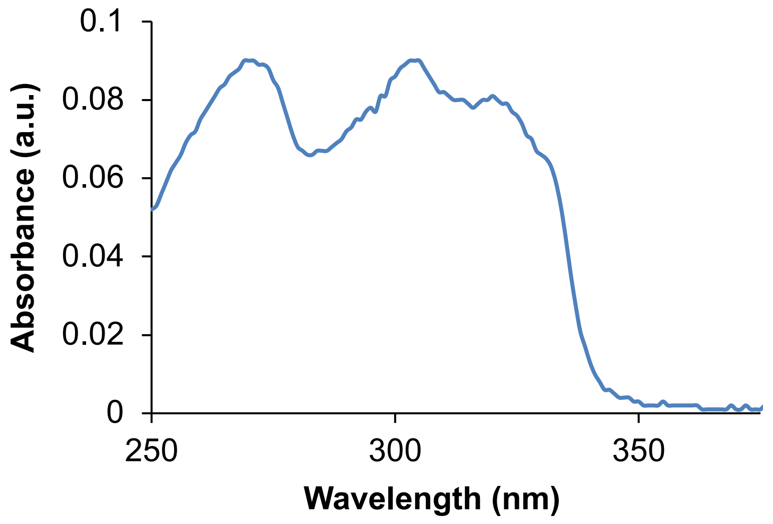

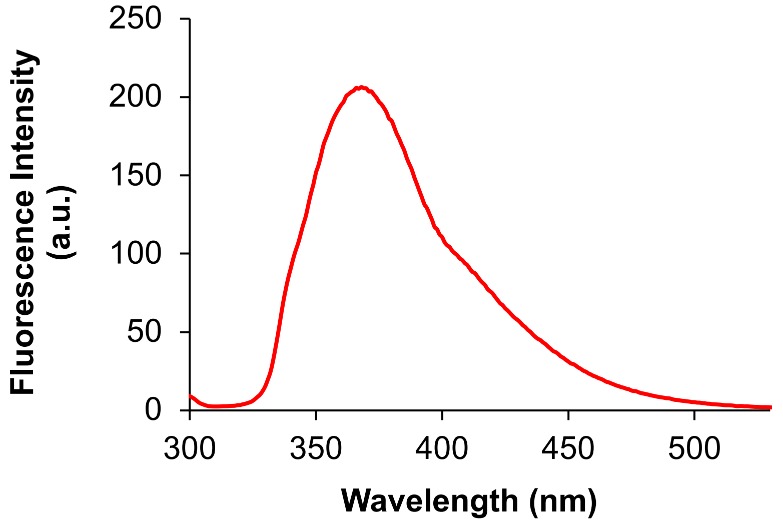

Absorption and Emission Spectra for 14a.

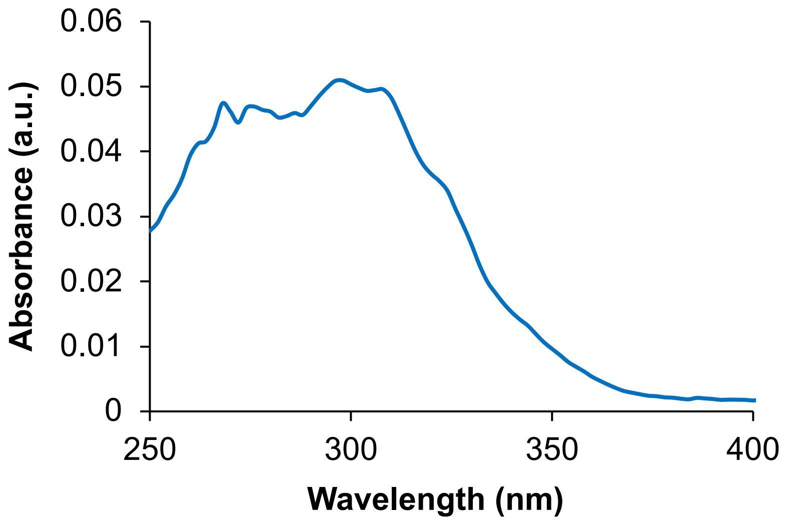

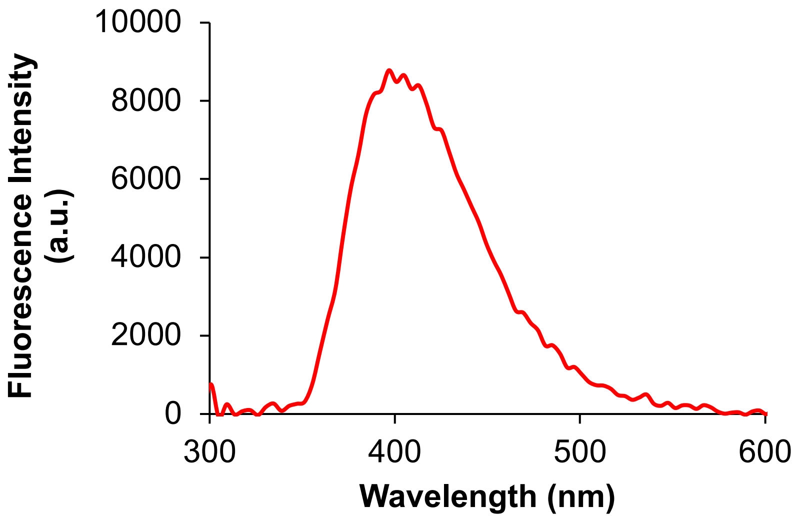

Absorption and Emission Spectra for 14b.

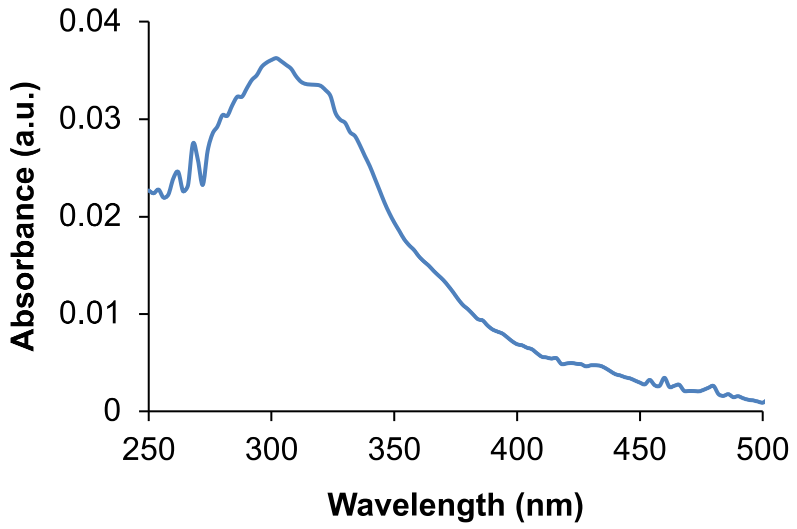

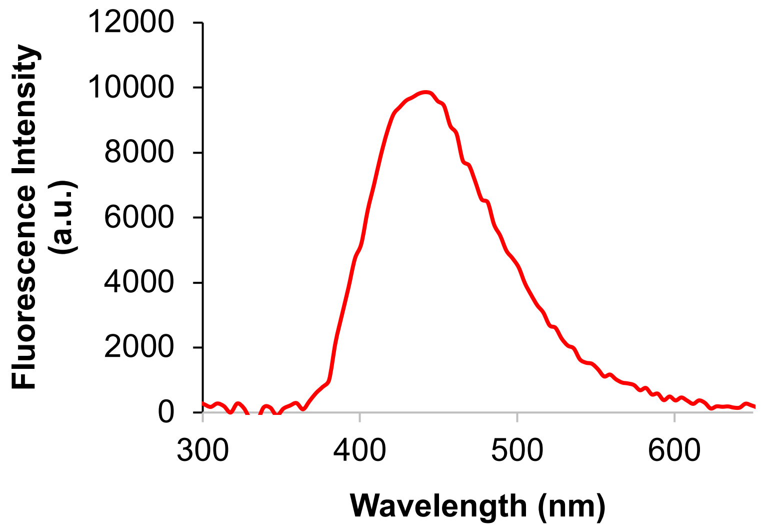

Absorption and Emission Spectra for 14c.

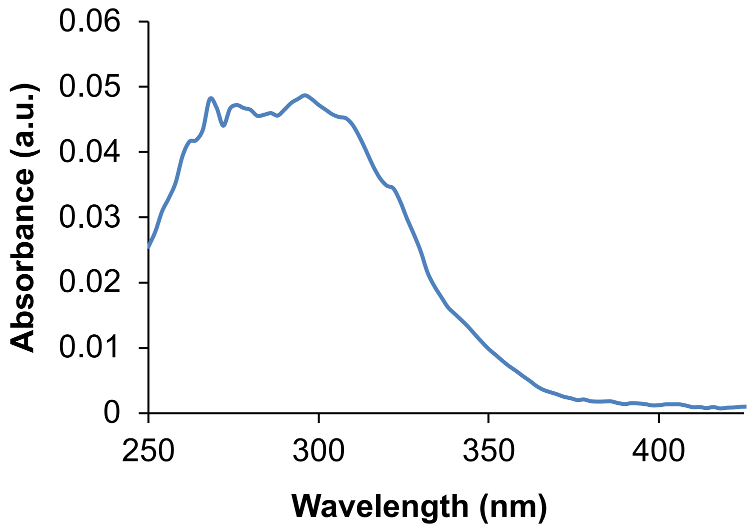

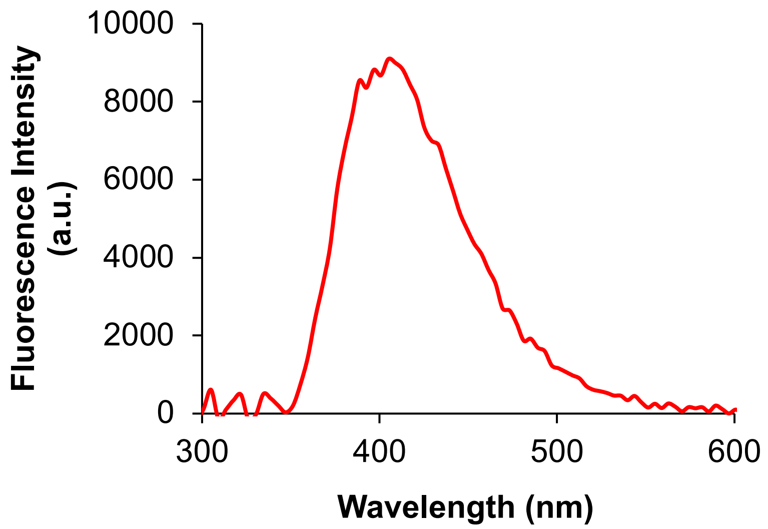

Absorption and Emission Spectra for the Solvatochromic Study of 12b.

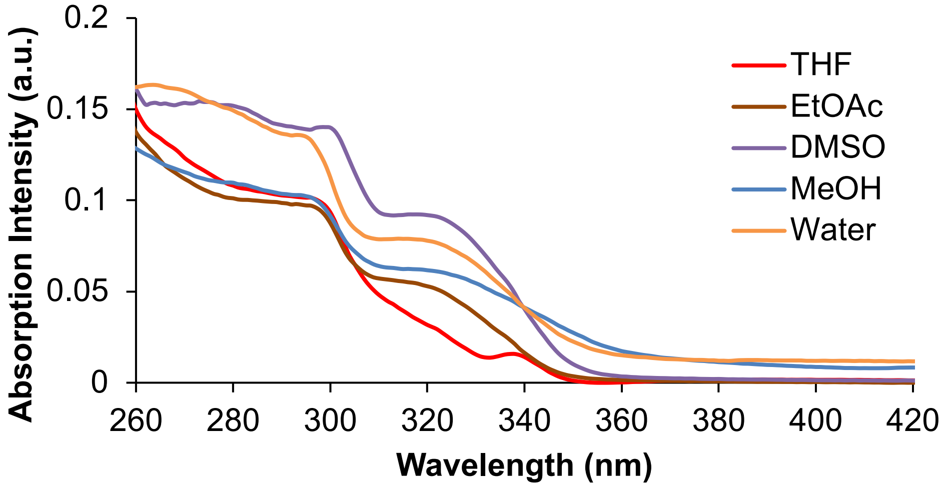

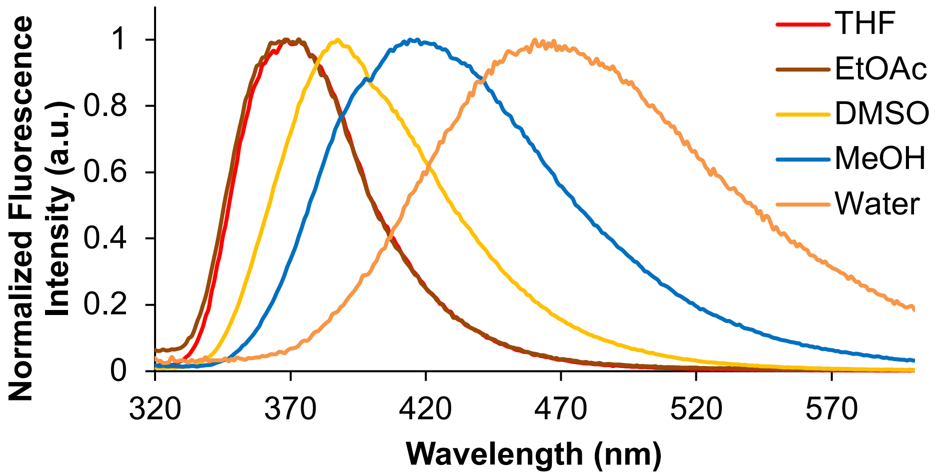

Lippert-Magata Graph for 12b.

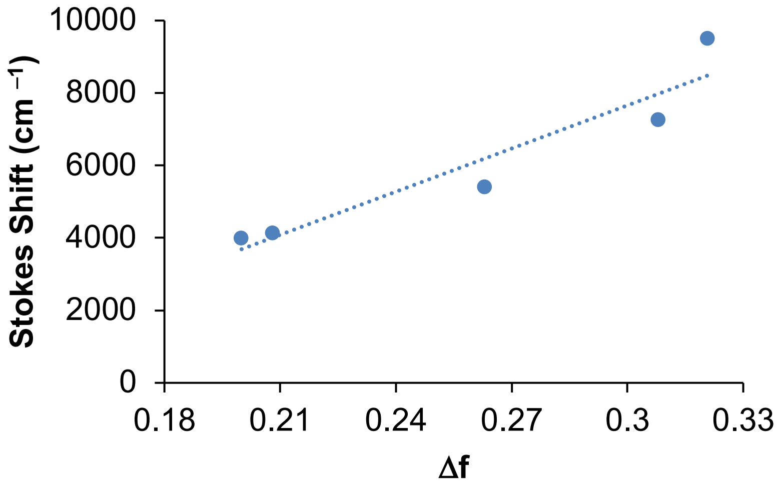

Absorption and Emission Spectra for the Solvatochromic Study of 12d.

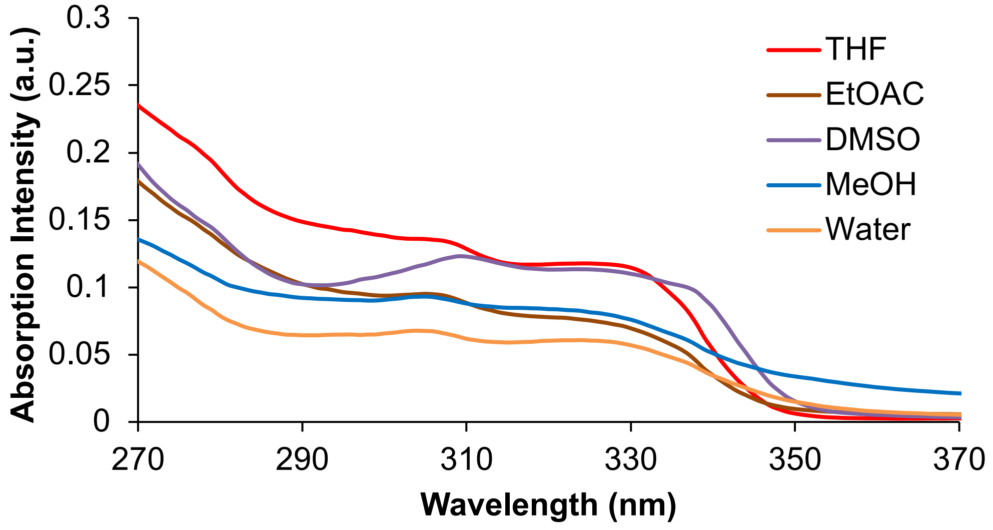

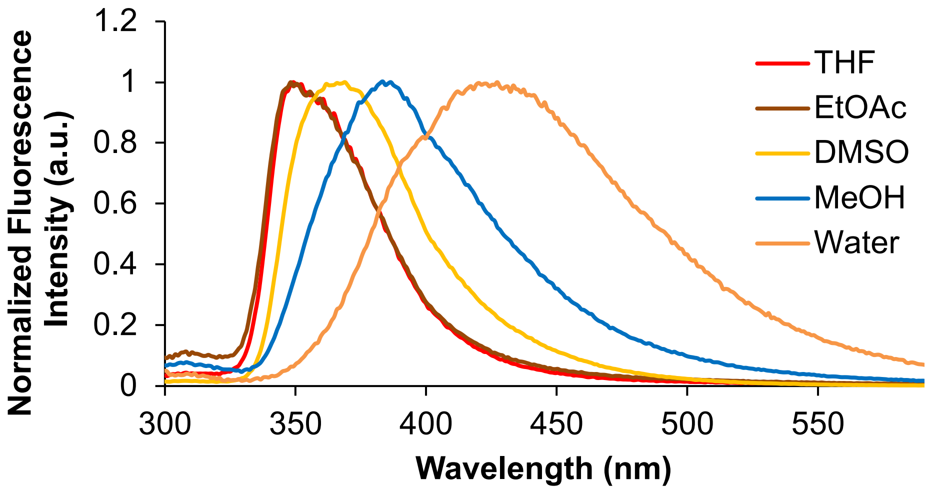

Lippert-Magata Graph for 12d.

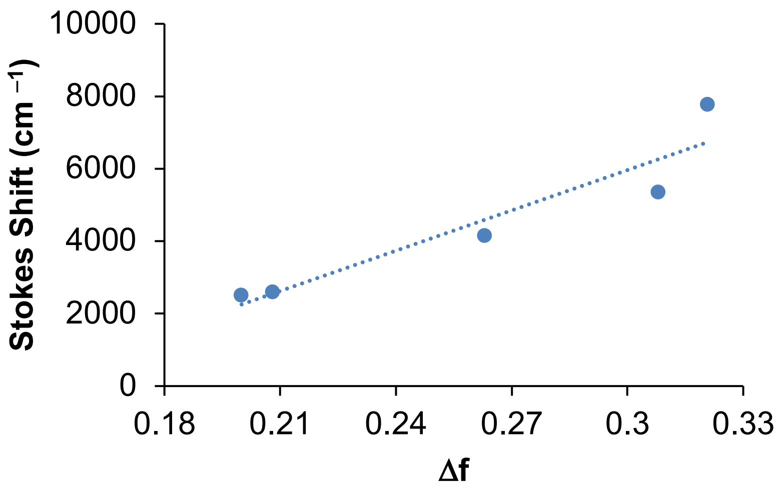

3. ^{1}H and ^{13}C NMR Spectra for all Novel Compounds

^{1}H NMR (400 MHz, $CDCl_3$)

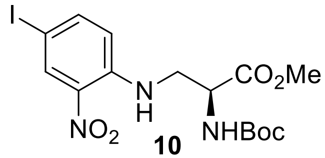

10

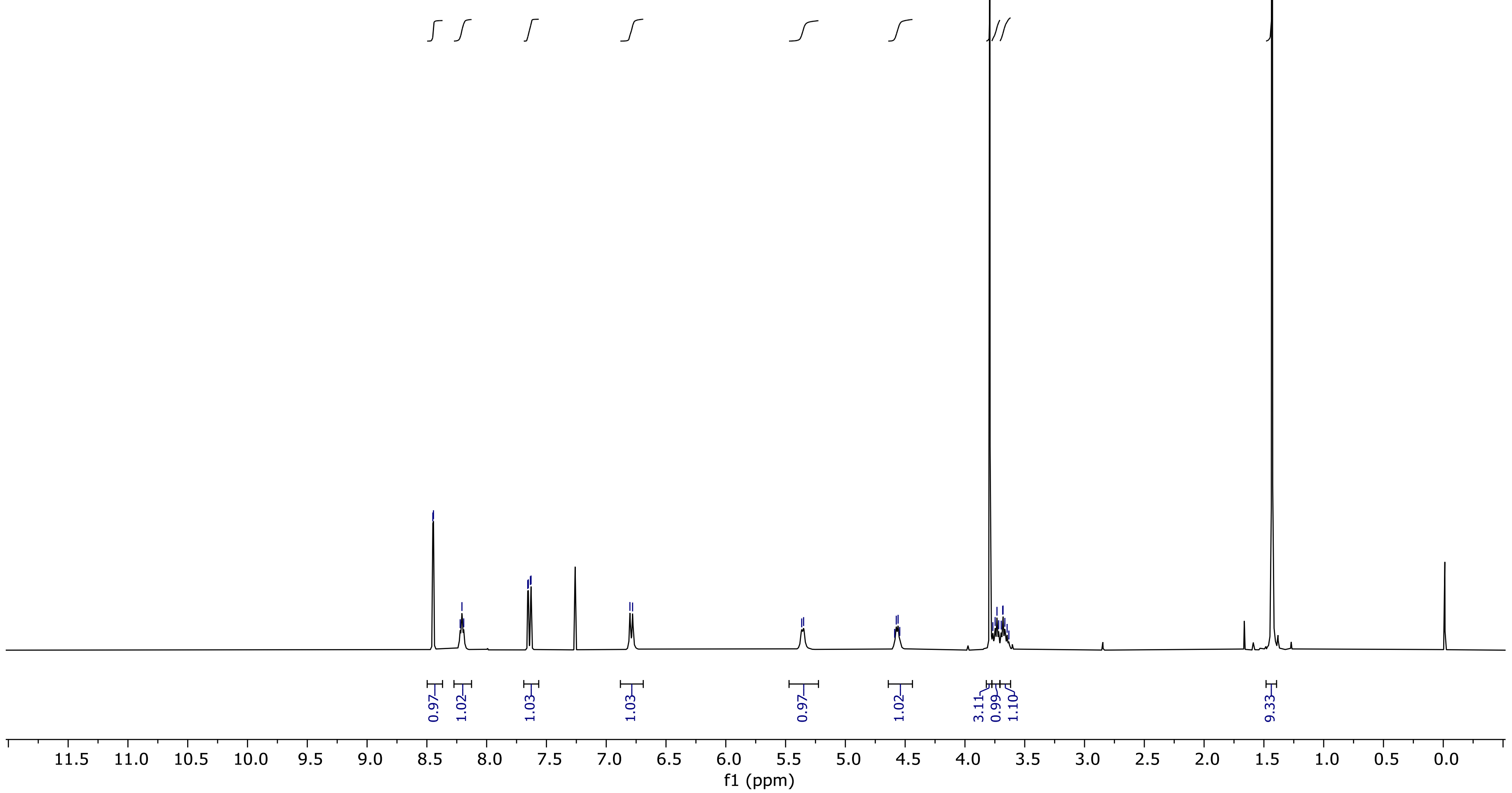

$^{13}C\{^{1}H\}$ NMR (101 MHz, $CDCl_3$)

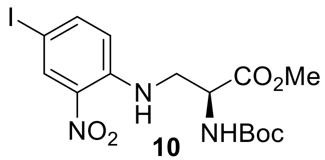

10

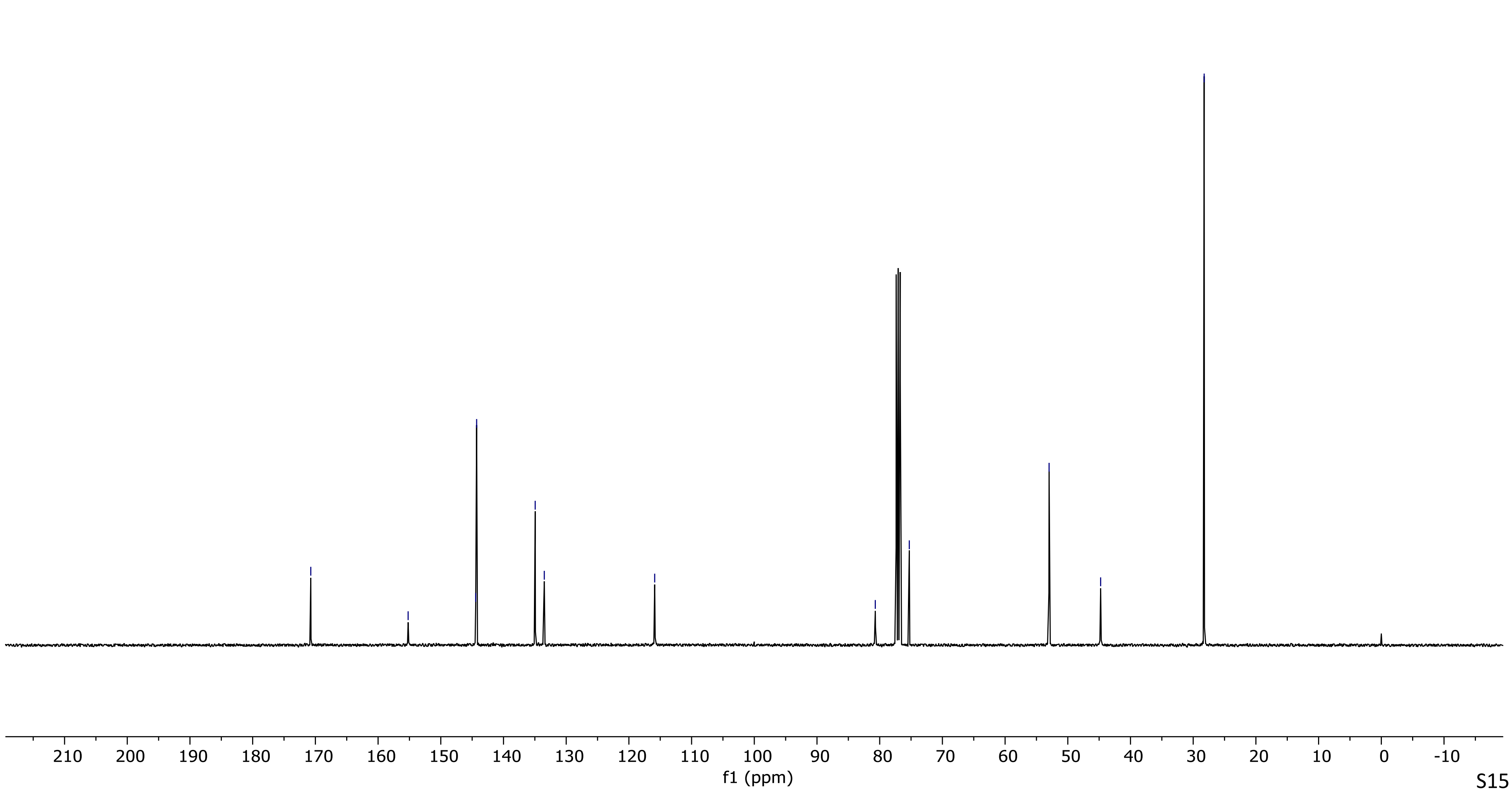

^{1}H NMR (400 MHz, $CDCl_3$)

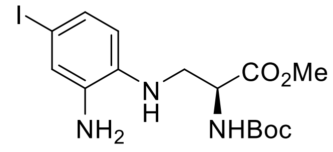

7

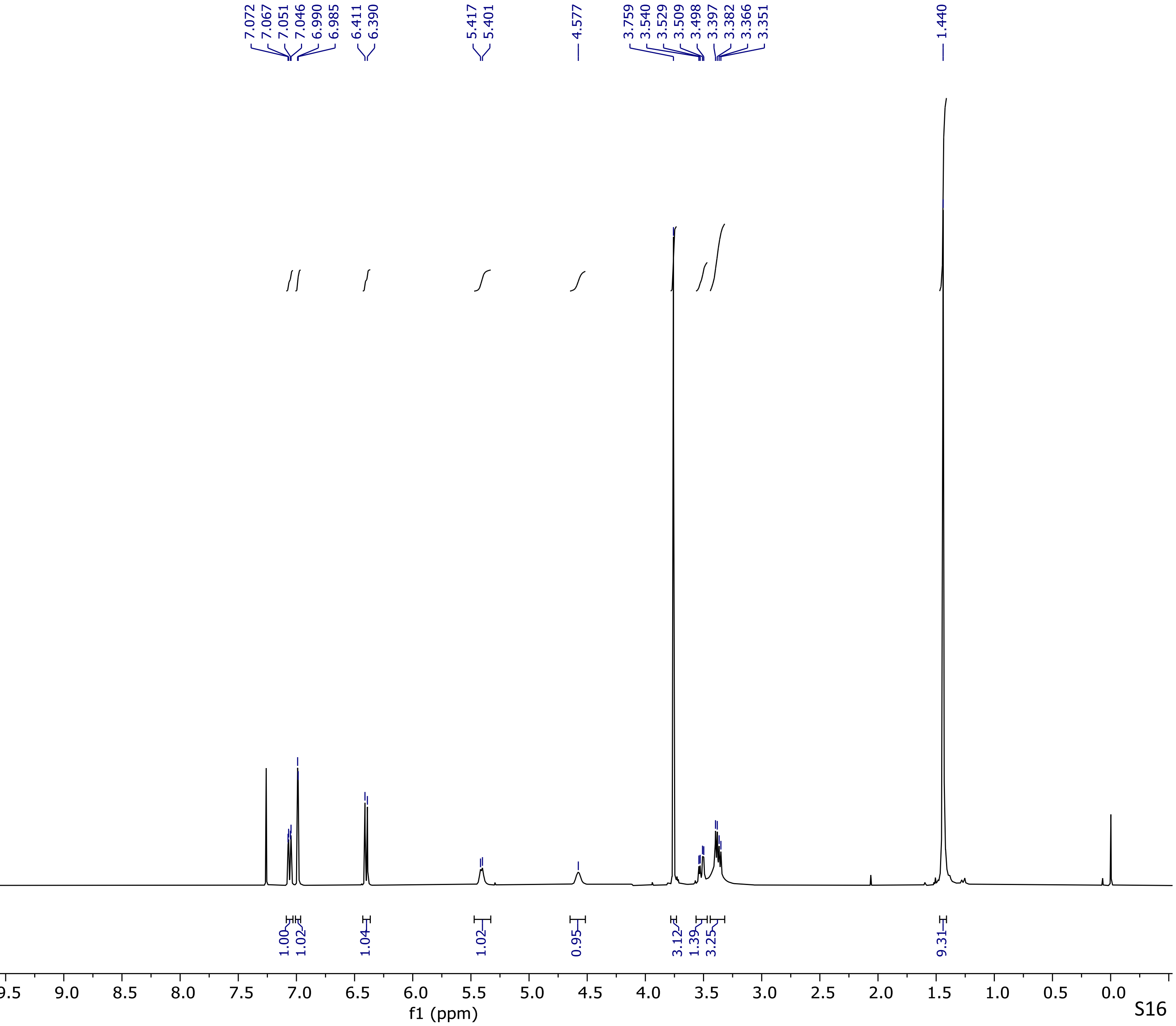

$^{13}C\{^1H\}$ NMR (101 MHz, $CDCl_3$)

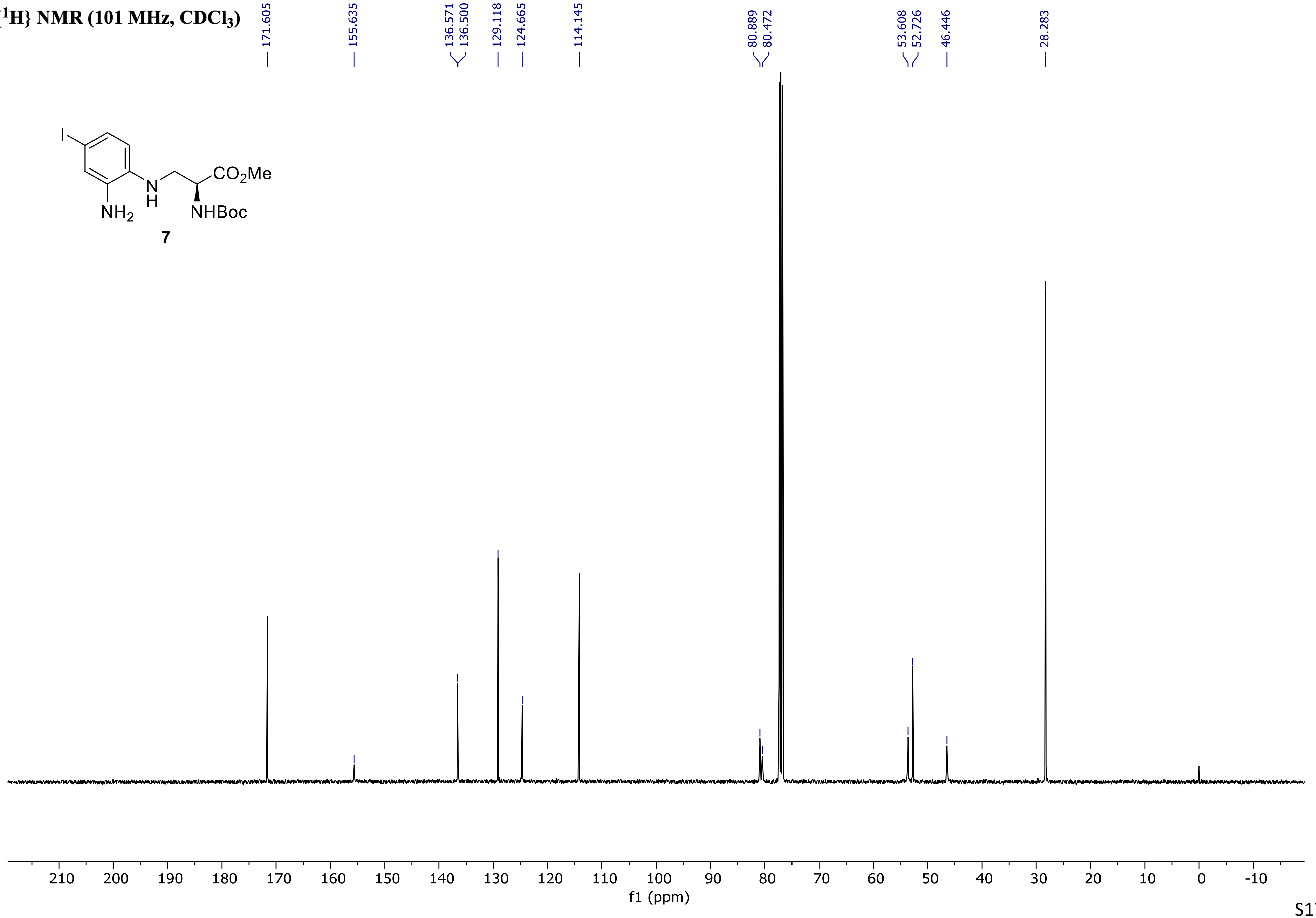

^{1}H NMR (400 MHz, $CDCl_3$)

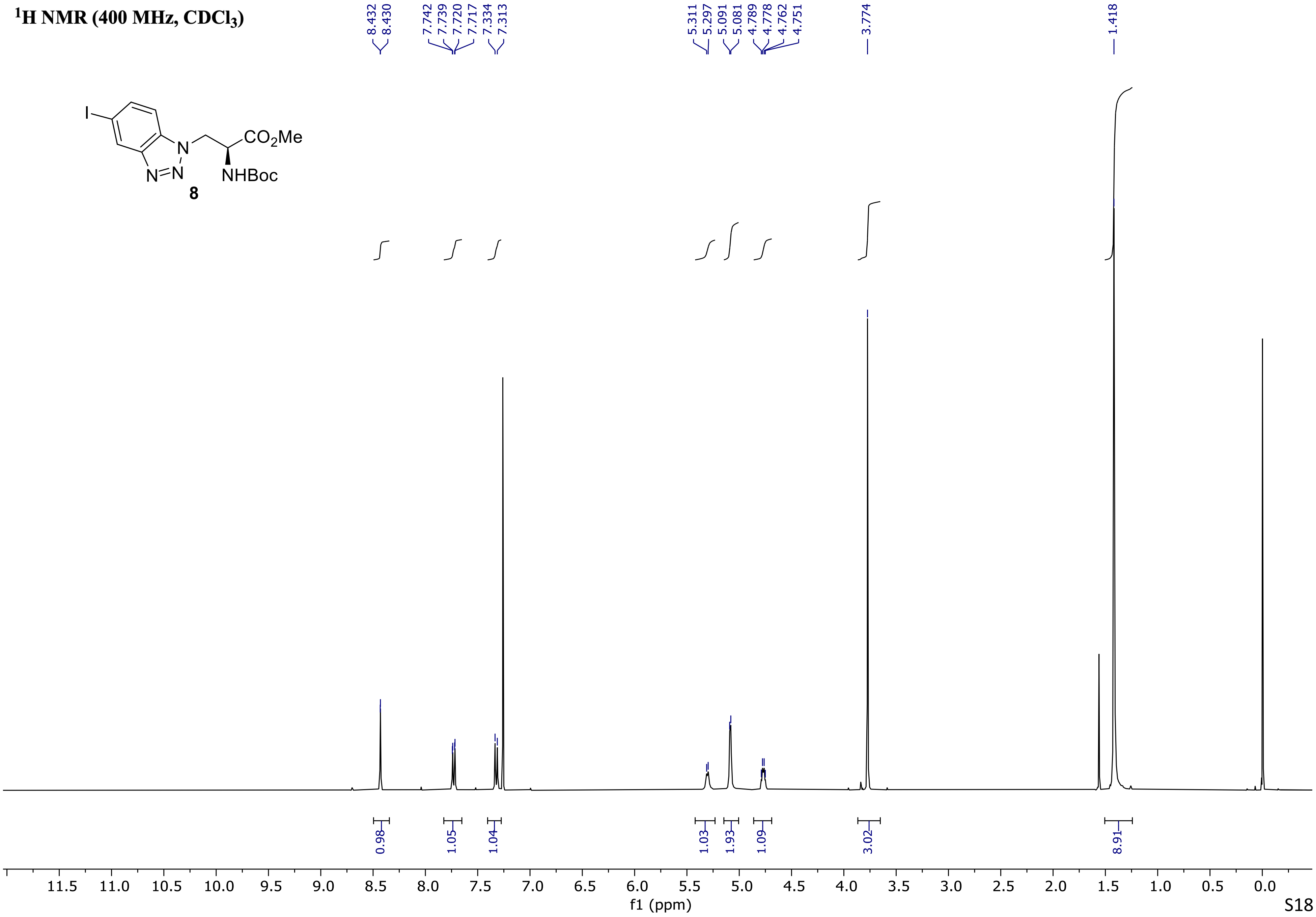

$^{13}C\{^{1}H\}$ NMR (101 MHz, $CDCl_3$)

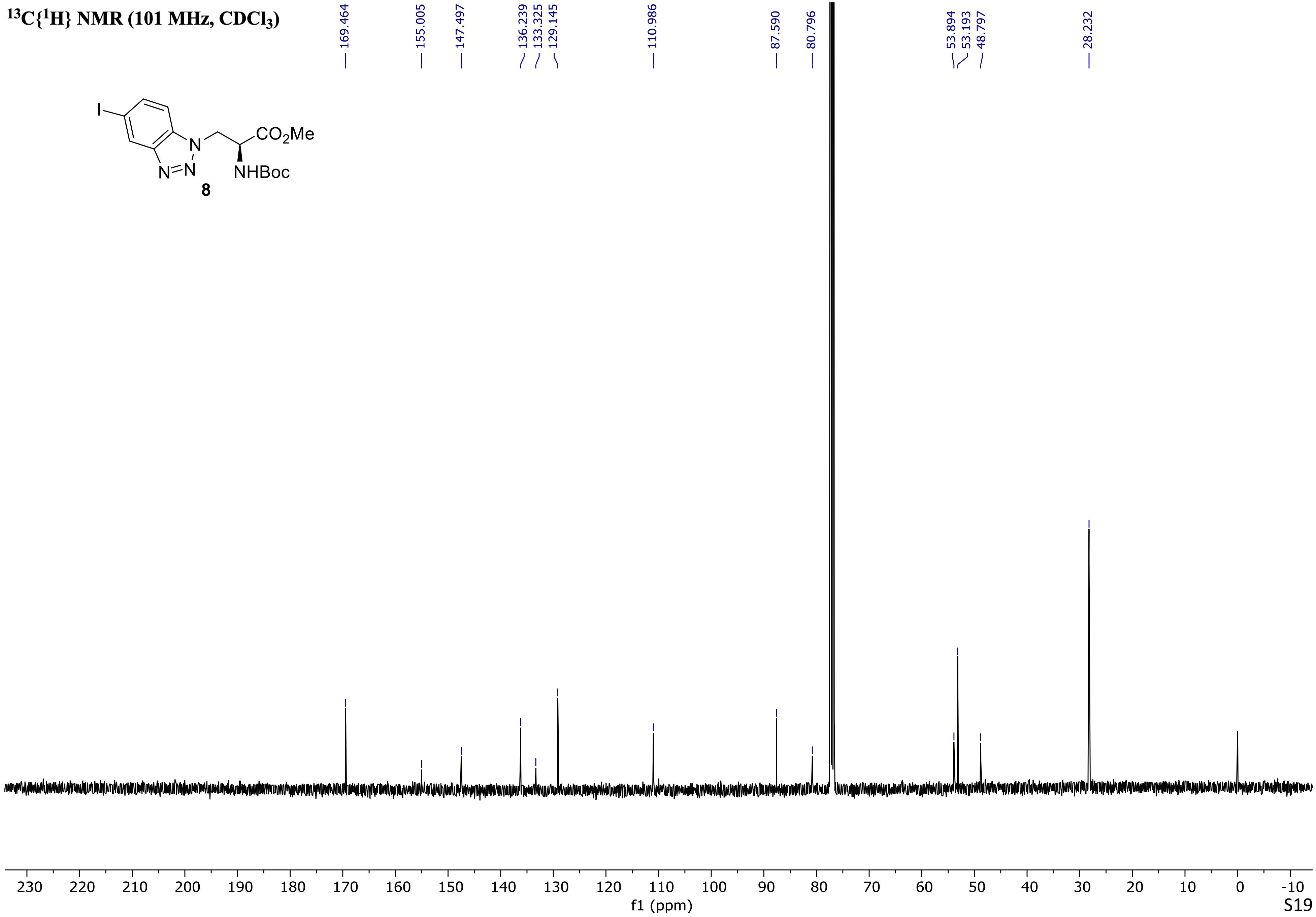

^{1}H NMR (400 MHz, $CDCl_3$)

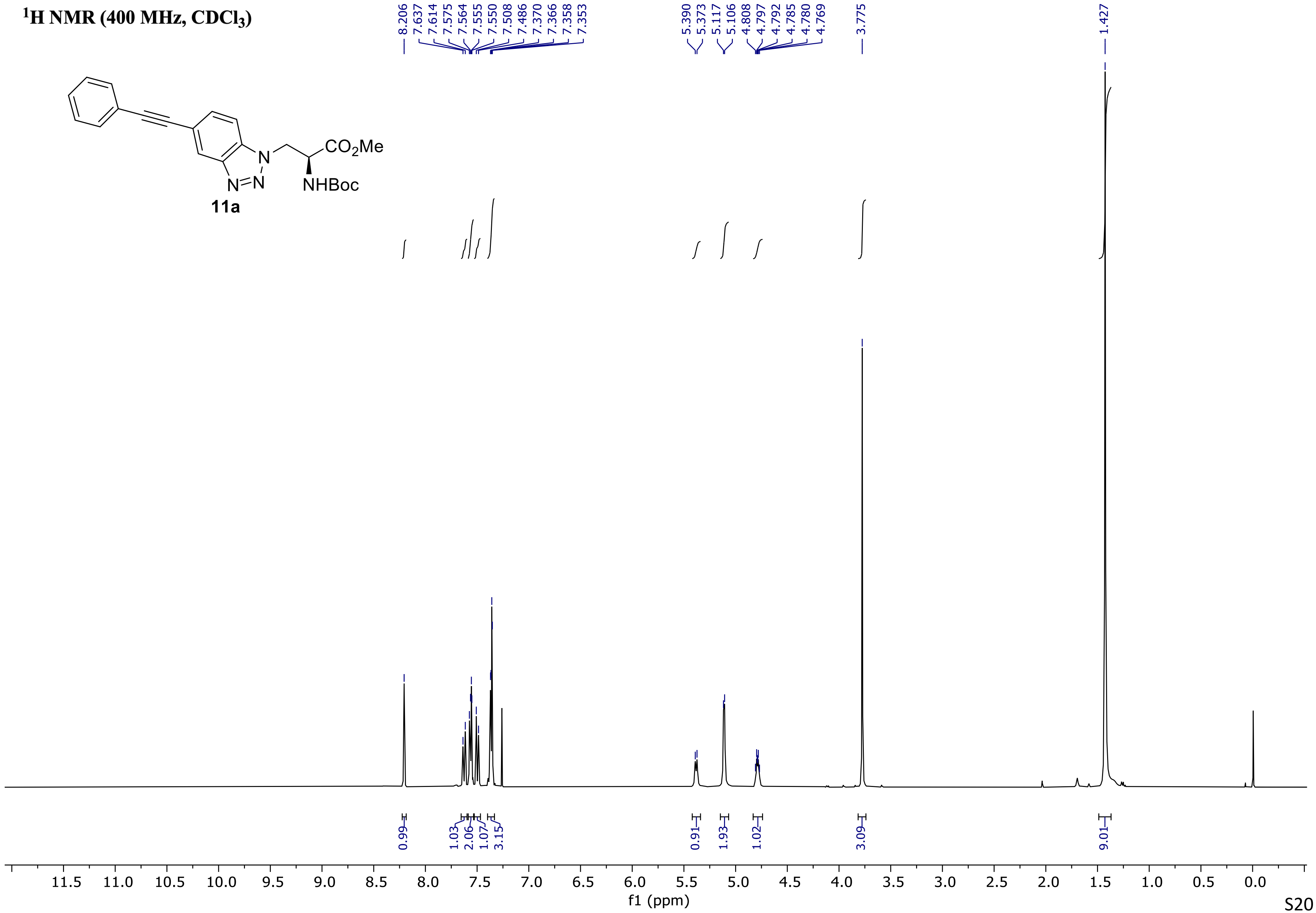

$^{13}C\{^1H\}$ NMR (101 MHz, $CDCl_3$)

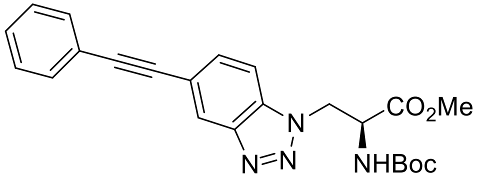

11a

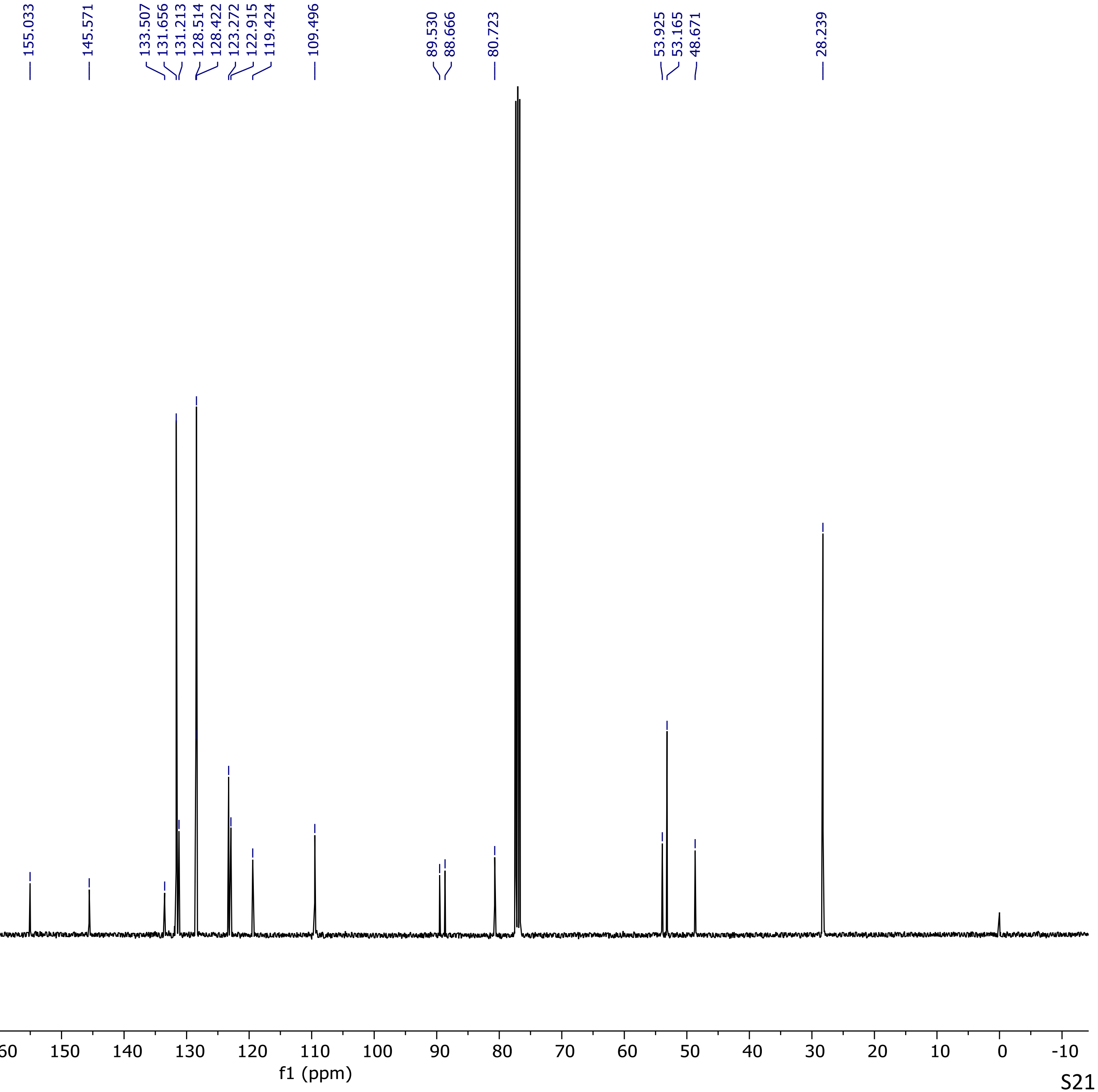

^{1}H NMR (400 MHz, $CDCl_3$)

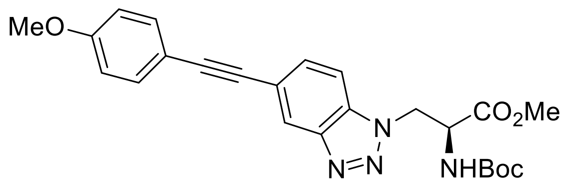

11b

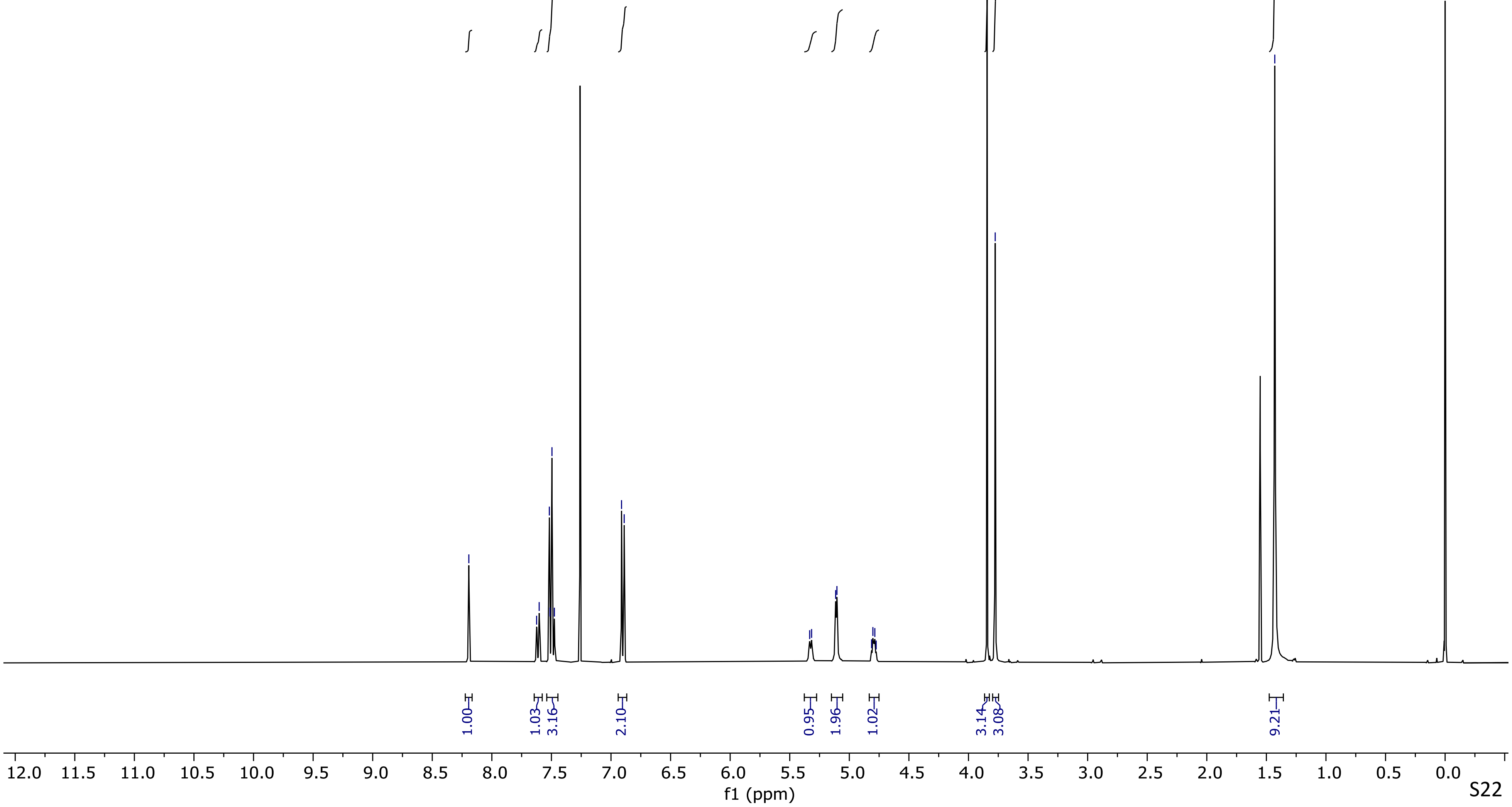

$^{13}C\{^{1}H\}$ NMR (101 MHz, $CDCl_3$)

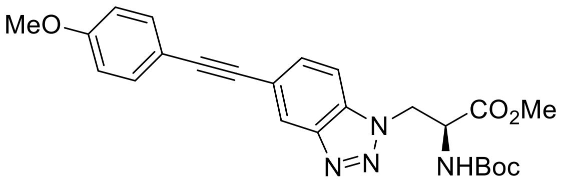

11b

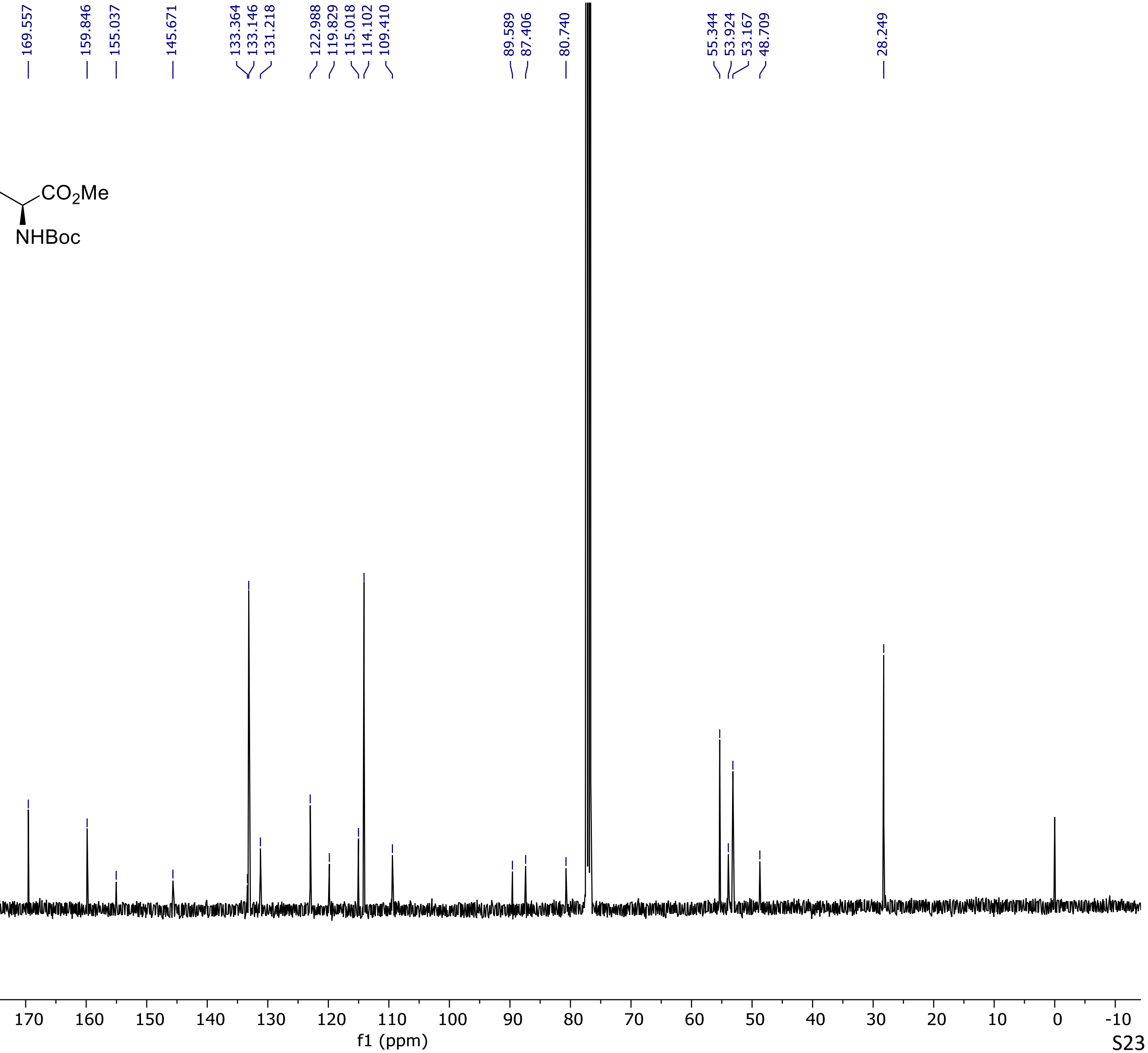

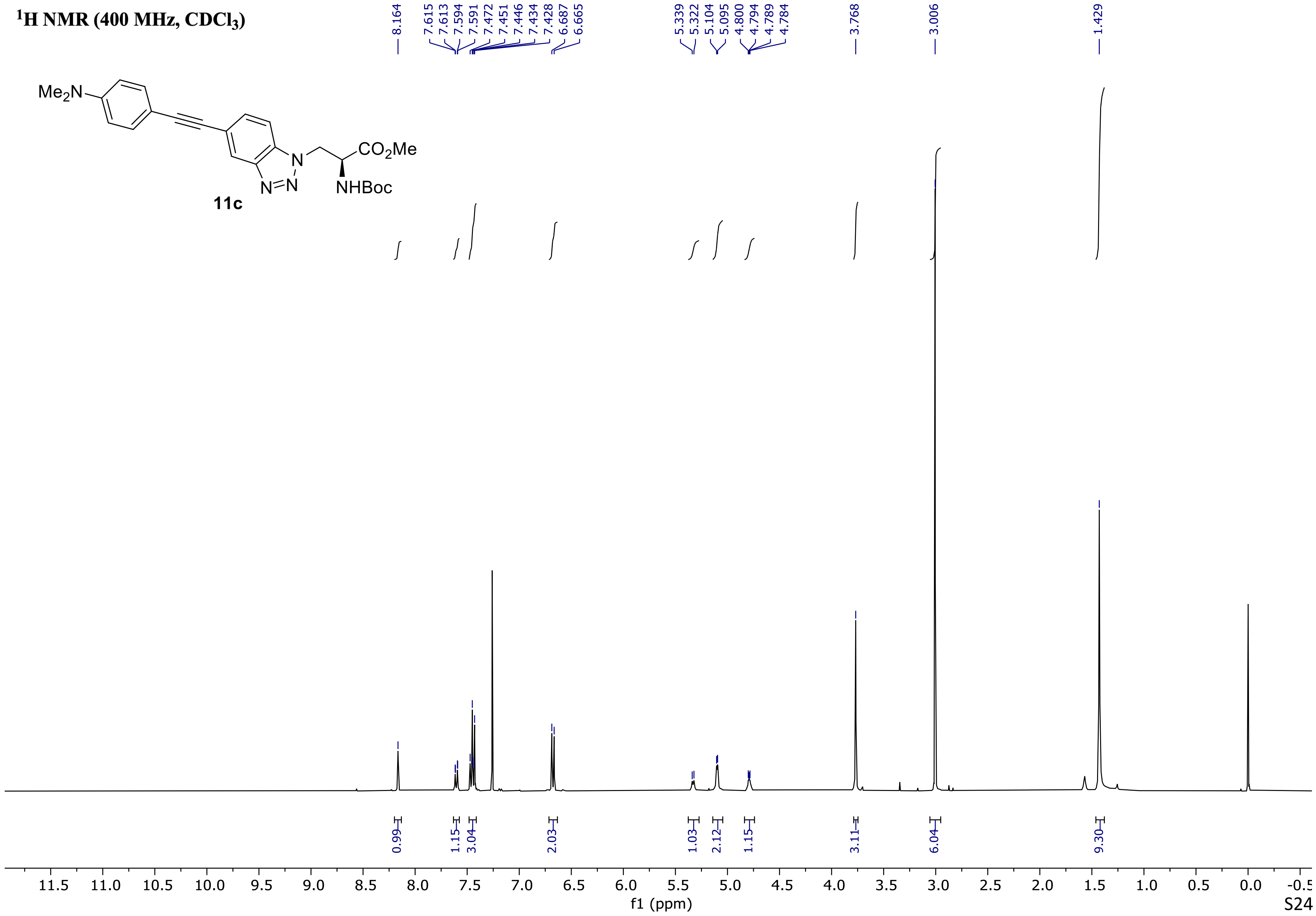

^{1}H NMR (400 MHz, $CDCl_3$)
Me2N
CO2Me
NHBoc
11c
8.164
7.615
7.613
7.594
7.591
7.472
7.451
7.446
7.434
7.428
6.687
6.665
5.339
5.322
5.104
5.095
4.800
4.794
4.789
4.784
3.768
3.006
1.429
0.99
1.15
3.04
2.03
1.03
2.12
1.15
3.11
6.04
9.30
11.5
11.0
10.5
10.0
9.5
9.0
8.5
8.0
7.5
7.0
6.5
6.0
5.5
5.0
4.5
4.0
3.5
3.0
2.5
2.0
1.5
1.0
0.5
0.0
-0.5
f1 (ppm)

$^{13}C\{^{1}H\}$ NMR (101 MHz, $CDCl_3$)

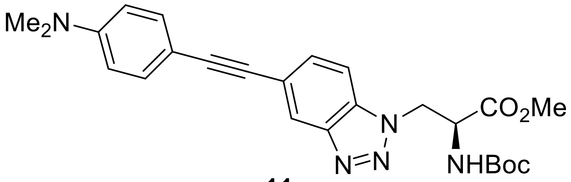

11c

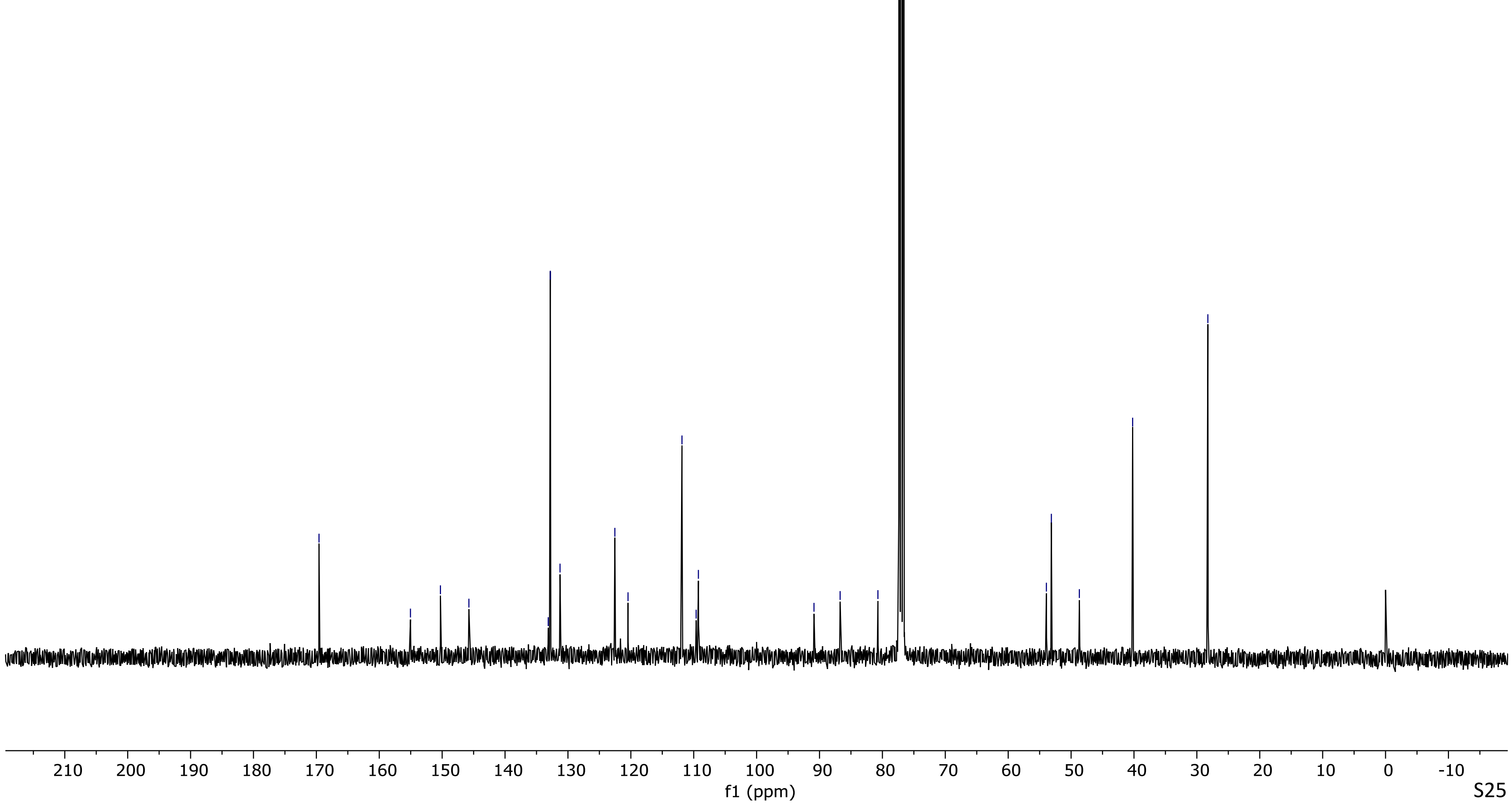

^{1}H NMR (500 MHz, $CDCl_3$)

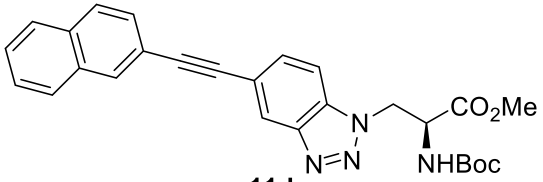

11d

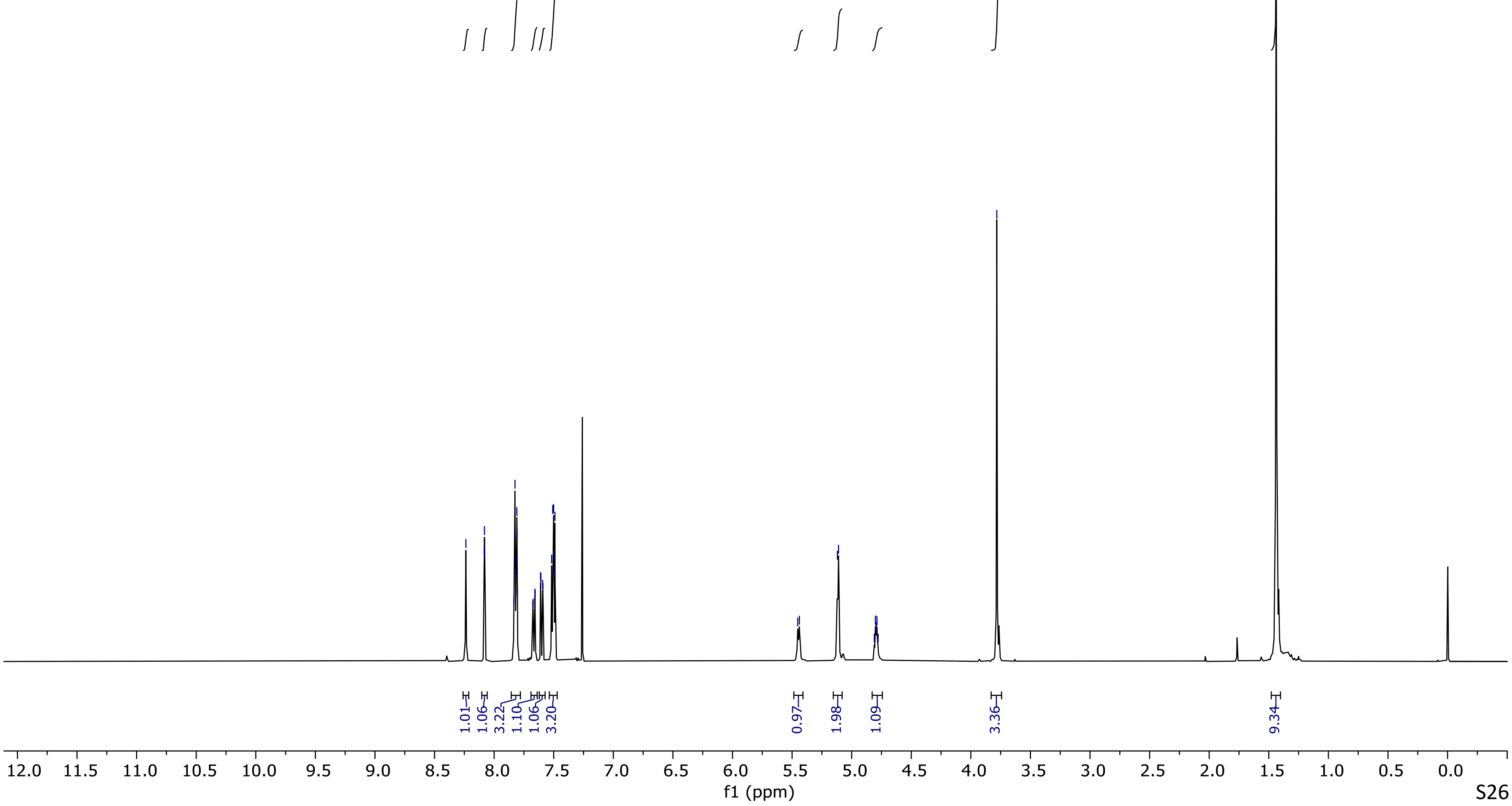

$^{13}C\{^{1}H\}$ NMR (126 MHz, $CDCl_3$)

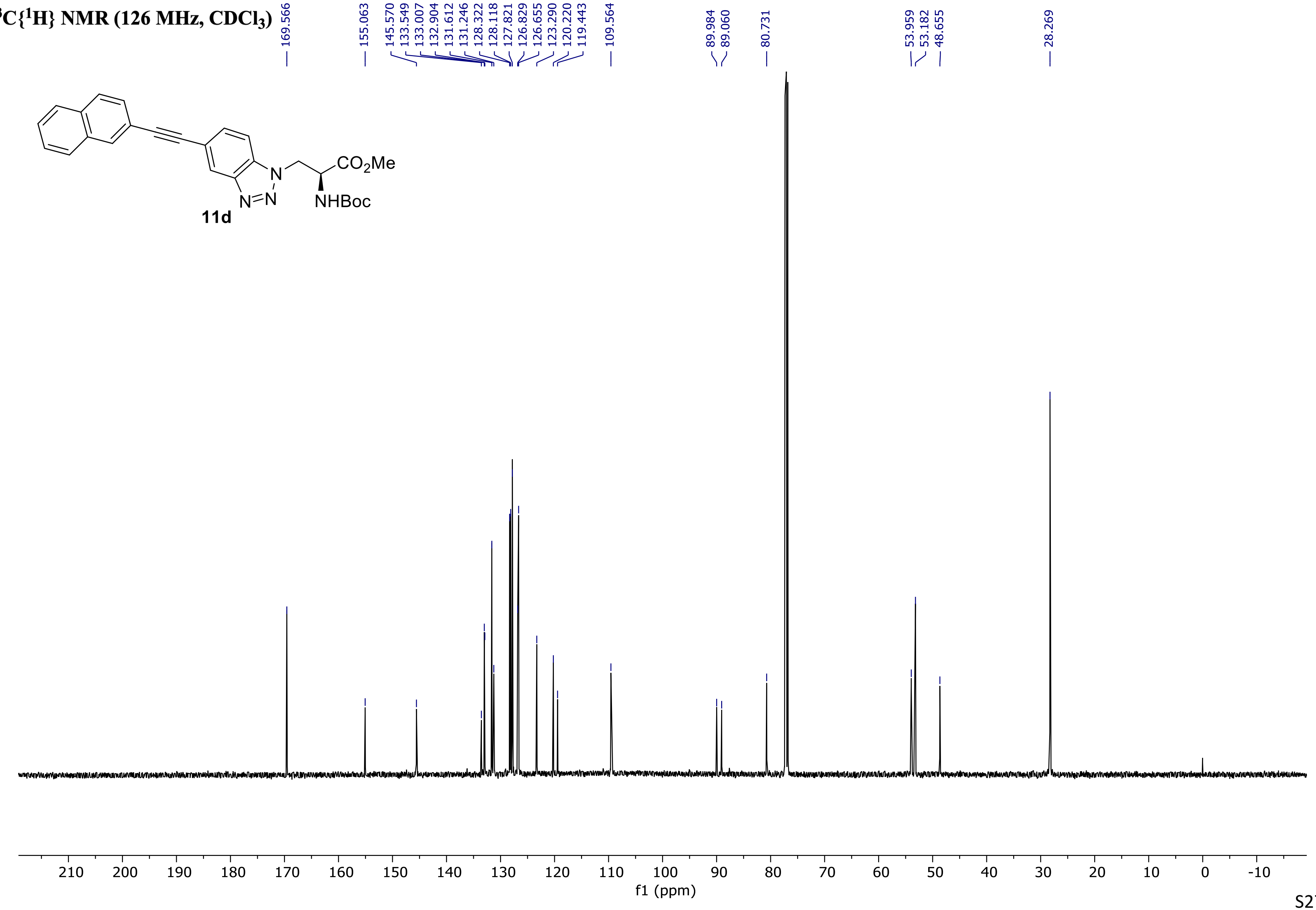

11d

^1H NMR (400 MHz, $CDCl_3$)

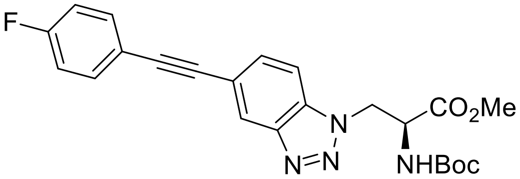

11e

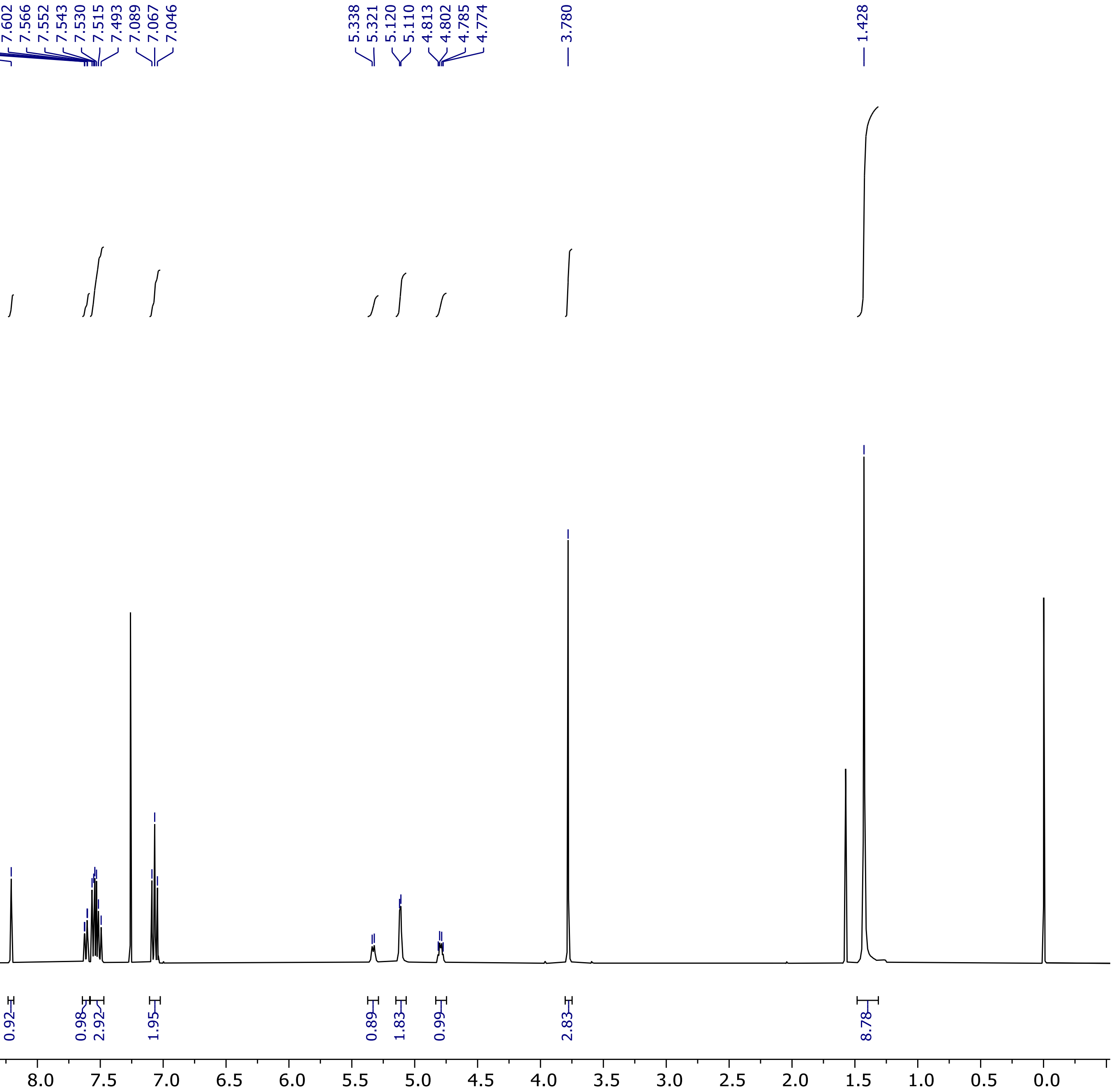

$^{13}C\{^1H\}$ NMR (101 MHz, $CDCl_3$)

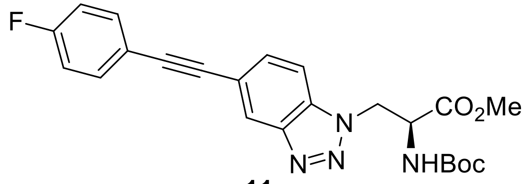

11e

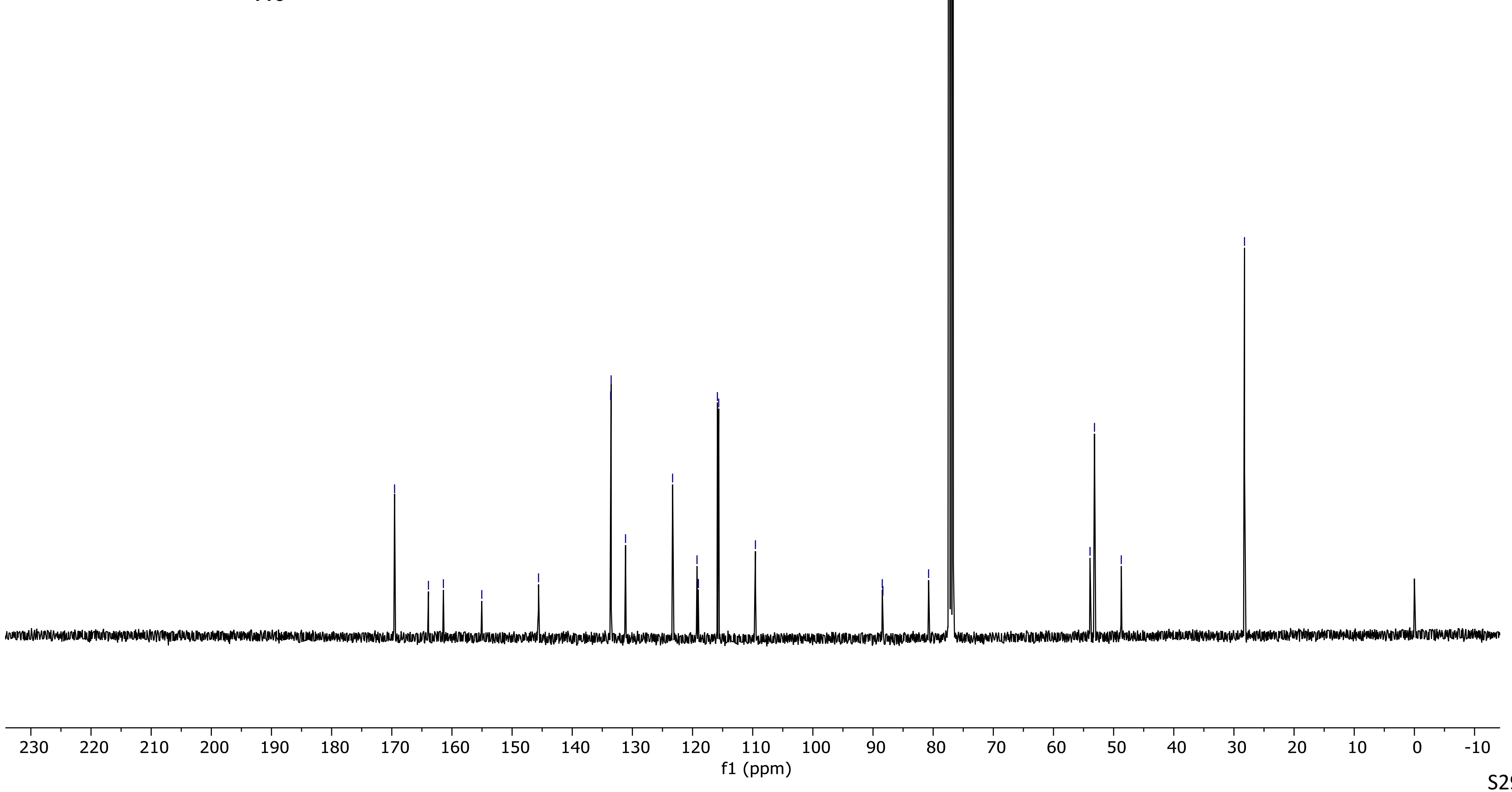

^{1}H NMR (500 MHz, $CDCl_3$)

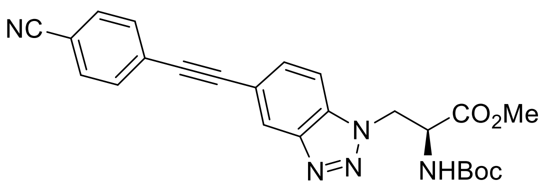

11f

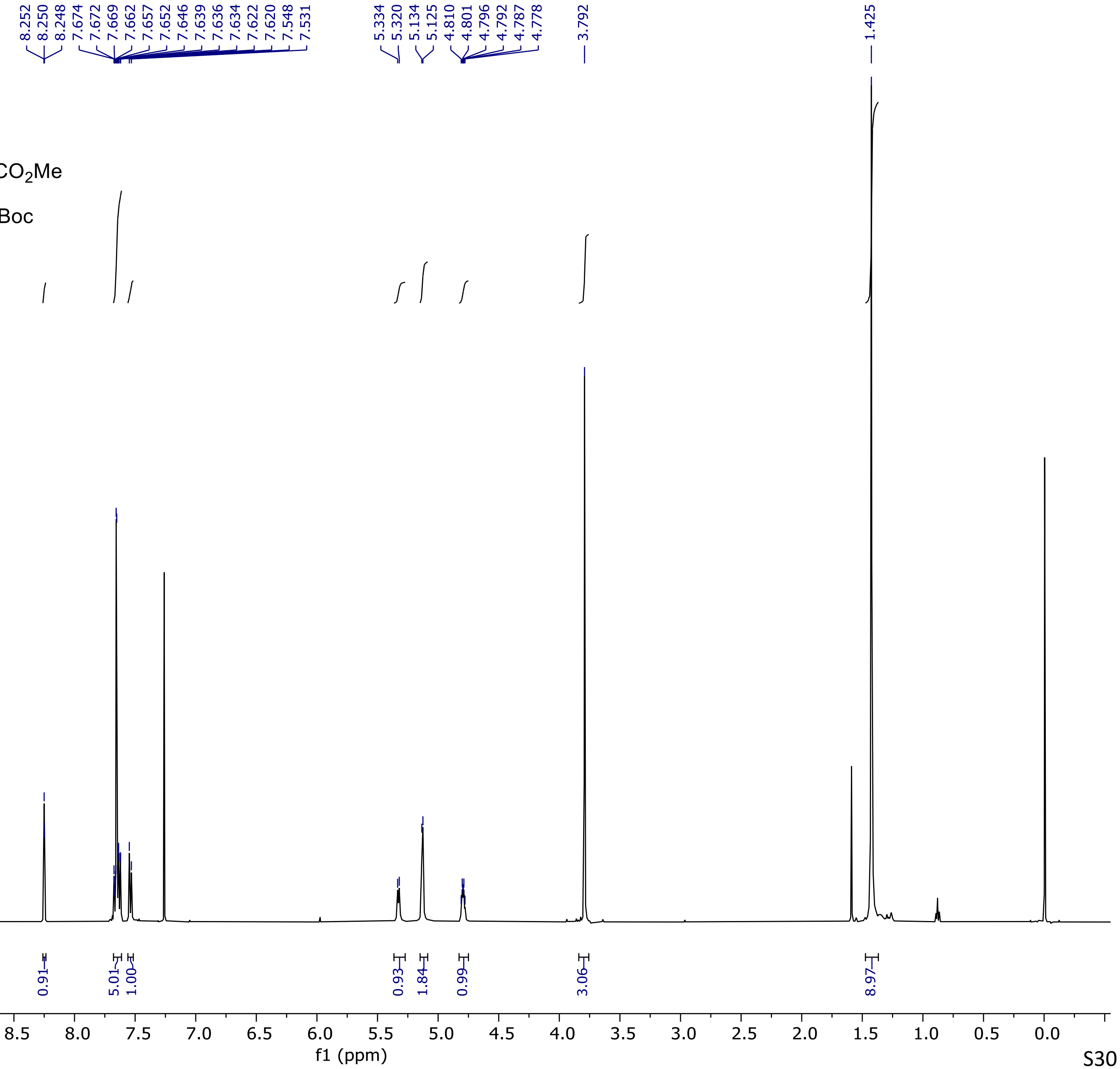

$^{13}C\{^{1}H\}$ NMR (126 MHz, $CDCl_3$)

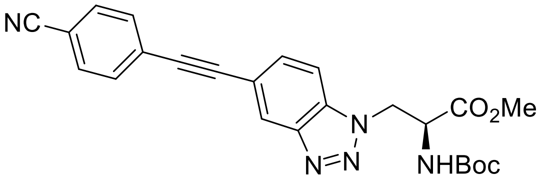

11f

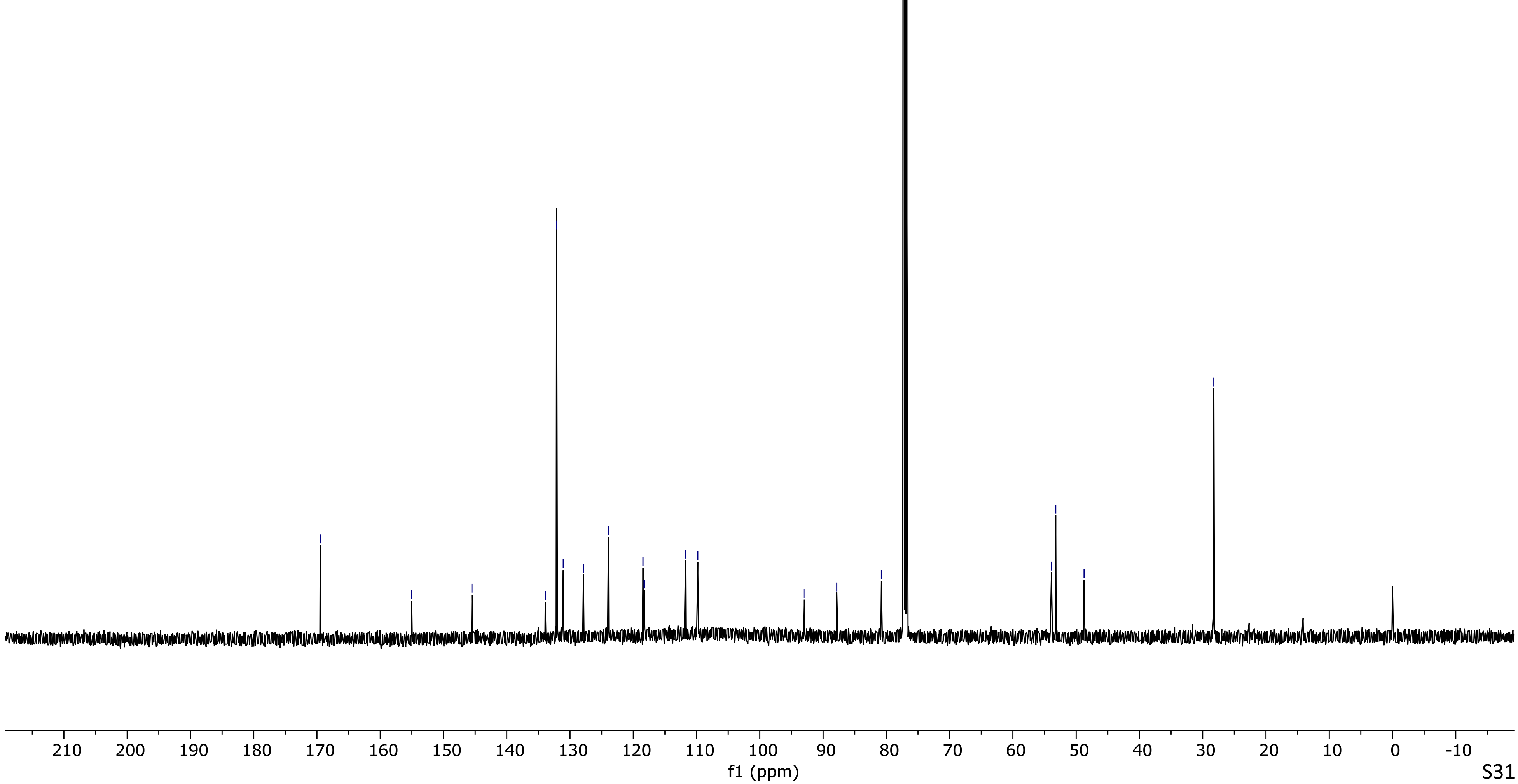

^{1}H NMR (400 MHz, CD_3OD)

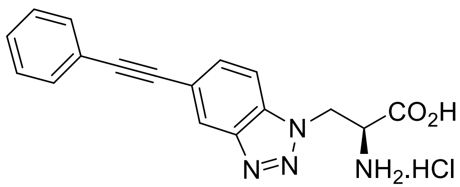

12a

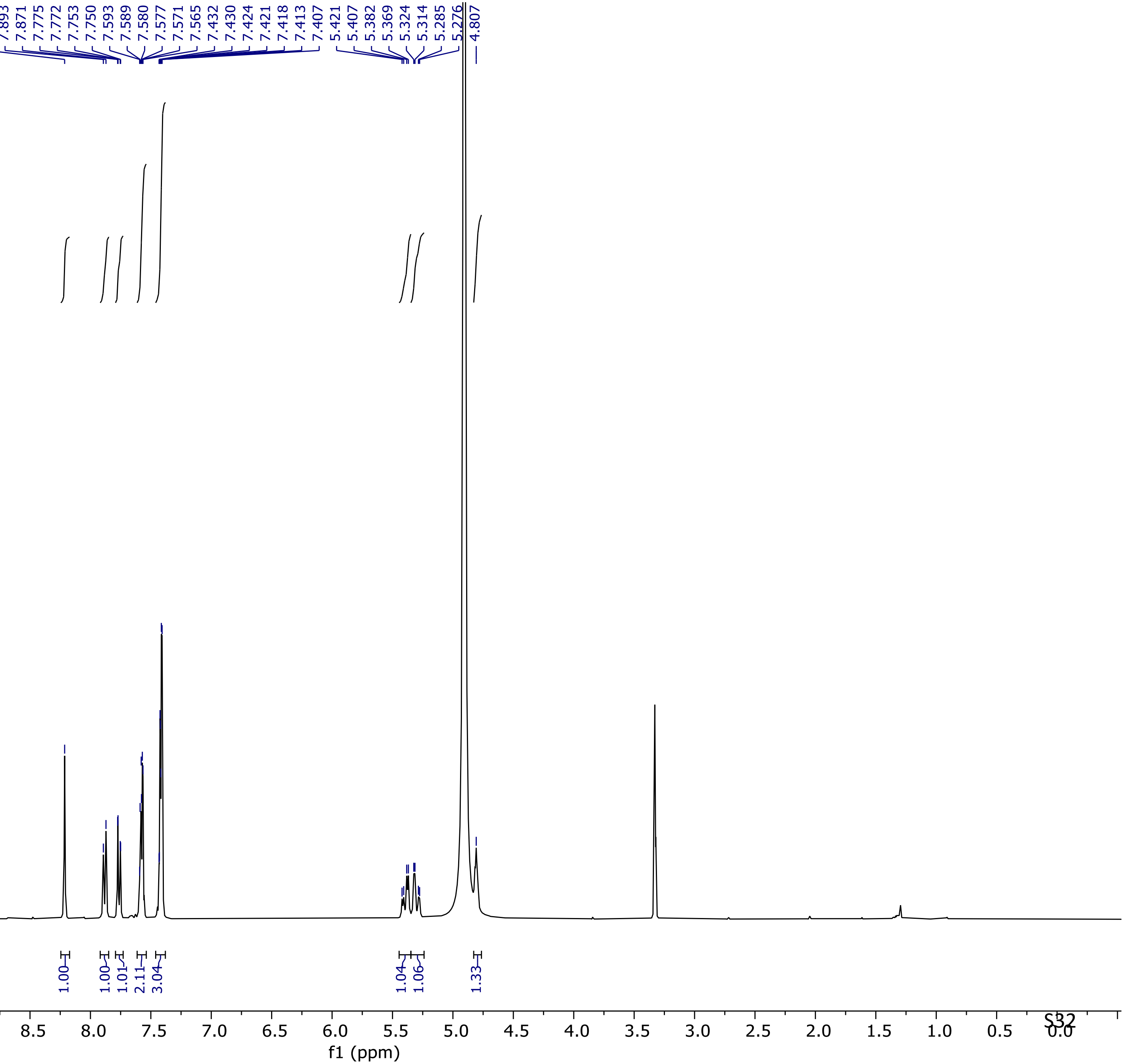

$^{13}C\{^1H\}$ NMR (101 MHz, CD_3OD)

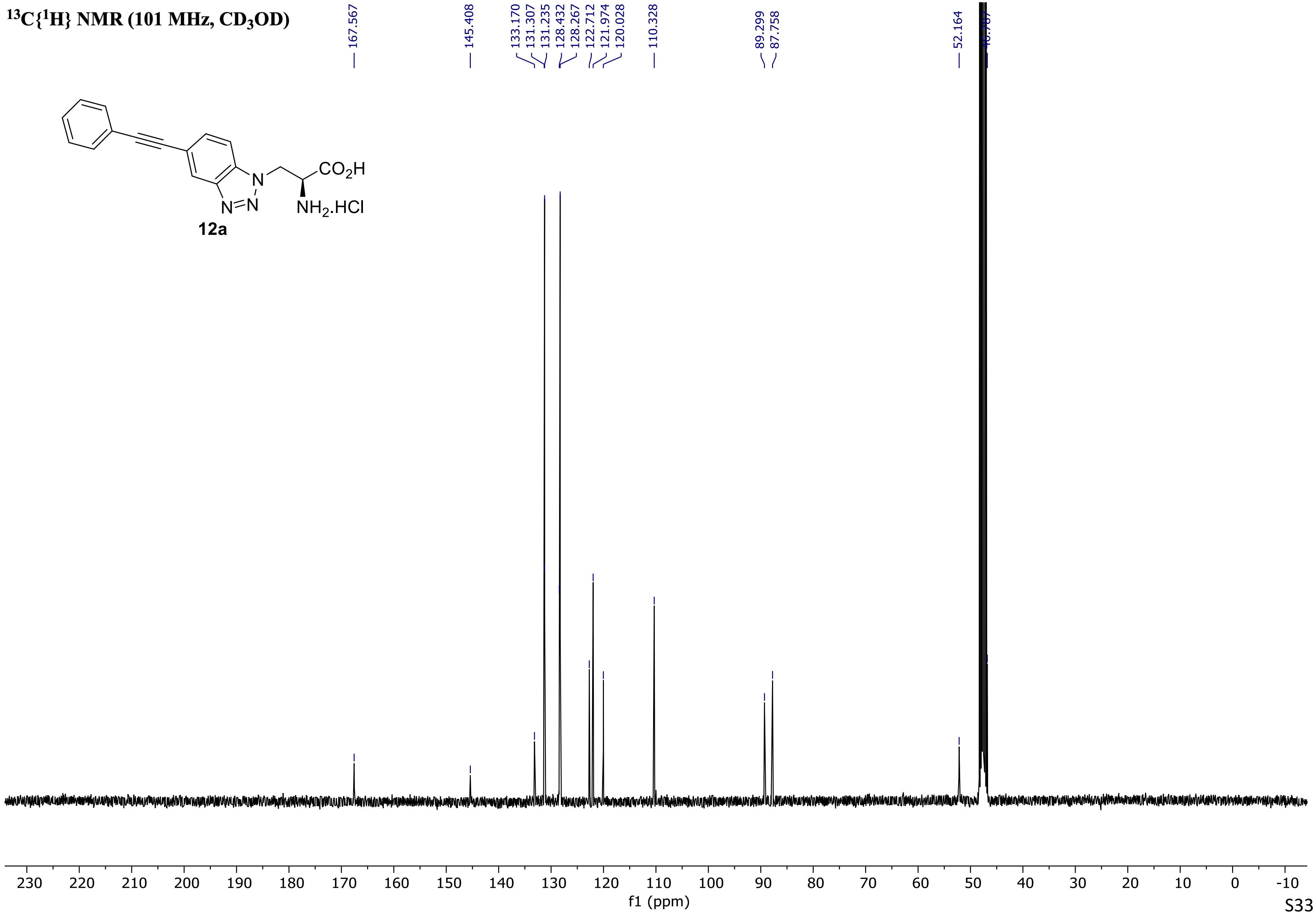

^{1}H NMR (400 MHz, DMSO-d_6)

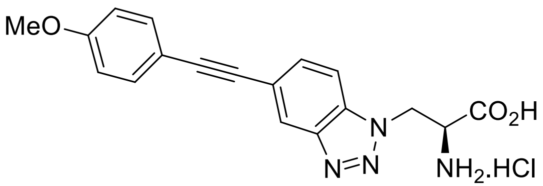

12b

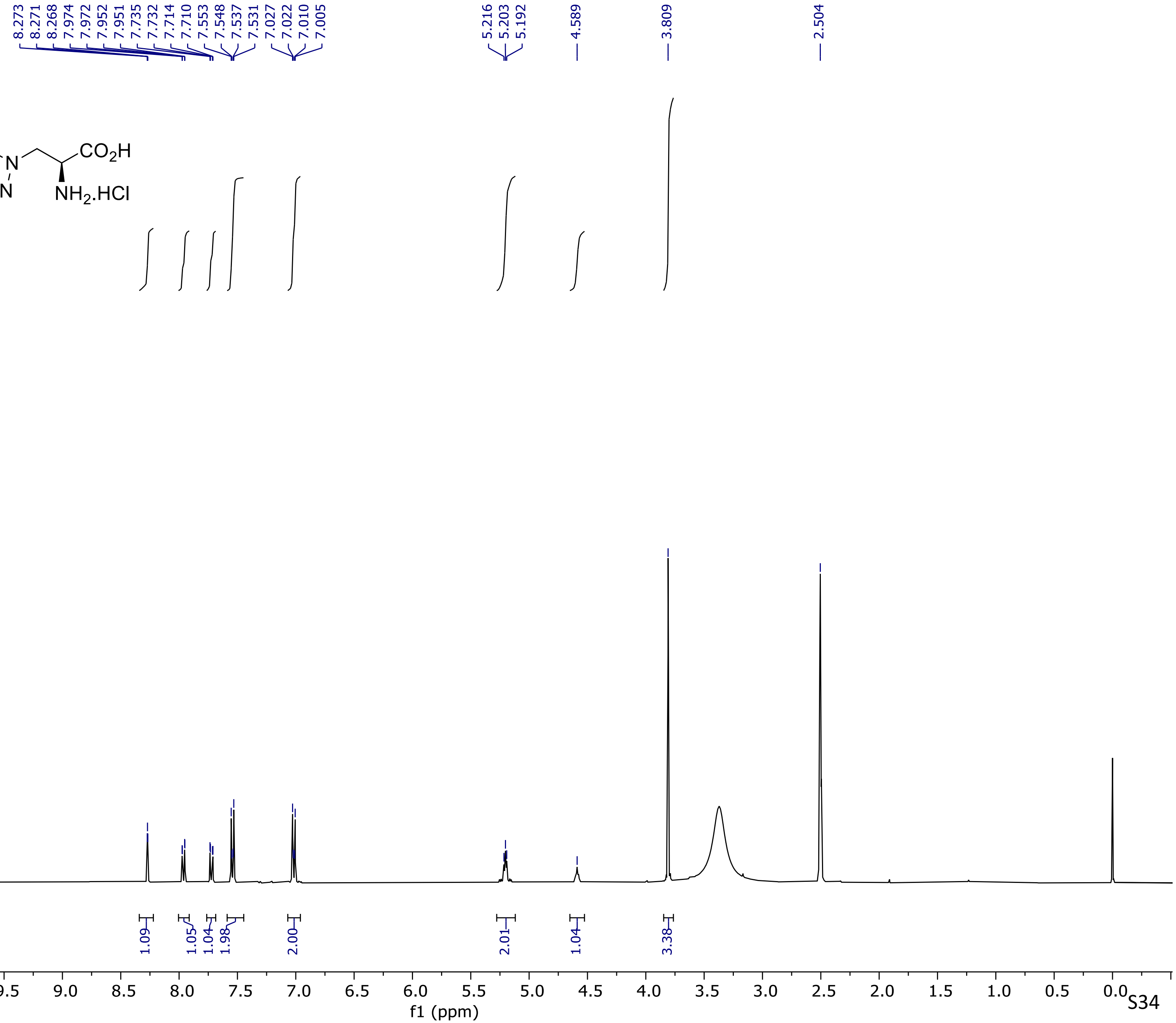

$^{13}C\{^{1}H\}$ NMR (126 MHz, DMSO-d_6)

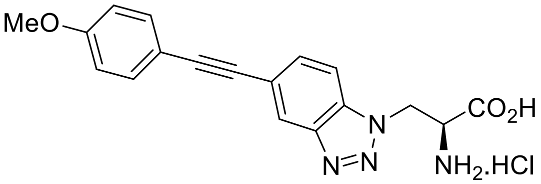

12b

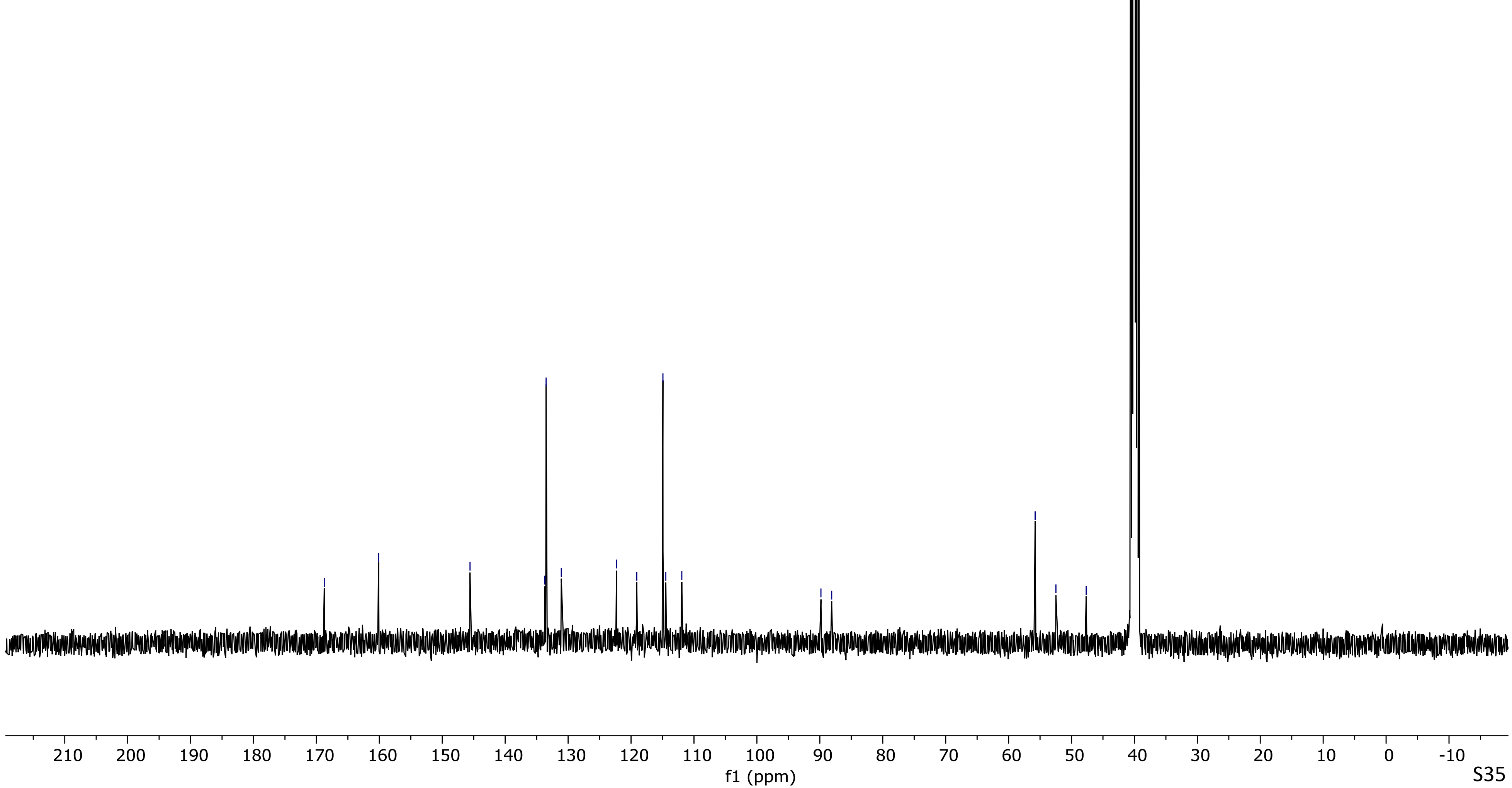

^{1}H NMR (400 MHz, CD_3OD)

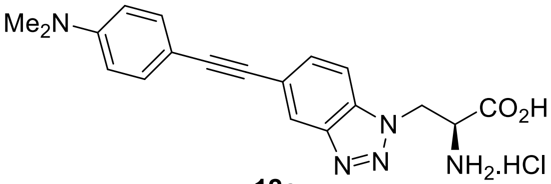

12c

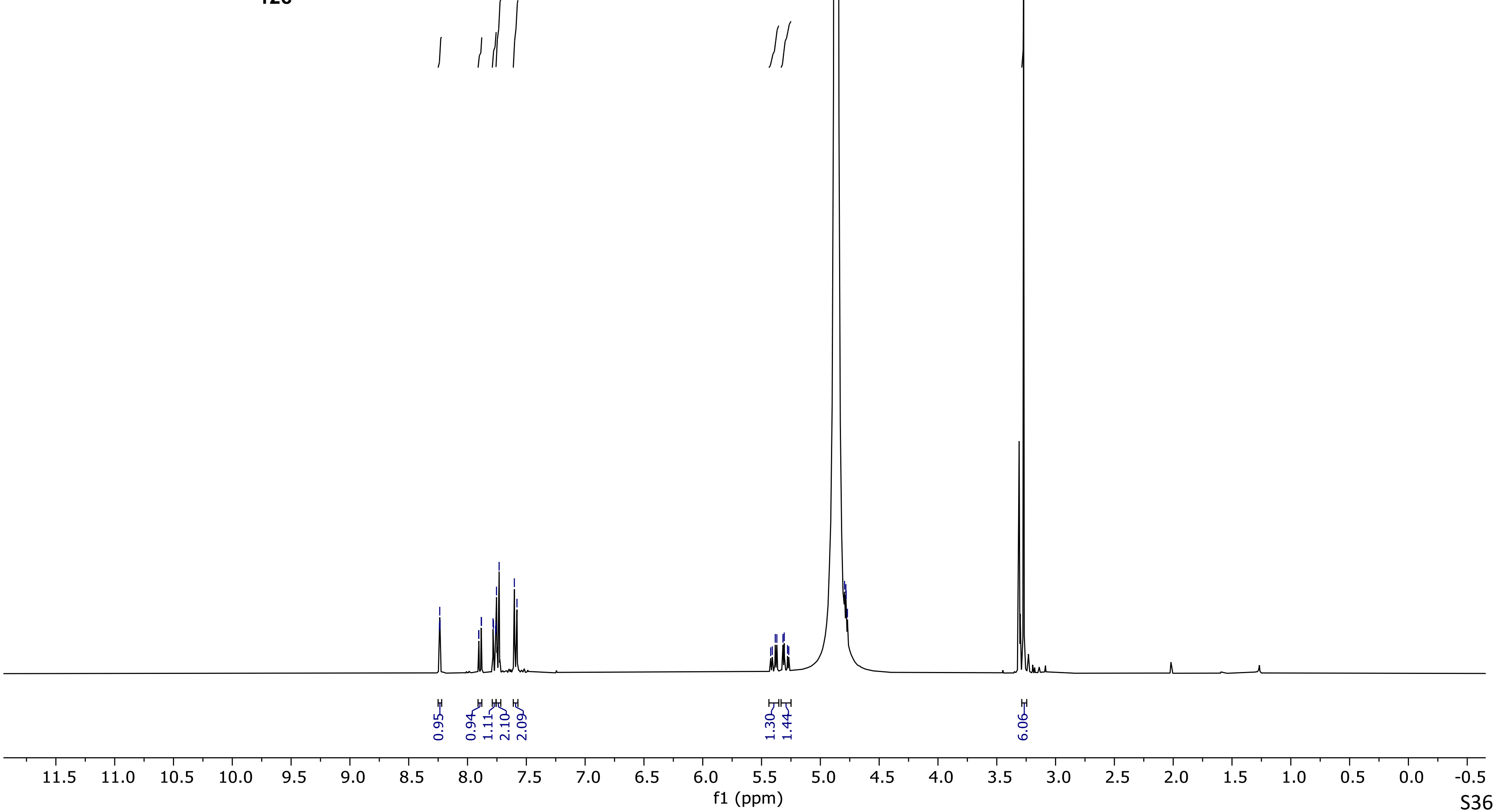

$^{13}C\{^{1}H\}$ NMR (101 MHz, CD_3OD)

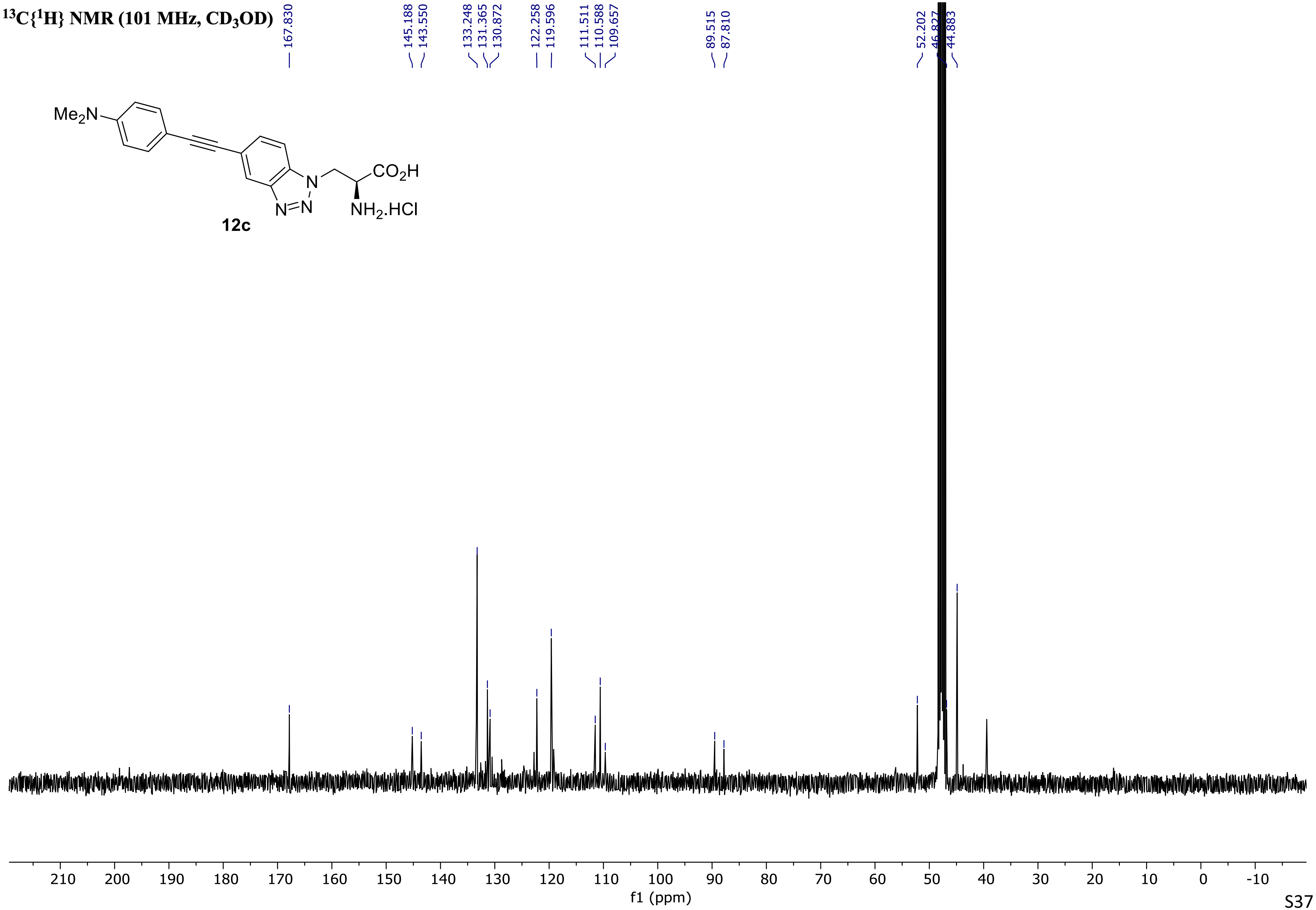

^{1}H NMR (400 MHz, CD_3OD)

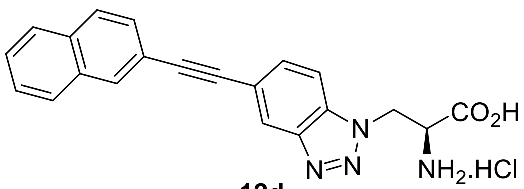

12d

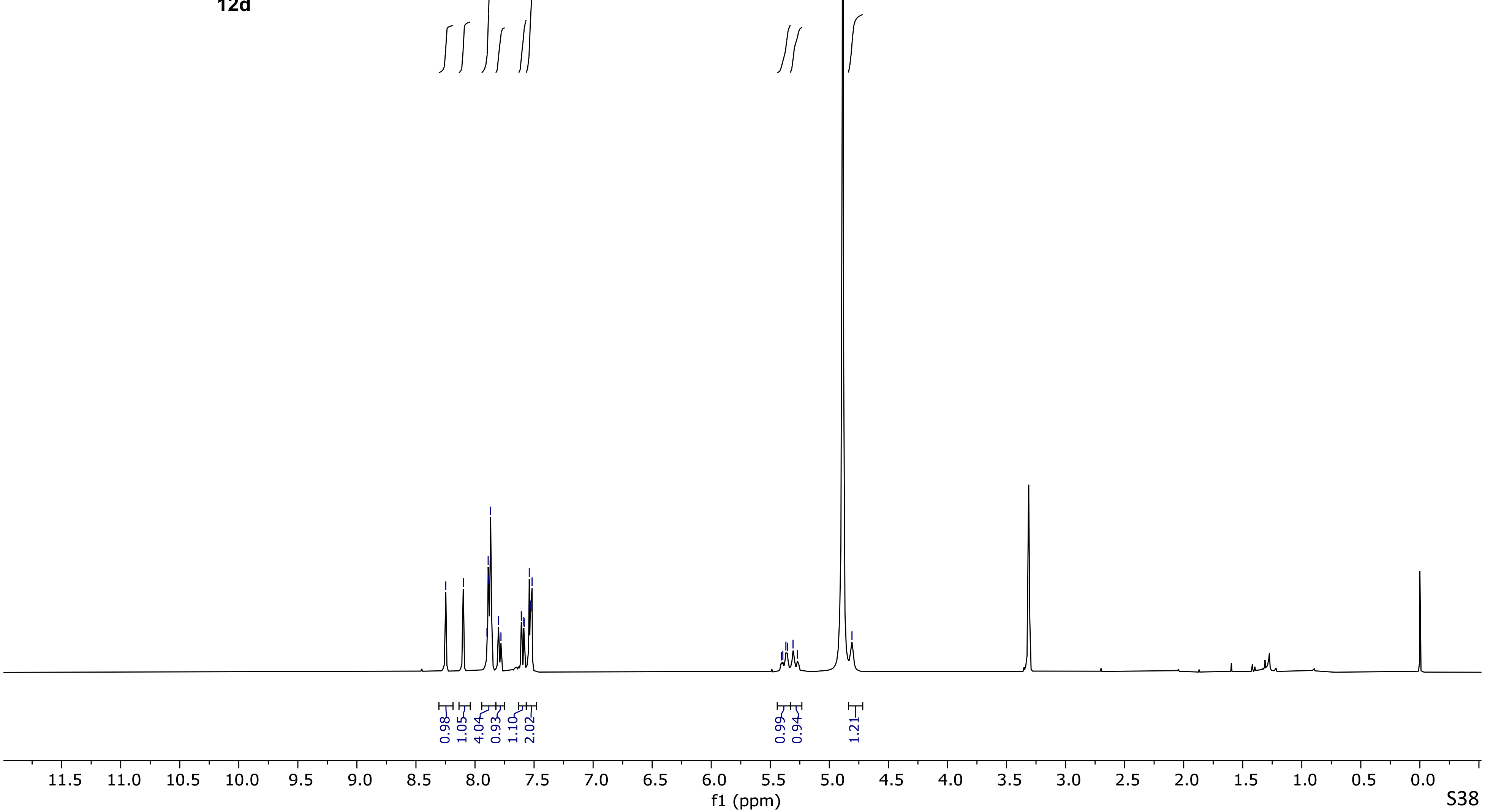

$^{13}C\{^{1}H\}$ NMR (101 MHz, CD_3OD)

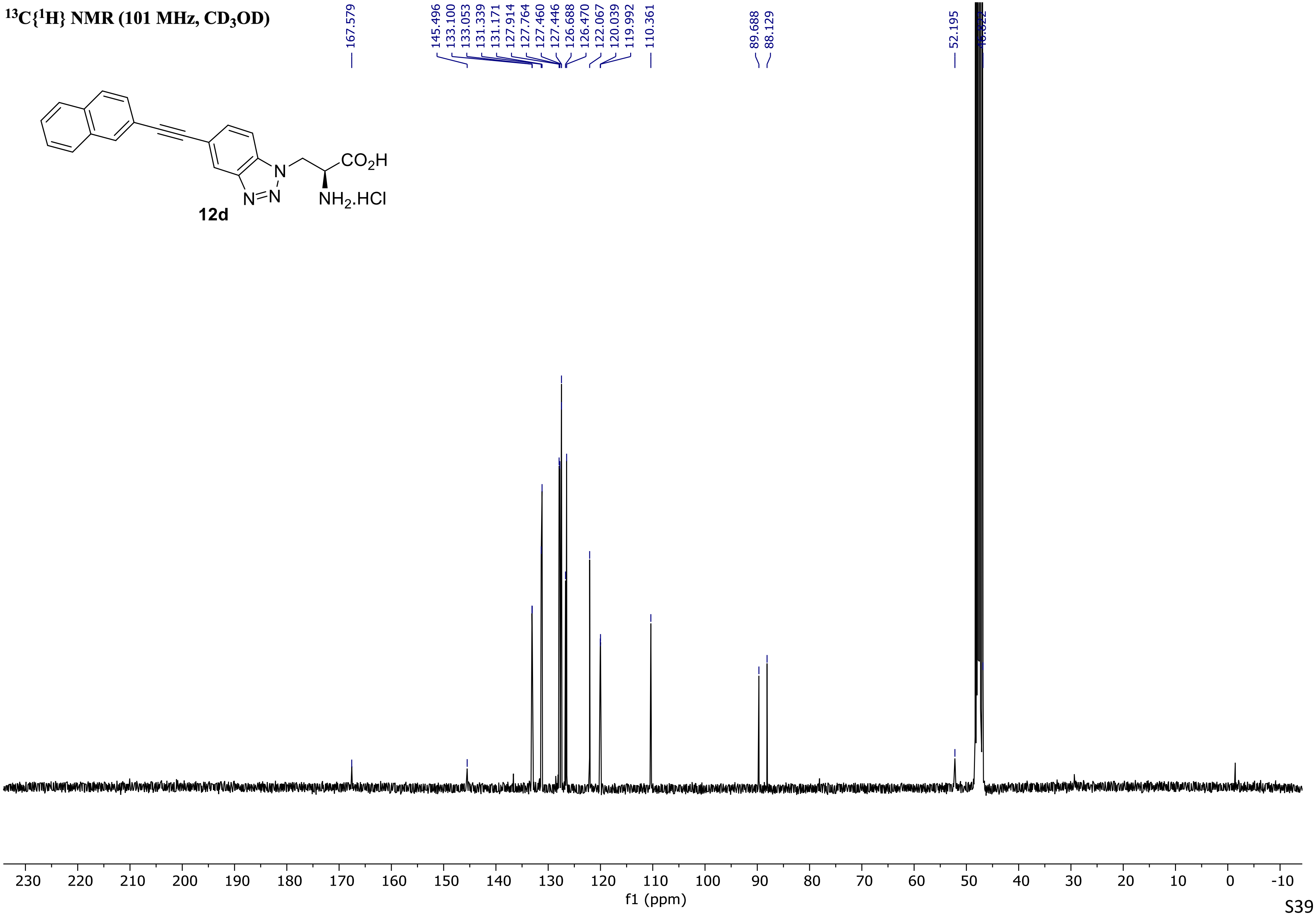

^{1}H NMR (400 MHz, CD_3OD)

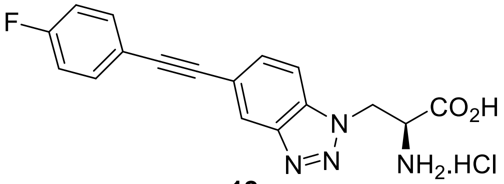

12e

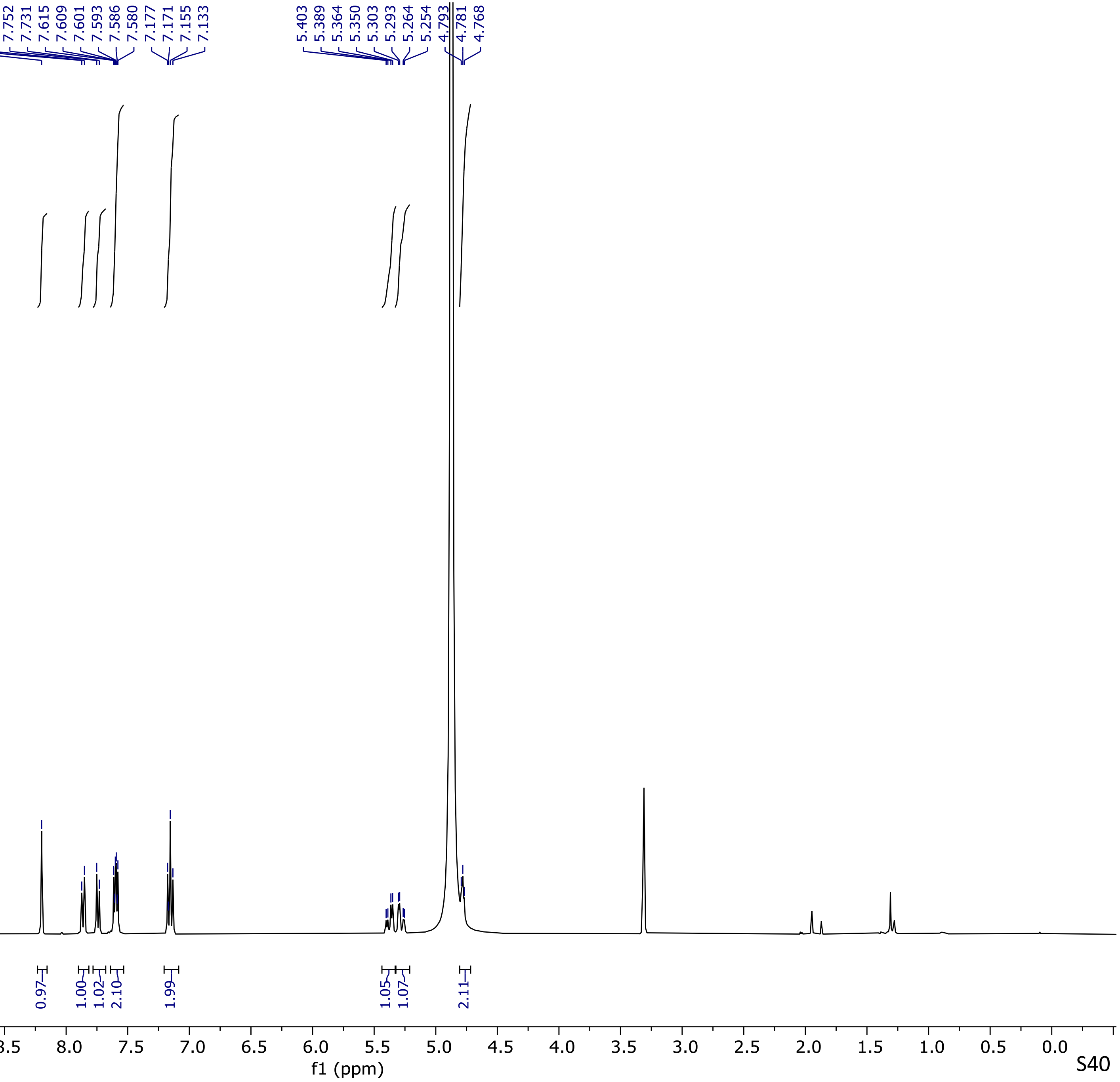

$^{13}C\{^1H\}$ NMR (101 MHz, CD_3OD)

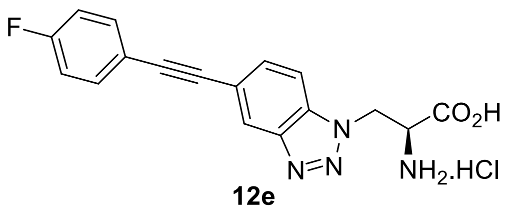

12e

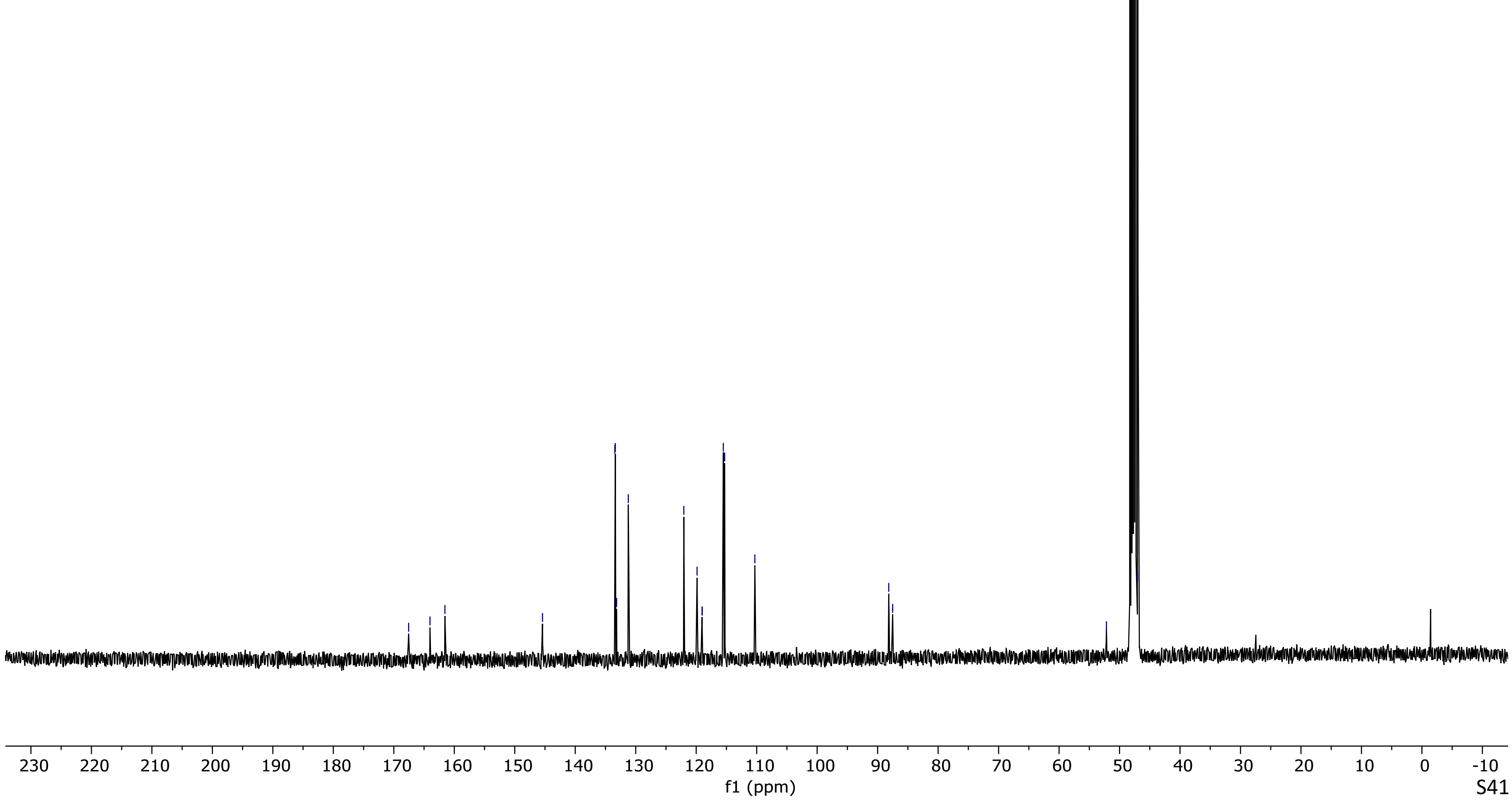

^{1}H NMR (500 MHz, DMSO-d_6)

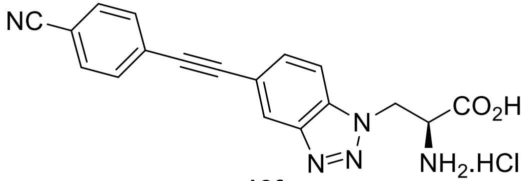

12f

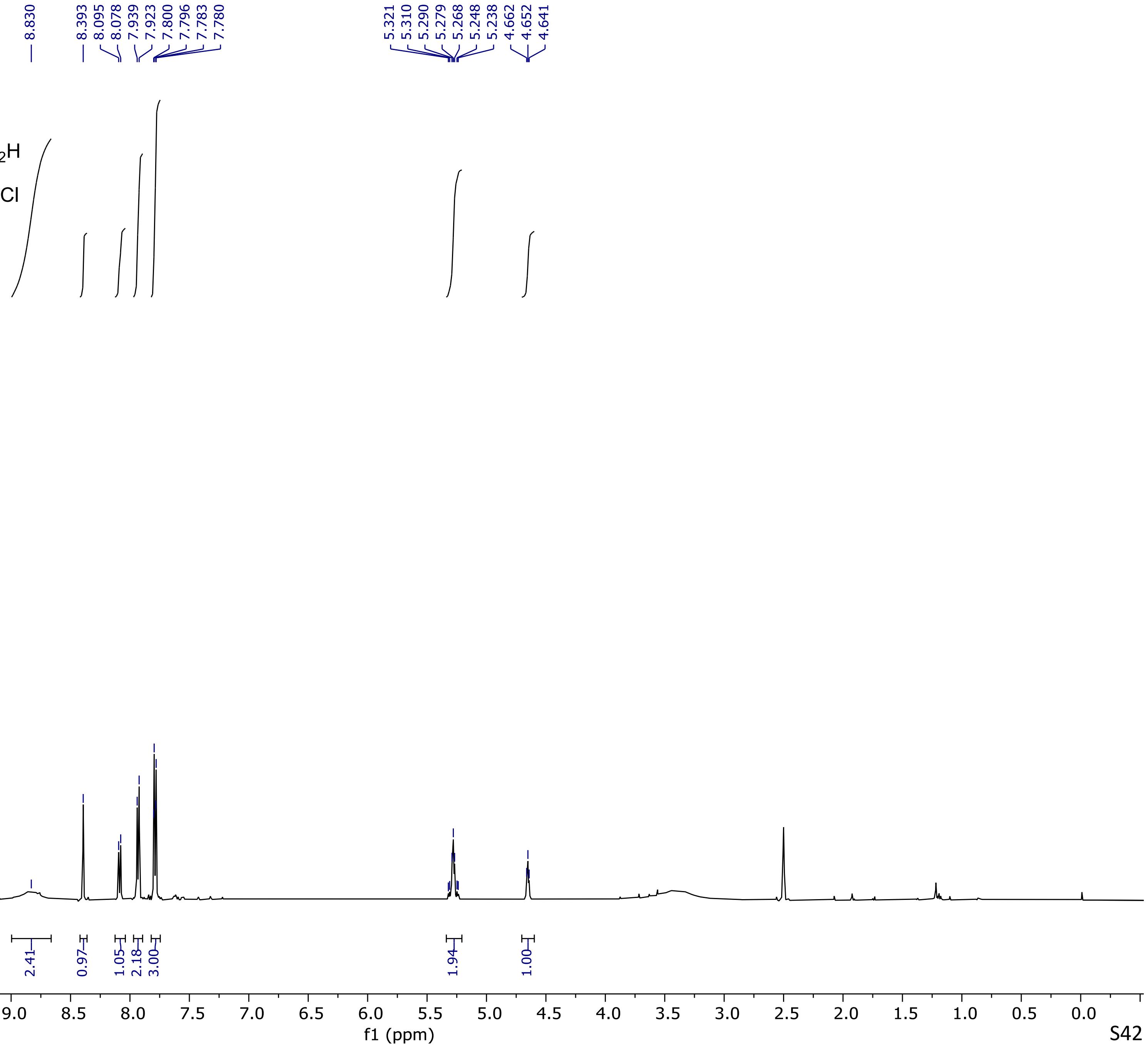

$^{13}C\{^{1}H\}$ NMR (126 MHz, DMSO-d_6)

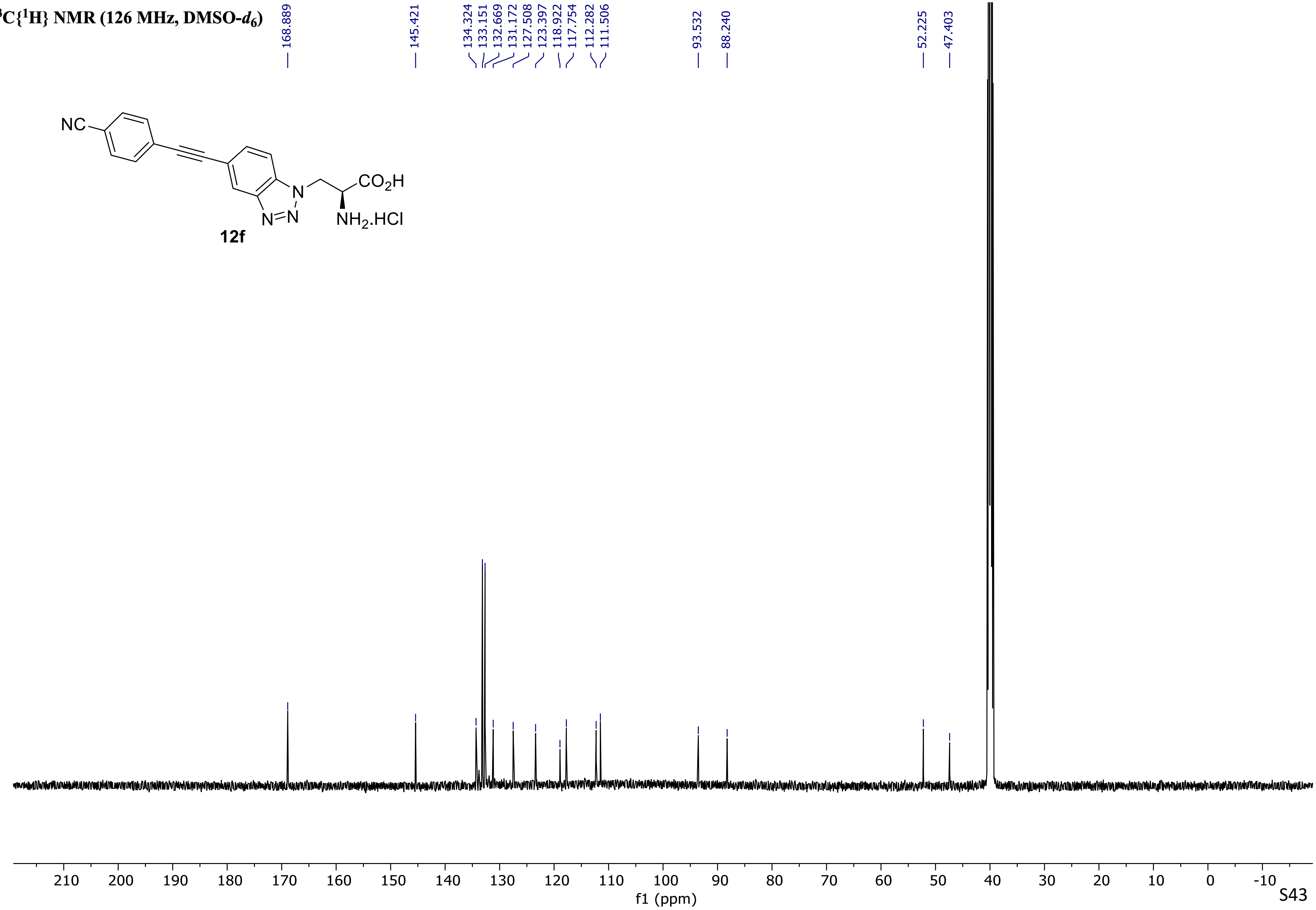

^{1}H NMR (400 MHz, $CDCl_3$)

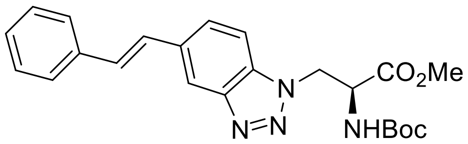

13a

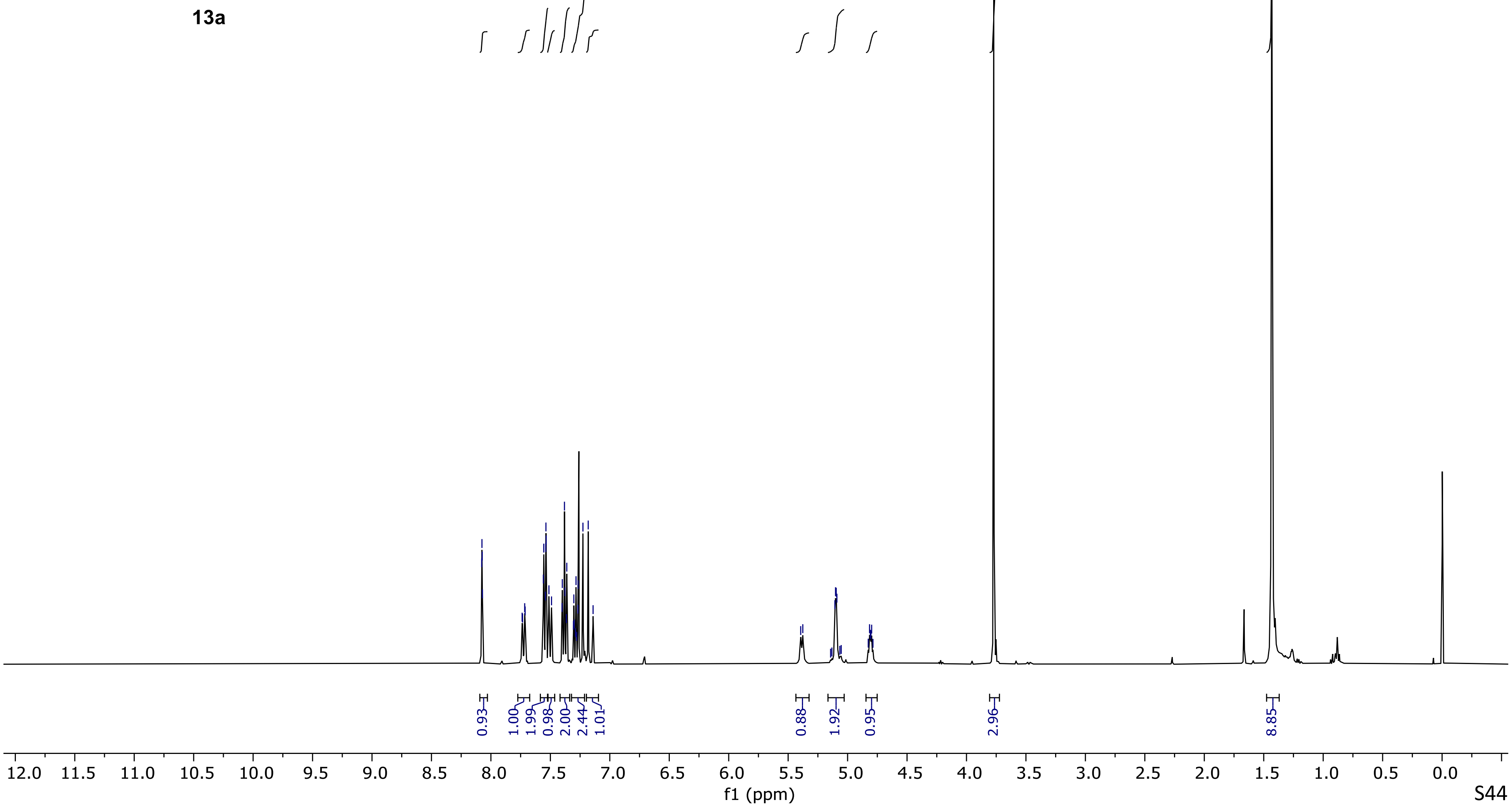

$^{13}C\{^1H\}$ NMR (101 MHz, $CDCl_3$)

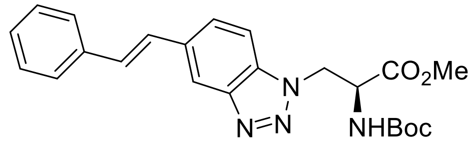

13a

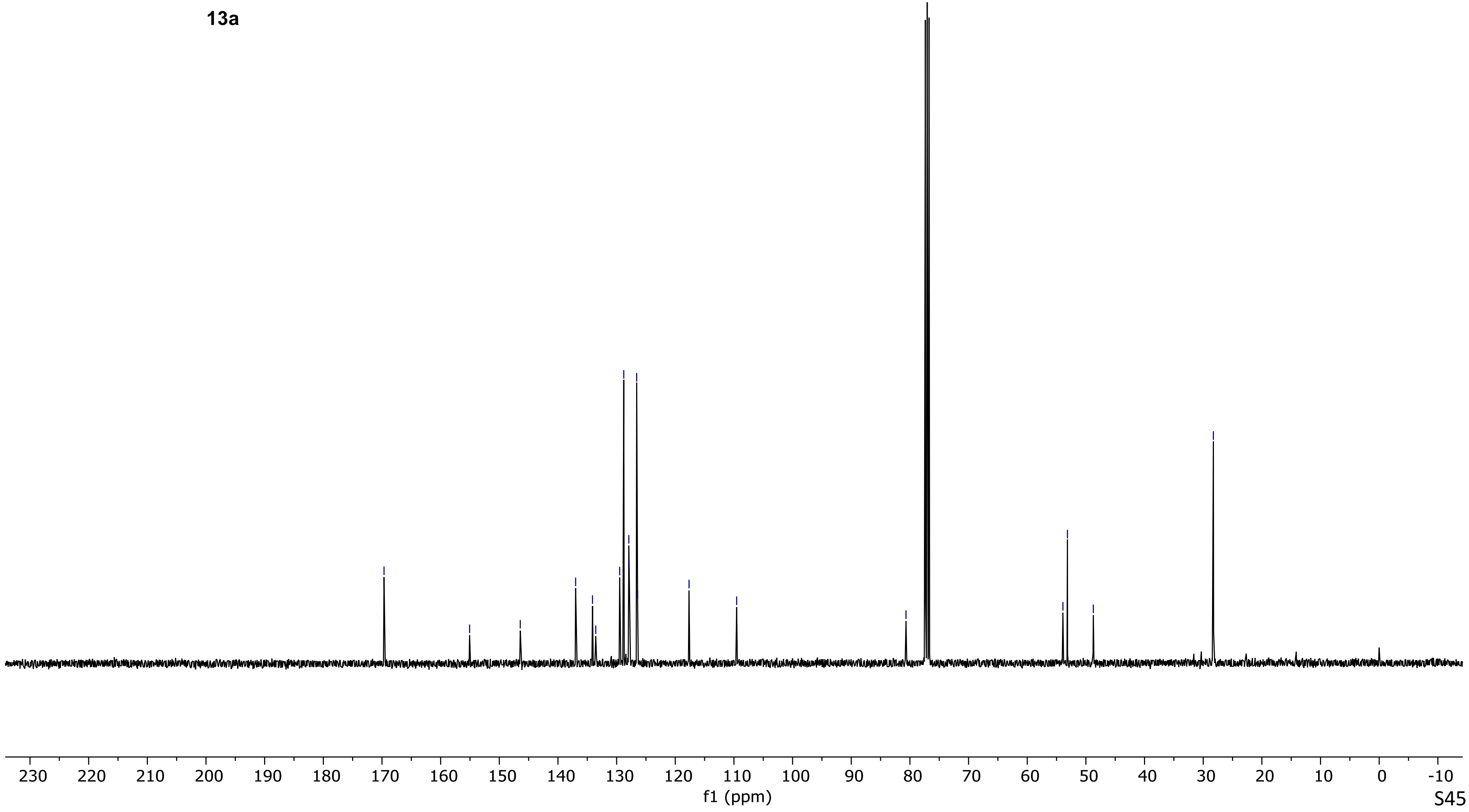

^{1}H NMR (500 MHz, $CDCl_3$)

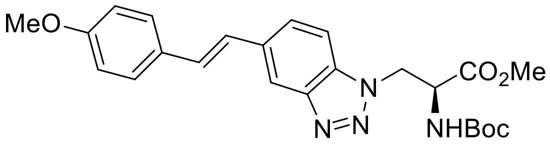

13b

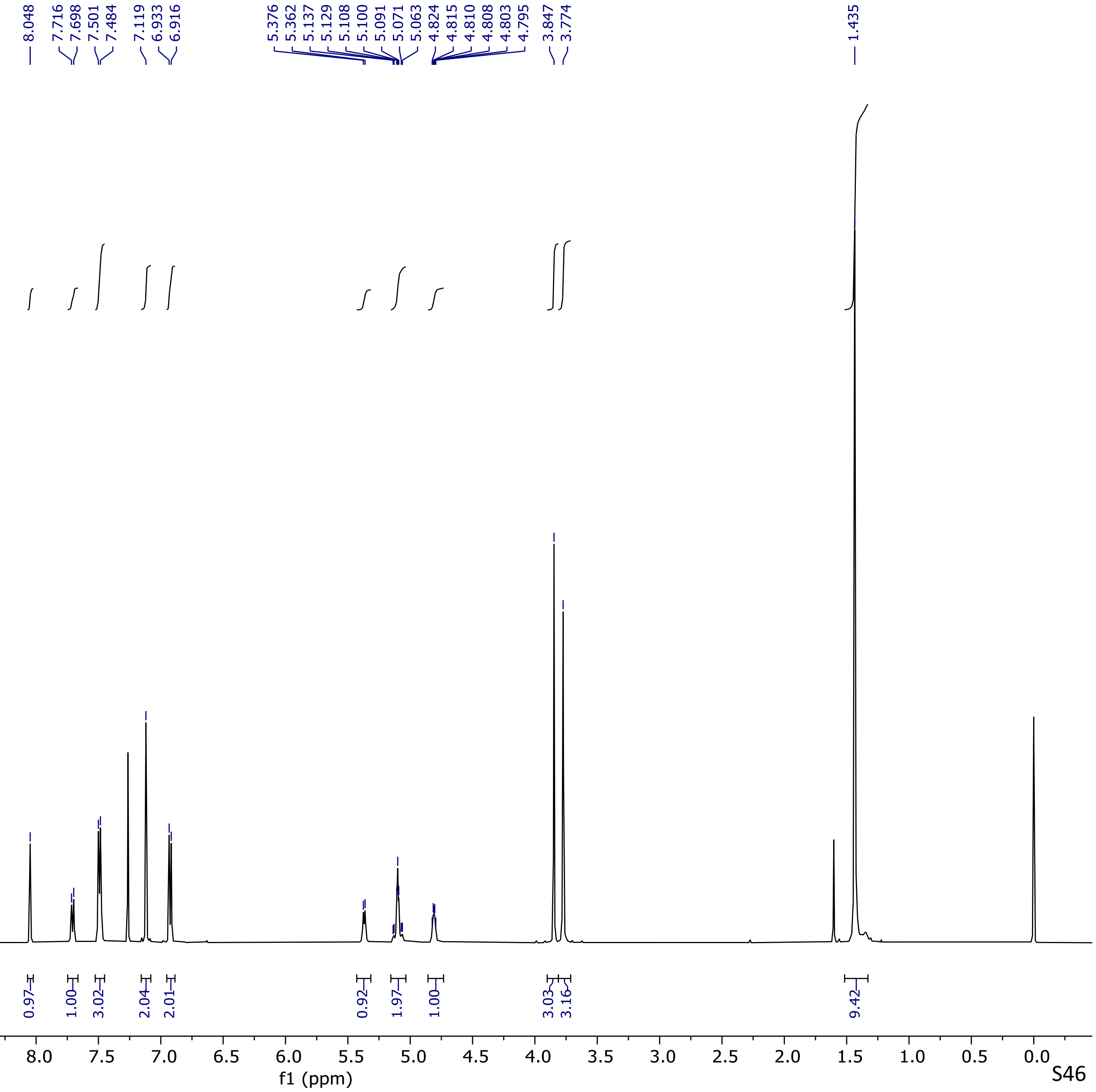

$^{13}C\{^{1}H\}$ NMR (126 MHz, $CDCl_3$)

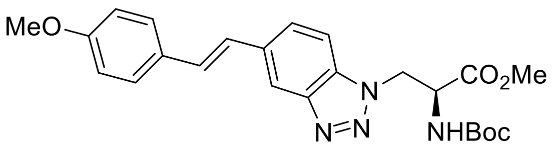

13b

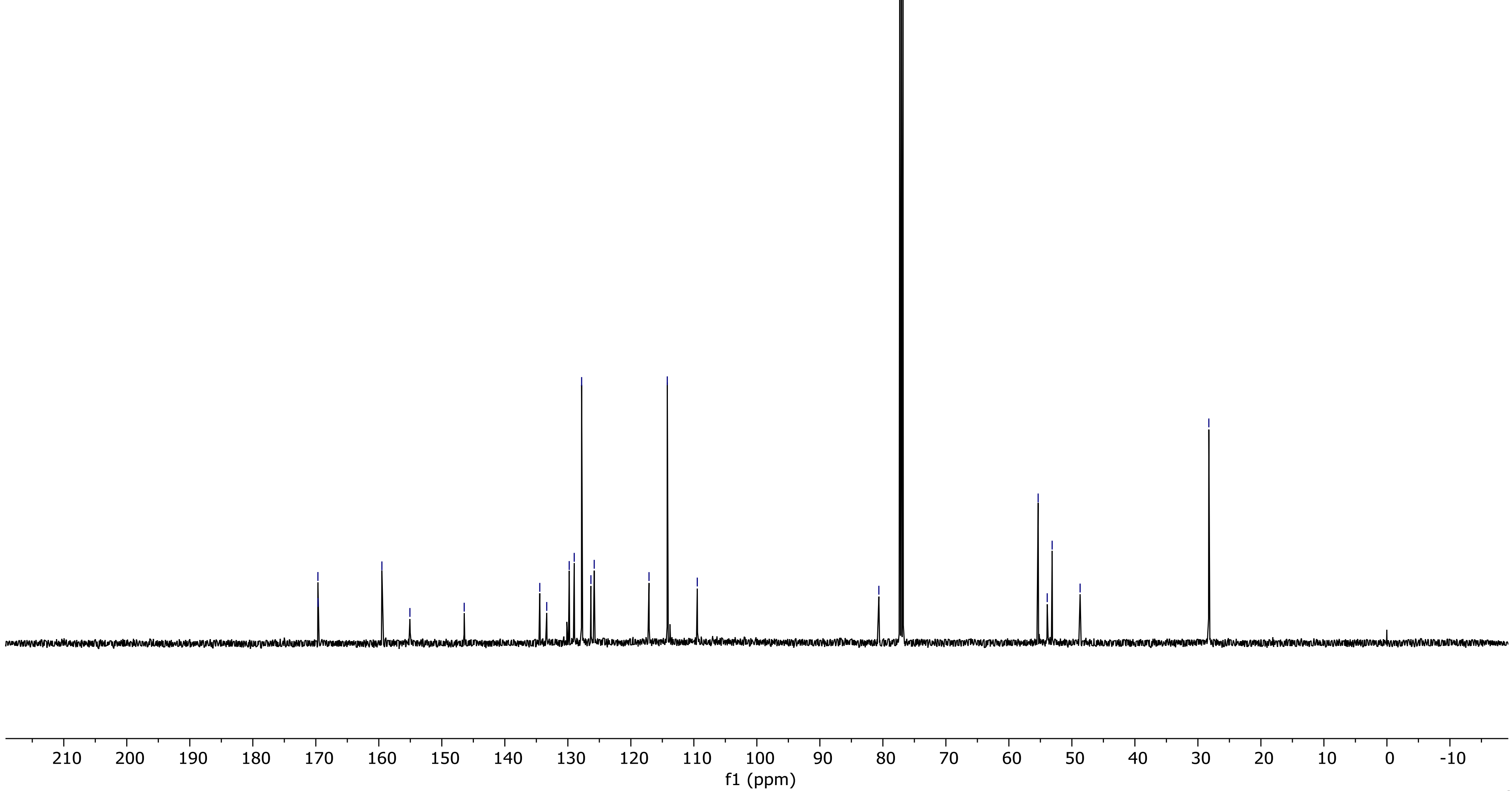

^{1}H NMR (400 MHz, DMSO-d_6)

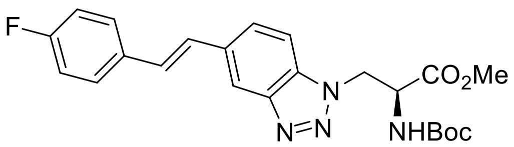

13c

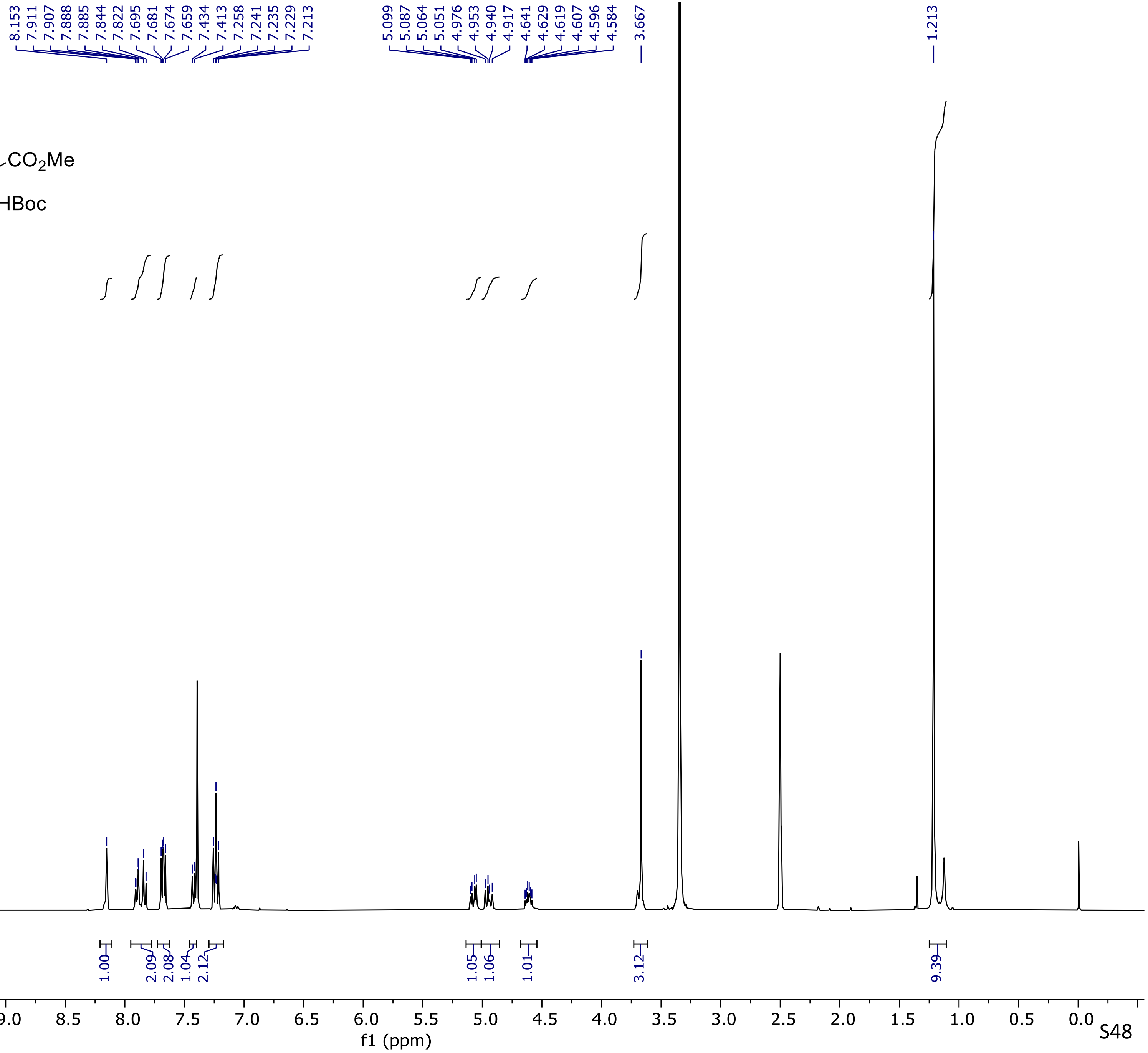

$^{13}C\{^1H\}$ NMR (101 MHz, DMSO-d_6)

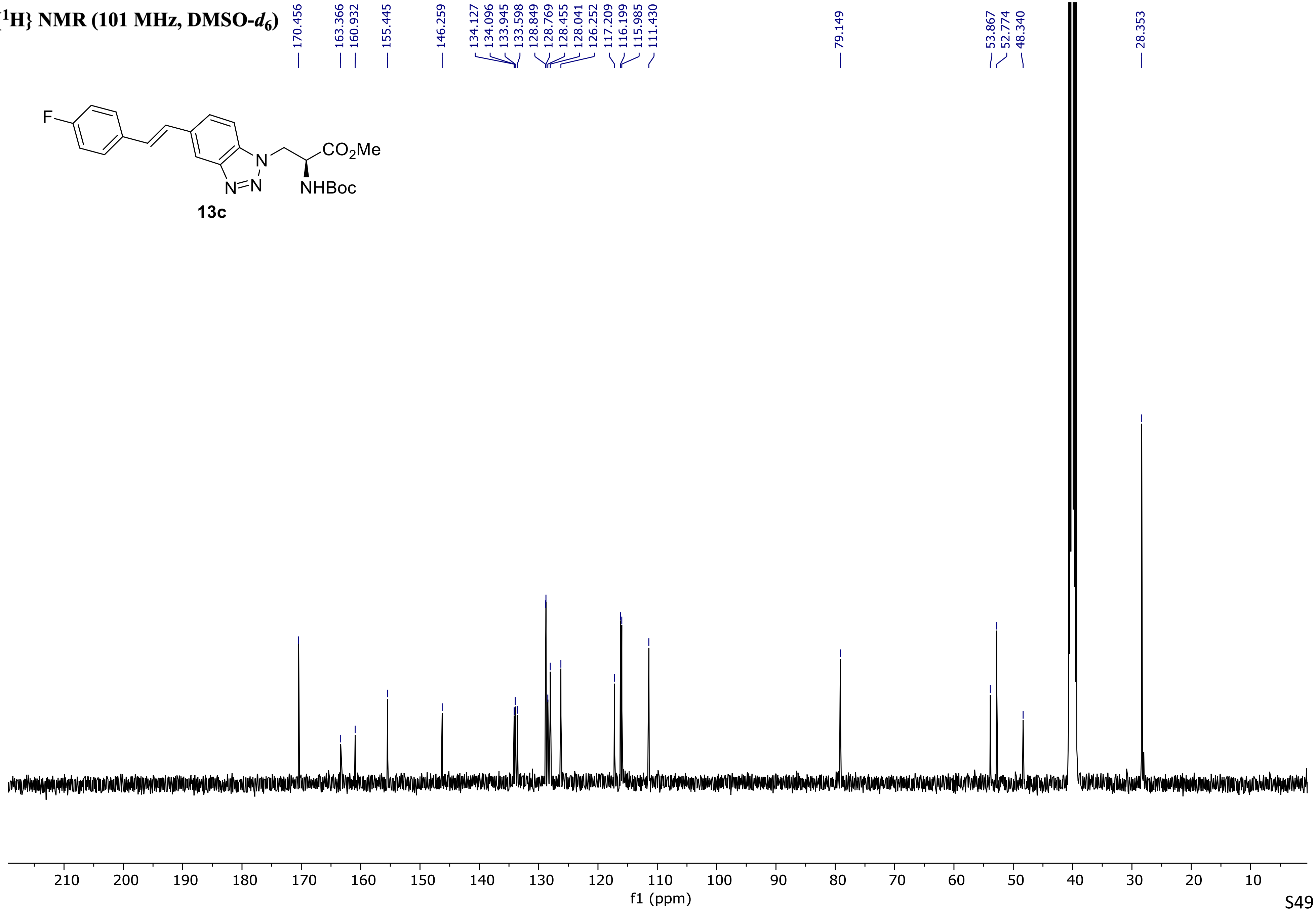

^{1}H NMR (500 MHz, CD_3OD)

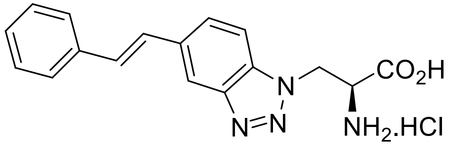

14a

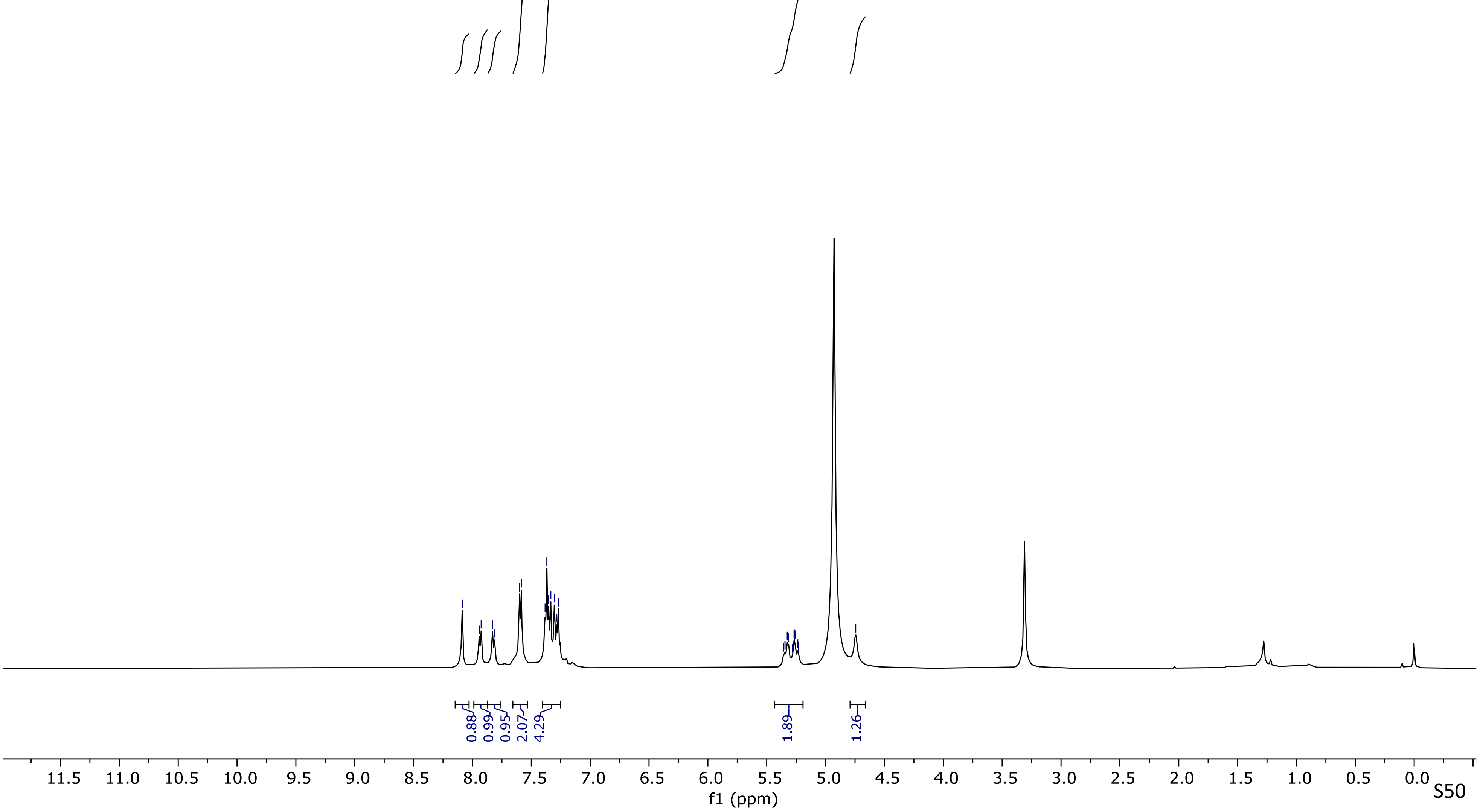

$^{13}C\{^{1}H\}$ NMR (126 MHz, CD_3OD)

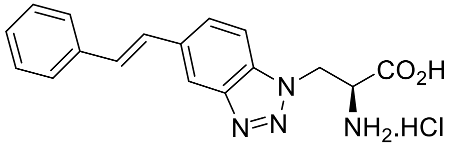
14a

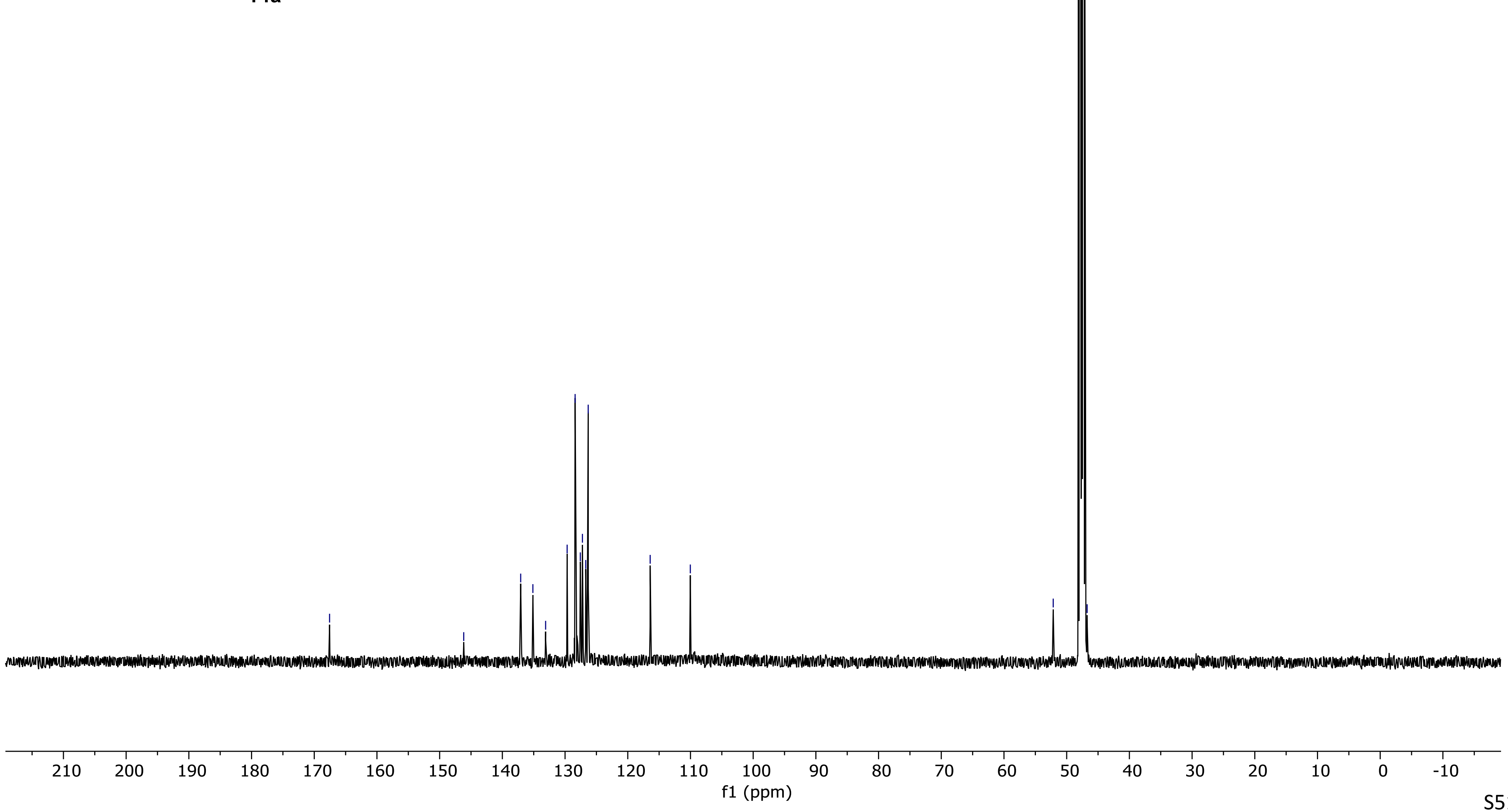

^{1}H NMR (400 MHz, DMSO-d_6)

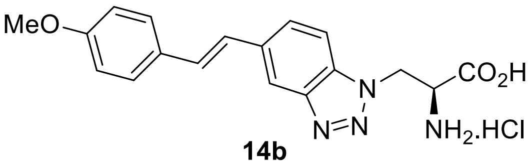

14b

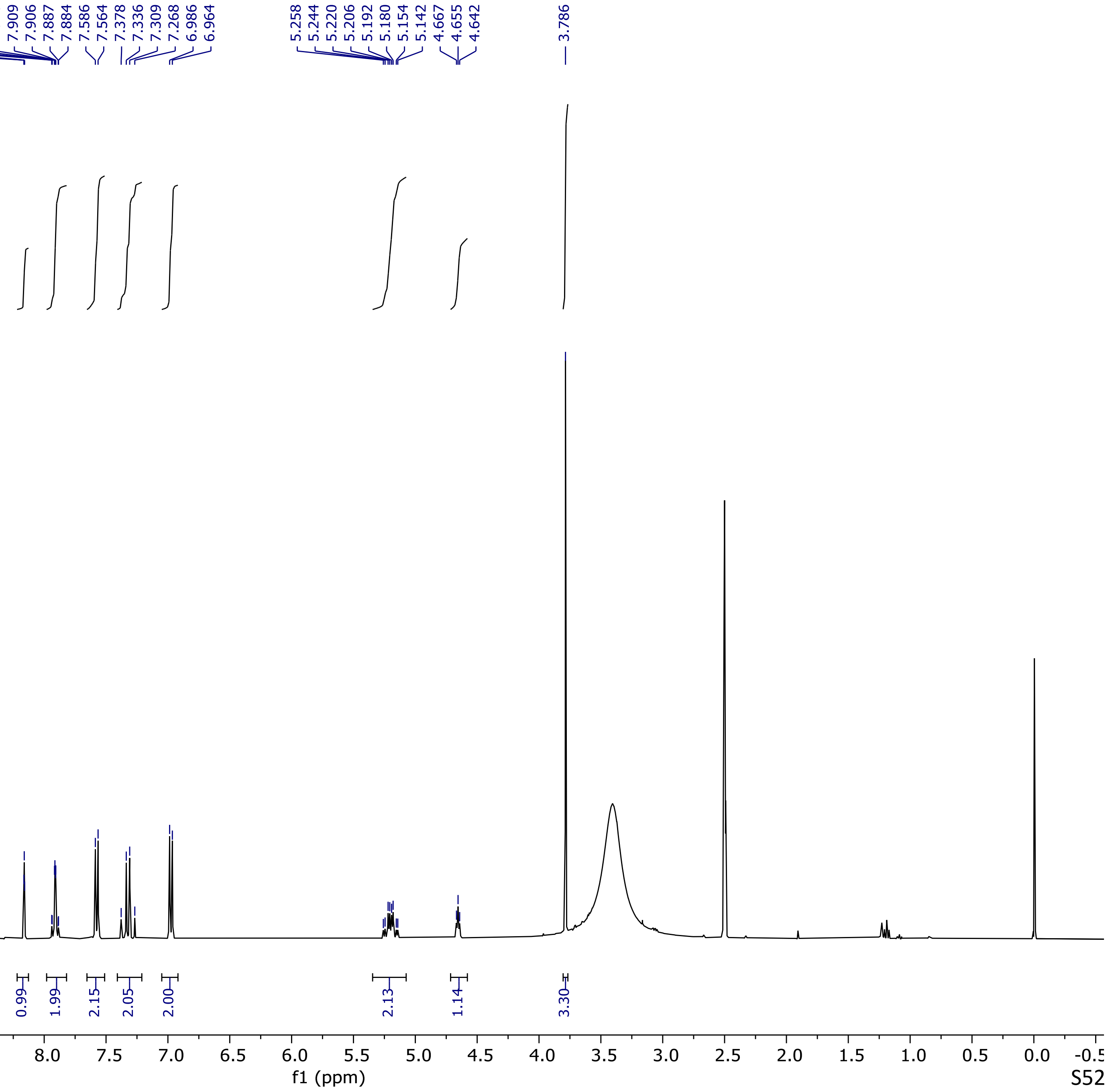

$^{13}C\{^{1}H\}$ NMR (101 MHz, DMSO-d_6)

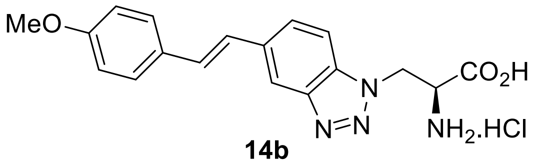

14b

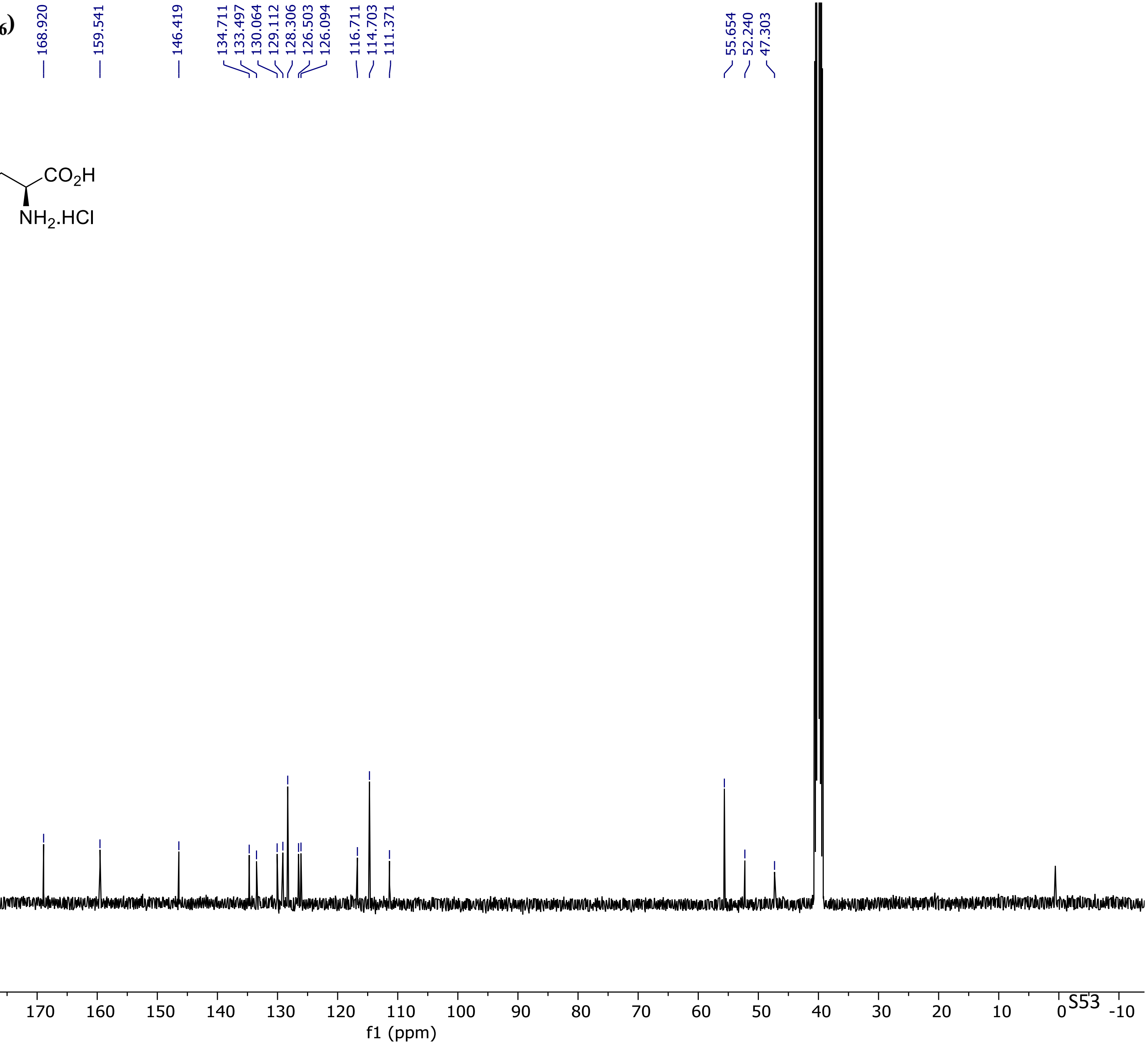

^{1}H NMR (500 MHz, DMSO-d_6)

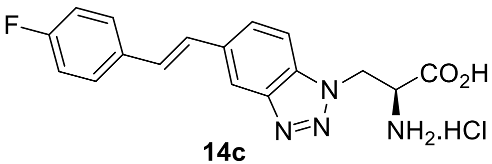

14c

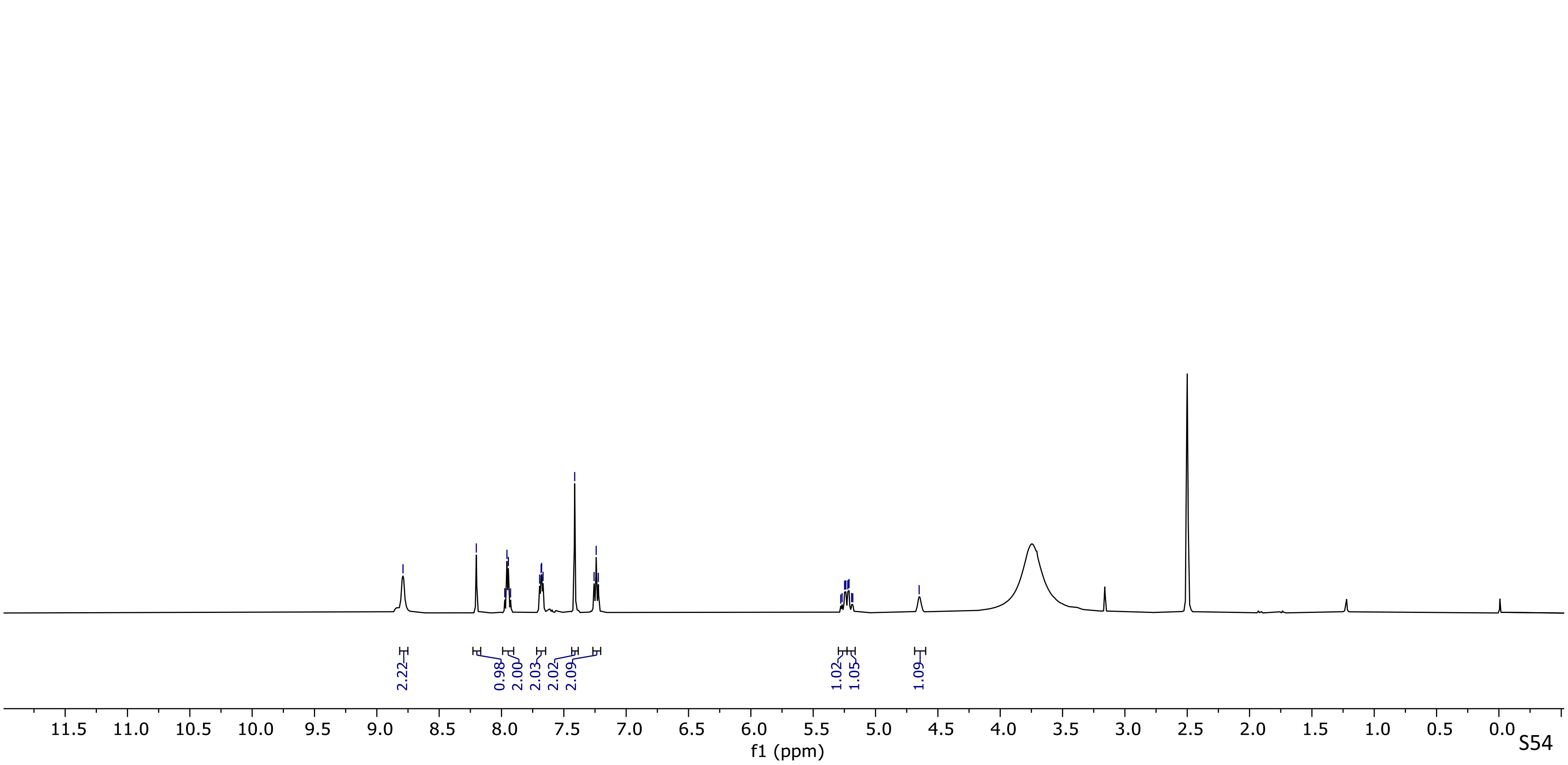

$^{13}C\{^{1}H\}$ NMR (126 MHz, DMSO-d_6)

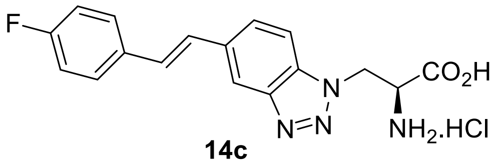

14c

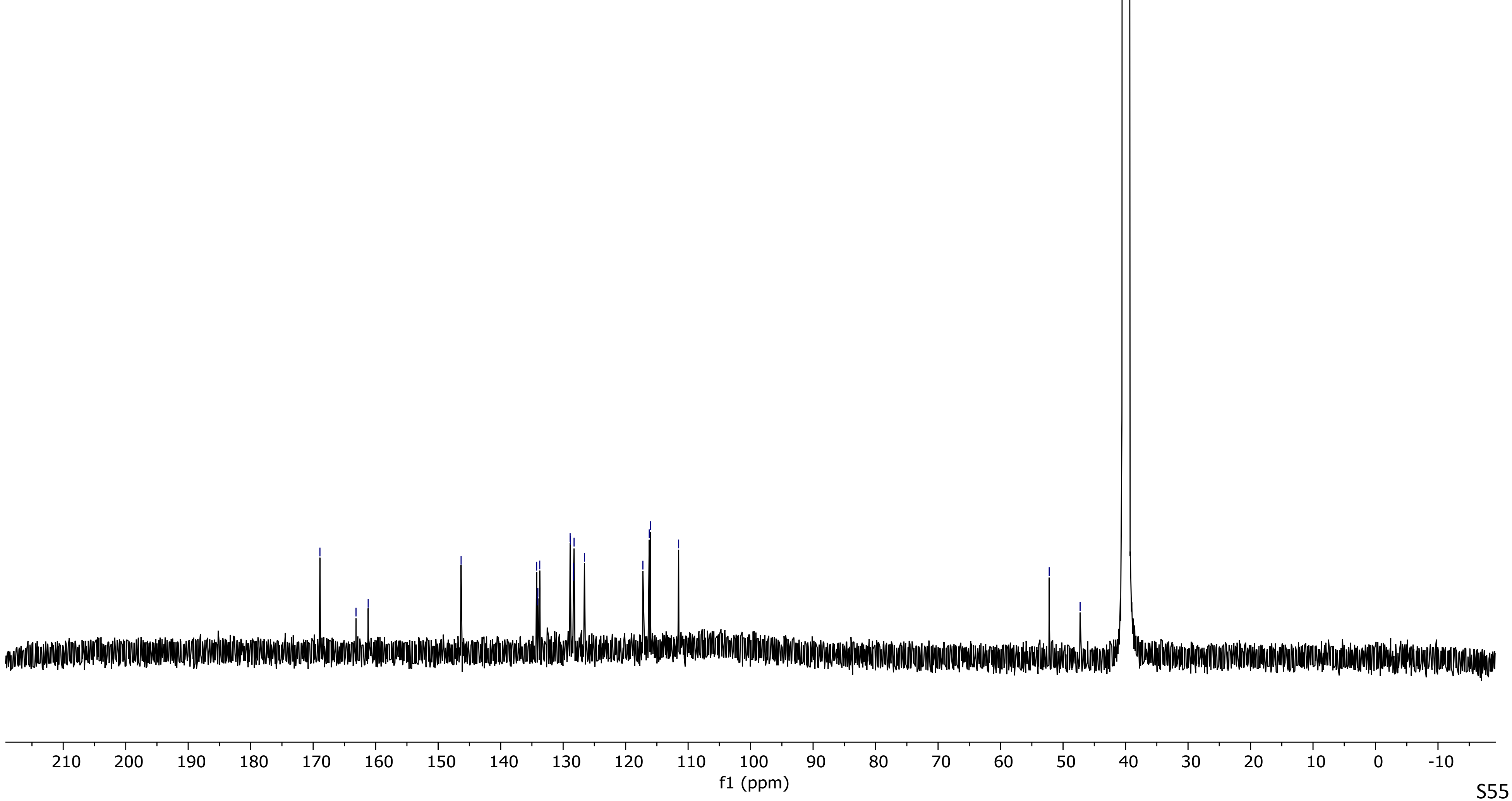

Supplement: Supplementary file 1 — jo2c02886_si_001.pdf [file jo2c02886_si_001.pdf]
